# Oxygen-oxygen bond cleavage enables efficient photocatalytic H_2_O_2_ production via an *O_2_ dissociation pathway

**DOI:** 10.1038/s41467-026-73685-x

**Published:** 2026-06-05

**Authors:** Qiong Liu, Tianxiang Chen, Tsz Woon Benedict Lo, Fuxian Wang, Gangfeng Ouyang

**Affiliations:** 1 College of Environment and Climate, Guangdong Provincial Key Laboratory of Environmental Pollution and Health, Jinan University, Guangzhou, China; 2 Institute of Analysis, Guangdong Academy of Sciences (China National Analytical Center, Guangzhou), Guangzhou, China; 3 Department of Applied Biology and Chemical Technology, The Hong Kong Polytechnic University, Hong Kong, China; 4 School of Chemical Engineering and Technology, Sun Yat-sen University, Zhuhai, China; 5 Huaxin Chuangneng (Guangdong) Technology Co., Ltd., Foshan, China

**Keywords:** Photocatalysis, Artificial photosynthesis

## Abstract

Photocatalytic reduction of O_2_ to H_2_O_2_ is generally regarded to proceed through the *O_2_ hydrogenation pathway (*O_2_ → *OOH), which inevitably encounters a high energy barrier proton extraction process via cleavage of the H-O bond in H_2_O. Designing a K and Cs co-modified polymeric carbon nitride (CN-KCs) to create dual-end adsorption sites as frustrated Lewis pairs for O_2_, on which the O-O breaking energy is significantly reduced and a *O_2_ dissociation pathway towards photocatalytic H_2_O_2_ synthesis is realized (*O_2_ → 2*O, then *O + *H_2_O → H_2_O_2_). The CN-KCs presents a competitive photocatalytic H_2_O_2_ yield of 1806.6 μmolh^−1^, a high quantum efficiency of 72.3% at 420 nm, and a solar-to-H_2_O_2_ conversion efficiency of 6.1% with presence of biomass derivative. Here, we show that regulating dual-end adsorption sites opens a door to new *O_2_ dissociation avenue for efficient conversion of O_2_ to H_2_O_2_.

## Abstract (web summary)

The authors report a dual-site carbon nitride catalyst that enables hydrogen peroxide production via an unconventional O_2_ dissociation pathway, bypassing traditional limits and offering an alternative avenue for sustainable chemical synthesis.

## Introduction

H_2_O_2_, as an environmentally friendly oxidant, plays important role in different fields, such as bleaching, chemical synthesis, sewage treatment, disinfection, and even rocket propellant^1^. In the circumstance of worldwide net-zero transition, photocatalytic oxygen reduction reaction (ORR) has emerged as a promising approach for green and sustainable H_2_O_2_ production^2–5^. Currently, two photocatalytic ORR reaction pathways have been proposed and H_2_O_2_ is considered to be generated through either a one-step two-electron direct reduction (O_2_ + 2H^+^+2e^-^→H_2_O_2_) or two-step single-electron indirect reduction (O_2_+e^-^→·O_2_^-^/*O_2_, then *O_2_ + 2H^+^+e^-^→H_2_O_2_)^1^. Both cases are based on the premise that O-O bond does not break during the photocatalytic ORR reaction, which is essentially valid since so far the activation energy of *O_2_ dissociation (*O_2_ → 2*O) was found to be higher than that of *O_2_ hydrogenation (*O_2_ → *OOH) on most photocatalysts^6^. Therefore, existing researches are limited to reducing the formation energy barrier of *OOH^7,8^, suppressing the O = O bond dissociation. In sharp contrast, no attempt is made to investigate the *O_2_ dissociation (*O_2_ → 2*O) pathway towards photocatalytic H_2_O_2_ production.

Recently, in the field of alkaline-air battery of which the discharge is also based on ORR, the understanding of O_2_ adsorption and activation has been undergoing a profonde change: Li_2_O, due to its’ high energy density, has emerged a strong candidate as the oxidation product compared to its traditional counterparts LiO_2_ and Li_2_O_2_^9^. Noteworthily, unlike the formation of LiO_2_ and Li_2_O_2_ during which the O-O maintains, the Li_2_O formation involves the breaking of the O-O bond of O_2_ during the discharge. Actually, back to the 1970s, Papageorgopoulos et al. has experimentally verified the significant effect of alkaline metals/ions including Na^10^, K^11^, Cs^12^ on O_2_ adsorption. Specifically, the presence of Cs dramatically increases the sticking coefficient and adsorption of O_2_. However, the cleavage of O-O bond was not investigated. Inspired by the classic experimental founding and the latest progress in alkaline-air battery, we proposed the hypothesis that by applying alkaline metal/ions into the photocatalyst, the O_2_ adsorption can be significantly enhanced and it is possible to decrease the energy of *O_2_ dissociation (*O_2_→ *O), cleavage the O-O bond, and thus create a new pathway towards photocatalytic H_2_O_2_ synthesis (*O_2_ → 2*O, then *O + *H_2_O → H_2_O_2_).

In this work, we systematically investigate the effect of alkaline metals/ions doping on the photocatalytic ORR activity of carbon nitride. A K and Cs co-modified polymeric carbon nitride (CN-KCs) is elaborately designed, on which dual strong O_2_ adsorption sites are created and the cleavage of O-O bond is realized. A new photocatalytic ORR pathway towards H_2_O_2_ production through *O_2_ dissociation is proposed, which is demonstrated to be thermodynamically more favorable than the traditional proton-coupled pathways via *OOH formation. A competitive solar-to-H_2_O_2_ conversion efficiency is achieved with high quantum efficiencies under mild photocatalytic conditions.

## Results

### Photocatalytic ORR to H_2_O_2_

**Fig. 1:**
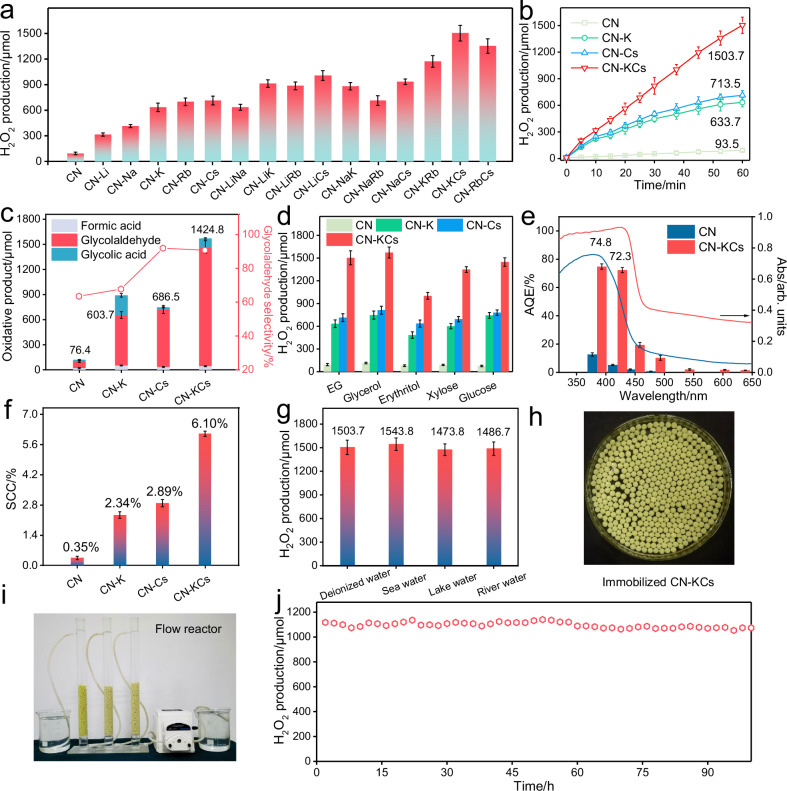
Photosynthesis performance of H_2_O_2_. **a** Photocatalytic H_2_O_2_ generation rates over a series of prepared catalysts for 60 min. **b** The time-dependent photocatalytic reduction to H_2_O_2_ generation rate and **c** Photocatalytic EG oxidation product generation rate using white 10 W LED light source at an intensity of 100 mW/cm^2^ at 25 °C within 20 mL of a 0.5% (v/v) EG aqueous solution over 15 mg of CN, CN-K, CN-Cs and CN-KCs. **d** Comparison of photocatalytic H_2_O_2_ generation rates using different biomass derivative C_2_-C_6_ substrates. **e** Apparent quantum efficiencies (AQE) for H_2_O_2_ product at specific incident wavelengths, together with the DRS spectra of CN and CN-KCs, the curve pointed to by the arrow denotes the DRS spectrum. **f** The solar-to-chemical conversion efficiency (SCC) value. **g** Photocatalytic H_2_O_2_ generation rates over CN-KCs in deionized water, sea water with a K/Na concentration of 0.31 mmol/L, lake water with the total organic carbon (TOC) of 7.3 mg/L, and tap water with the TOC of 5.8 mg/L. **h** The CN-KCs beads immobilized by calcium alginate. **i** The fixed-bed reactor system. **j** Continuous photocatalytic H_2_O_2_ synthesis using the fixed-bed reactor. Error bars represent the standard deviation from three independent replicates.

Our initial experiments focused on evaluating a series of alkali metals, including Li, Na, K, Rb, and Cs, as well as dual alkali metal co-modified polymeric carbon nitride (CN) catalysts for the photocatalytic reduction of O_2_ to synthesize H_2_O_2_. To address the sluggish kinetics of water oxidation half-reaction, we used 0.5% (v/v) ethylene glycol (EG), a typical biomass, as hole scavenger. As illustrated in Fig. 1a, after 1 h LED illumination, all modified CN catalysts showed improved photocatalytic activity for H_2_O_2_ synthesis compared to CN, and the highest activity was achieved on CN-KCs.

Then, we concentrated on CN-KCs as our primary subject for further investigation, the catalyst optimization processes are provided in Supplementary Figs. 1-5. As shown in Fig. 1b, CN yielded an H_2_O_2_ production yield of 93.5 μmol h^−1^. Both CN-Cs and CN-K exhibited significant enhancements in activity, while CN-KCs achieved the highest H_2_O_2_ synthesis yield at 1503.7 μmolh^−1^ (i.e., 100.2 mmol h^−1^ g^−1^ within 20 mL). Concurrently, EG was oxidized into formic acid, glycolaldehyde, and glycolic acid (Fig. 1c and Supplementary Figs. 6-7). Replacing EG (C2) with other biomass-derived compounds also resulted in substantial photocatalytic H_2_O_2_ yield (Fig. 1d and Supplementary Figs. 8–9), indicating the broad applicability of biomass substrates. CN-KCs achieves the highest yield for glycolaldehyde from EG with a high selectivity of 91.9%^13^, consistent with its high ORR activity. Control experiments demonstrated that the catalyst, light source, air, H_2_O, and EG are all essential prerequisites for efficient H_2_O_2_ production (Supplementary Figs. 10–11). When N_2_ was used instead of air, no H_2_O_2_ was produced, excluding the water oxidation (WOR) pathway for H_2_O_2_ production. Therefore, the H_2_O_2_ is synthesized exclusively from the reduction of O_2_. When anhydrous solvents were used instead of H_2_O (Supplementary Fig. 12), the reaction was largely ineffective, highlighting the necessity of the H_2_O involved process for H_2_O_2_ generation. Further control experiments (Supplementary Fig. 13) reveal that a high yield of H_2_O_2_ can only be achieved through the formation of the chemical bonding between CN and KCl/CsCl.

In line with its high activity, CN-KCs achieved maximum quantum efficiencies of 74.8% and 72.3% at 380 nm and 420 nm, respectively (Fig. 1e, the detailed calculation process could be found in Supplementary Note 1). The variation in quantum yield decreased with increasing wavelength of spectral absorption, indicating that the reaction is fundamentally driven by light. Furthermore, we employed an AM 1.5 light source to drive this dual-functional reaction (Supplementary Fig. 14), CN-KCs reached a high H_2_O_2_ yield of 1806.6 μmol h^−1^, achieving a solar-to-chemical conversion efficiency (SCC) of 6.1% (Fig. 1f). As summarized in Table S1 (Supplementary Note 2)^14–19^, our CN-KCs under air is competitive with state-of-the-art photocatalysts in terms of SCC and AQE. We acknowledge that this comparison may not be entirely fair, as corresponding data with 0.5% (v/v) EG for other catalysts is unavailable. When dispersing the catalysts into 5 mmol aqueous H_2_O_2_ solution (Supplementary Fig. 15), the CN-Cs and CN-KCs systems can effectively minimize the side reaction of H_2_O_2_ overoxidation during the photocatalytic process. The H_2_O_2_ production rate kept unchanged after 10 cycles (Supplementary Fig. 16). Additionally, XRD, FTIR, SEM, TEM, and XPS analyses, and ICP-OES (Supplementary Figs. 17-21) confirm that the structure and composition remain unchanged after the reaction, demonstrating high stability of CN-KCs. The photocatalytic activity of CN-KCs for H_2_O_2_ synthesis under real environmental conditions has also been further explored. In seawater, lake water, and river water, CN-KCs achieved H_2_O_2_ yields of 1543.8, 1473.8, and 1486.7 µmol, respectively (Fig. 1g). This demonstrates substantial robustness of CN-KCs against K/Na ions or dissolved natural organic matters. We further loaded the CN-KCs photocatalysts on sodium alginate beads (Fig. 1h–j) to construct a semi-fixed bed reactor for continuous photocatalytic H_2_O_2_ reduction coupling reactions. At a flow rate of 1200 mL/h, CN-KCs maintained stable H_2_O_2_ synthesis activity over 100 h test period, resulting 120 L H_2_O_2_ solution with a concentration of about 1100 μmol/L, demonstrating consistent performance stability and scalability for H_2_O_2_ production.

### Material Synthesis and Characterization

**Fig. 2:**
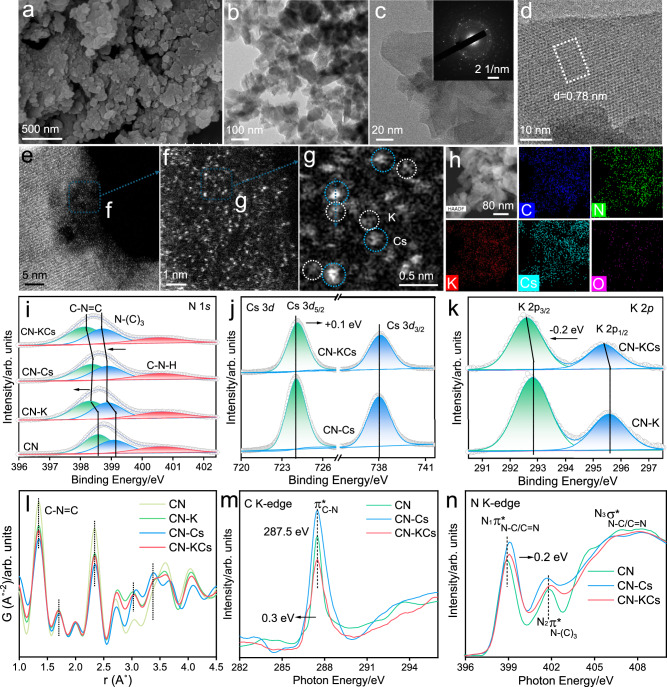
Morphological and structural characterization of CN-KCs. **a** SEM. **b**, **c** TEM, inset is SAED pattern. **d** high resolution-TEM of CN-KCs, the dashed rectangle indicates the area used for measuring lattice spacing. **e**, **f** spherical AC HAADF-STEM image and **g** amplified AC HAADF-STEM image of CN-KCs, the blue dashed rectangle and the corresponding arrow in (**e**, **f)** indicate the magnified area; the arrow in (**g**) indicates the line profile; the blue and white dashed circles represent Cs and K atoms, respectively. **h** HAADF-STEM and elemental mapping images of CN-KCs. XPS spectra of **i** N 1 *s*, **j** Cs 3 *d* and **k** K 2*p*. **l** High-resolution synchrotron X-ray total scattering spectra with refined with Pair Distribution Function of CN, CN-K, CN-Cs and CN-KCs. XANES spectra at the **m** C K-edge and **n** N K-edge. The black dashed lines in (**i**–**n**) indicate the shifting trends of the corresponding peak positions for different catalysts.

The CN-KCs synthesis schematic is shown in Supplementary Fig. 22. The structure of CN-KCs is comprehensively characterized and compared with other samples via the SEM (Fig. 2a and Supplementary Figs. 23–25), TEM image and the selected area electron diffraction (SAED) pattern (Fig. 2b–d and Supplementary Figs. 26–28) to reveal uniform size of the 2D nanosheets and the increase in crystallinity^20^ (related discussion seen below the Supplementary Fig. 23). Crystal lattice or aggregates of K/Cs components is not observed in the high-resolution TEM (HR-TEM) image of CN-KCs. To further investigate the atomic state of K and Cs components, aberration-corrected high-angle annular dark field scanning transmission electron microscopy (AC HAADF-STEM) was employed (Fig. 2e–g). Bright spots densely appear in the dark field images, indicating that K and Cs are dispersed throughout the CN material as individual atoms^20^. Higher magnification images (Fig. 2g) reveal paired bright and larger spots alongside relatively dimmer and smaller spots, which may correspond to Cs and K species, respectively. This observation is supported by the contrast line in Fig. 2g and Supplementary Fig. 29, where the height signal for Cs is higher than that for K^21^. The sizes of the Cs ion and K ion are measured to be 0.168 and 0.139 nm, align with the theoretical values. The corresponding energy dispersive X-ray spectroscopy (EDS) mapping images (Fig. 2h and Supplementary Figs. 30, 31) illustrate a uniform distribution of elements within CN-KCs, CN-K, and CN-Cs. The specific surface area of CN-KCs is measured at 22.4 m^2^/g, with a pore volume of 0.087 m^3^/g, which is slightly smaller than that of pristine CN (Supplementary Fig. 32). Providing that CN-KCs exhibits the highest photocatalytic performance, it can be concluded that surface area is not the main factor that affects the ORR activity. ICP-OES reveals that the contents of K and Cs in CN-KCs are 6.8 wt% and 21.3 wt%, respectively.

CN-KCs retains its characteristic layered structure and forms the K or Cs-Nx conjugated bonding (Supplementary Fig. 33). X-ray photoelectron spectroscopy (XPS) analysis of the C 1 *s* spectra (Supplementary Fig. 34) shows the lower binding energy shift of C-N = C peak after the incorporation of K/Cs species. The XPS N 1 *s* spectrum (Fig. 2i) over CN-KCs deconvolutes into three peaks: C-N = C (N_2_, 398.2 eV), N-(C)_3_ (N_3_, 398.7 eV), and C-N-H_x_ (400.5 eV)^22^. Compared to CN, the binding energies of both N_2_ and N_3_ in modified CN shift to lower values, in accord with the XPS C 1 *s* spectra. This indicates that K and Cs are located within the nitride pots among the three tri-s-triazine units of CN^23^. The location of N_2_ and N_3_ between CN-K with CN-Cs configurations are identical, indicating that K and Cs occupy comparable coordination environments. The Cs 3 *d* binding energy in CN-KCs shifts 0.1 eV to higher value compared to CN-Cs (Fig. 2j). In contrast, the K 2*p* binding energy in CN-KCs shifts 0.2 eV lower than in CN-K (Fig. 2k), probably due to weaker electron-withdrawing effect of Cs.

The changes in atomic environment were further explored through synchrotron radiation total scattering and pair distribution function (PDF) analysis (Fig. 2l and Supplementary Figs. 35–39). The positive peaks among all catalysts are centered at 1.34 Å, 1.70 Å, and 2.34 Å, corresponding to the partial double bonds in the tri-s-triazine network (C = N-C), bridging nitrogen (N-(C)_3_), and the second-neighbor correlation of C-N bonding, respectively^24,25^. The data show that the bond lengths for these three groups are consistent among different catalysts, with variations only in intensity. This demonstrates the tri-s-triazine network structure in CN-KCs remains unchanged compared to CN, consistent with the aforementioned results. In the range of 3.0 to 4.5 Å, this region primarily represents the interlayer distances. CN-K and CN-KCs display a shoulder peak, while CN-Cs shows a distinct peak at 3.37 Å^26^. This indicates that their interlayer correlations may create periodic oscillations at high r values due to the presence of K and Cs atoms, with the Cs atoms partially protruding into the interlayer space as co-verified by XRD. Notably, the PDF data indicate that the fine structure of CN-KCs is more closely aligned with that of CN-K.

We employed synchrotron-based XANES to further investigate the CN-KCs. The C K-edge XANES spectra (Fig. 2m) show a prominent resonance peak at 287.5 eV, indicating electron excitation from C 1 *s* to π* orbitals of s*p*^2^ hybridized C-N bonds^24^. The π* resonance signal of CN-Cs is stronger than that of CN, indicating that Cs donates electrons, enhancing C-N bond polarization. In the N K-edge XANES spectra (Fig. 2n), a signal peak near 401.8 eV corresponds to the transition from the N 1 *s* to the π* state of N-(C)_3_^26^. The introduction of Cs results in a shift of this peak from 401.8 eV in CN to 401.6 eV. This shift is attributed to the localized electron accumulation effect and polarization induced by Cs atom, leading the Cs atom as the Lewis-acidic site and to a decrease in the π* transition energy level over N-(C)_3_. While, the coexistence of K and Cs in CN-KCs leads to a rightward shift of the peak position. This phenomenon is attributed to the smaller atomic radius of K, which weakens the polarization effect on the surrounding electron cloud, thereby raising the energy level of the antibonding orbitals of N-(C)_3_. This balance in polarization may facilitate the adsorption and formation of stable reactive intermediates and asymmetric structural sites.

### Mechanism of *O_2_ dissociation photocatalytic ORR pathway

**Fig. 3:**
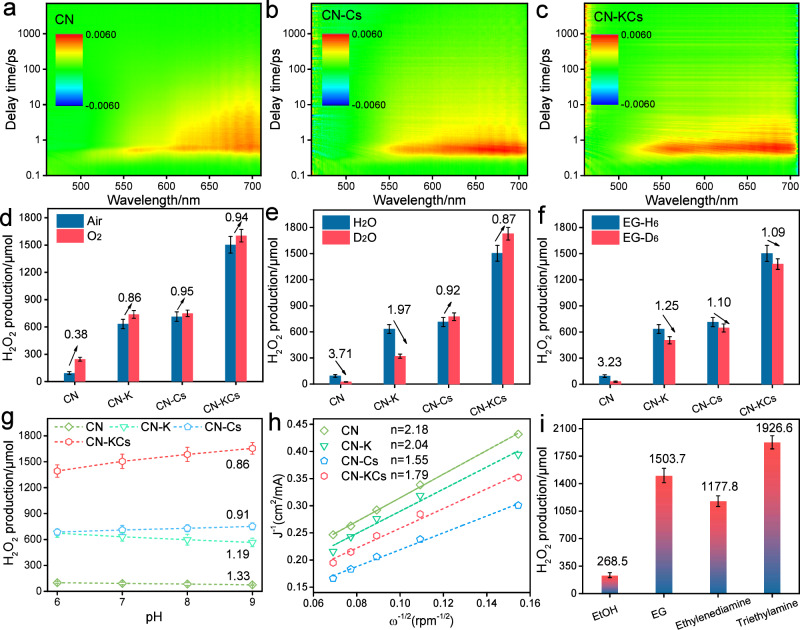
fs-TA spectra and Control experiments. 2D mapping femtosecond transient absorption (fs-TA) spectroscopy measurements of (**a**) CN, **b** CN-Cs and **c** CN-KCs. Comparison H_2_O_2_ generation rate for 60 min (**d**) under air or O_2_ gas, **e** using H_2_O or D_2_O in the reaction system; white 10 W LED light source with 100 mW/cm^2^ at 25 °C within 20 mL of a 0.5% (v/v) EG aqueous solution over 15 mg of CN, CN-K, CN-Cs and CN-KCs, **f** Using EG or deuterated EG-D_6_ as the substrate, the arrows in (**d**–**f)** indicate the activity ratio of the former condition relative to the latter control, and **g** In different pH from 6 to 9 over the different catalyst. **h** The Koutecky–Levich plots, n denotes the average number of electrons. **i** Comparison H_2_O_2_ generation rate using different substrate on CN-KCs. Error bars represent the standard deviation from three independent replicates.

It is revealed the band position in CN-KCs is enough to drive the whole redox reaction (Supplementary Figs. 40–42). In CN-Cs and CN-KCs of the fs-TA spectra (Fig. 3a–c and Supplementary Figs. 43-44), the rapid decay of charge carriers than CN signifies that as the existence of Cs atom, the excited electrons can be swiftly transferred from the heptazine units in CN toward surface-active sites. As such, this could continuously supply excited electrons to facilitate surface reactions for adsorbed O_2_ molecules on CN-KCs. The intensity of excited-state absorption (ESA) in the region of approximately 520–700 nm for CN-Cs and CN-KCs is stronger than that of CN. This indicates a generation of more excited electrons and a broader spectral range of active responses^27,28^, which is consistent with the AQE activities of CN and CN-KCs at different wavelengths.

We further conducted a series of control experiments to gain deeper insights into the reaction mechanism of the oxygen reduction reaction (ORR) for H_2_O_2_ production. Initially, when replacing air with O_2_ (Fig. 3d), the activity of CN, CN-Cs and CN-KCs increased by 163%, 5.26% and 6.38%, respectively. This indicates that incorporating Cs significantly enhances oxygen adsorption on CN^12^ (further confirmed by TPD, vide infra). Next, we conducted isotopic experiments by using D_2_O instead of H_2_O during H_2_O_2_ production (Fig. 3e). The kinetic isotope effect (KIE) value for CN and CN-K is 3.71 and 1.97, respectively, indicating that the protons released from H_2_O play a crucial role in the formation of H_2_O_2_. In this case, the H_2_O_2_ production occurs via a proton-coupled electron transfer (PCET) hydrogenation process, typically considered as the rate-determining step of the ORR^29^, consistent with previous findings. Interestingly, the KIE values for CN-Cs and CN-KCs are 0.92 and 0.87, respectively. This inverse kinetic isotope effect demonstrates that using D_2_O is more effective than H_2_O for H_2_O_2_ synthesis via ORR. Note that the average bond dissociation energy of the O-H bond in H_2_O and D_2_O is 5.18 eV and 5.28 eV, respectively^30^. In other words, the dissociation of O-D is theoretically more challenging than that of O-H. Therefore this inverse kinetic isotope effect^31^ on CN-Cs and CN-KCs suggests that H_2_O molecules do not necessarily undergo the traditional O-H dissociation pathway, but directly couple with oxygen-containing intermediates like *O to produce H_2_O_2_ (*O + *H_2_O → H_2_O_2_). As such, this indicates the introduction of Cs in CN-Cs and CN-KCs may function as Lewis- acidic Cs site to facilitate hydrogen (proton/electron) transfer and the activation of O_2_, tuning the reaction pathway for H_2_O_2_ production. In Fig. 3f, deuterated ethylene glycol (EG-D_6_) was used in place of EG (C_2_H_6_O_2_), revealing typical KIE effect on all catalysts^32^, suggesting EG serves primarily as hole scavenger.

Subsequently, we investigated the activity of the catalysts under different pH (Fig. 3g). For CN-K and CN, the H_2_O_2_ synthesis rates in the proton-rich solution at pH 6 are 1.19 and 1.33 times higher, respectively, than those in the proton-deficient solution at pH 9. This observation suggests an increased concentration of free protons in the solution enhances the activity of the ORR on CN-K and CN, which is reasonable in traditional PCET process. However, for CN-Cs and CN-KCs, the H_2_O_2_ synthesis activity decreases under proton-rich conditions. This implies their mechanism does not rely on the traditional PCET process in which free proton is required, further supporting that a new ORR reaction mechanism occurs on CN-Cs and CN-KCs. What’s more, Koutecky-Levich (K-L) analysis (Fig. 3h and Supplementary Fig. 45)^7^ reveals a decrease in average number of electrons (n) involved in the ORR process diminished from 2.18 for CN to 1.79 for CN-KCs and 1.55 for CN-Cs. This reduction confirms the presence of a single-electron reduction pathway for H_2_O_2_ formation on CN-KCs and CN-Cs, in addition to the conventional two-electron PCET route. In Fig. 3i, two other common hole scavengers, triethylamine and ethylenediamine that do not contain oxygen were used instead of EG. The yield of H_2_O_2_ remained high, suggesting that the oxygen in the produced H_2_O_2_ primarily originates from O_2_ through ORR reaction.

**Fig. 4:**
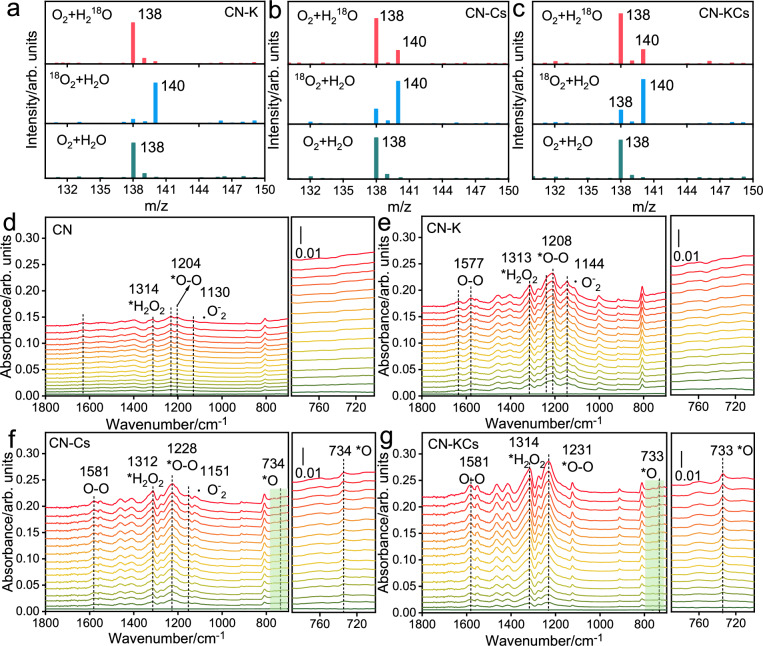
Investigation mechanism of H_2_O_2_ generation. MS spectra of O_2_ derived from H_2_O_2_ using O_2_ + H_2_O, ^18^O_2_ + H_2_O, and O_2_ + H_2_^18^O on (**a**) CN-K, **b** CN-Cs and **c** CN-KCs. In situ ATR-FTIR experiments for (**d**) CN, **e** CN-K, **f** CN-Cs and **g** CN-KCs under the visible light illumination, conducted under a O_2_ gas flow. FTIR spectra were collected at 5-minute intervals, the black dashed lines indicate the corresponding IR peaks.

To further trace the source of oxygen in H_2_O_2_, isotope labeling experiments were conducted (Fig. 4a-c, Supplementary Figs. 46-50). When ^18^O_2_ was used instead of O_2_, all catalysts produced H_2_O_2_ that could yield isotopically labeled ^18^O, via post-treatment with benzoic acid, into p-hydroxybenzoic acid (p-HBA) for GC-MS analysis (see details in Isotope labeling experiments)^33^. A clear shift of the molecular ion peak from m/z = 138 (^16^O-containing p-HBA) to m/z = 140 (^18^O-labeled p-HBA) was observed, confirming that the oxygen atoms in H_2_O_2_ predominantly originate from ^18^O_2_ via the conventional PCET pathway (*^18^O_2_ + 2*H → H_2_^18^O_2_) on CN-K. However, for CN-Cs and CN-KCs, in addition to the dominant m/z = 140 peak, a noticeable signal at m/z = 138 was also observed, with a relative intensity ratio of ~0.30-0.38 (I_138_/I_140_). This result suggests that a fraction of the produced H_2_O_2_ contains oxygen atoms not solely derived from O_2_. Such behavior can be rationalized by the coexistence of an alternative pathway involving surface *O+ *H_2_O pathway, in which *^18^O couples with *H_2_O to form H_2_^16^O^18^O (*^18^O + *H_2_O → H_2_^16^O^18^O).

When replacing H_2_O with H_2_^18^O, the mass spectrometry peak for H_2_O_2_ using CN-K was only a peak at m/z = 138. It can be concluded that the oxygen in H_2_O does not participate in H_2_O_2_ formation using CN-K, with all oxygen in H_2_O_2_ derived from O_2_. This is consistent with the PCET pathway in which the *O_2_ intermediate generates H_2_O_2_. In contrast, for CN-Cs and CN-KCs, an additional peak at m/z = 140 emerged, with a relative intensity ratio of ~0.26–0.34 (I_140_/I_138_). This observation indicates that part of the generated H_2_O_2_ incorporates oxygen from H_2_^18^O, resulting in mixed isotopic products (H_2_^16^O^18^O). This can be attributed to the reaction *^16^O + H_2_^18^O → H_2_^16^O^18^O. Taken together, these results consistently suggest that, in addition to the conventional PCET pathway, an alternative *O-mediated pathway (*O + *H_2_O → H_2_O_2_) operates on CN-Cs and CN-KCs.

Based on a semi-quantitative estimation from the isotopic peak ratios, we infer that approximately 42%–55% of H_2_O_2_ (Supplementary Note 3) is generated via the *O + *H_2_O pathway, while the remaining fraction is produced through the conventional PCET route. We note that this estimation is intended to provide a qualitative assessment of pathway contributions rather than an exact quantification. These it can be inferred that Cs atoms in CN serve as Lewis-acidic center, and thus facilitates the O-O bond dissociation to form *O, which could then directly couple with the adsorbed *H_2_O to form H_2_O_2_. Such O-O bond dissociation is recently discovered in the formation of Li_2_O of Li-O_2_ battery^9^.

We then employed in situ attenuated total reflectance (ATR) FTIR technology to further elucidate the photocatalytic reaction pathway (Fig. 4d-g, and Supplementary Fig. 51). The peaks observed for CN-K at 1577 and 1144 cm^−1^ are attributed to the stretching vibrations of adsorbed O_2_ molecules and ·O_2_^-^ radicals following light excitation^16^. When O_2_ is adsorbed on CN-K, an *O-O bond is formed corresponding to the vibration peak at 1208 cm^−1^, which then undergoes a PCET process to generate the *OOH intermediate with a peak at 1238 cm^−1^. The peak at 1313 cm^−1^ corresponds to * H_2_O_2_, indicating the formation of the final product H_2_O_2_. Similar to CN-K, CN also exhibits FTIR peaks at 1130, 1204, and 1231 cm^−1^, corresponding to ·O_2_^-^, *O-O, and *OOH, respectively. However, the intensity of these peaks is significantly weaker than that of CN-K, and no adsorbed O_2_ molecules were observed. This is ascribed to the weaker capacity for oxygen adsorption and lower H_2_O_2_ production activity on CN.

For CN-KCs and CN-Cs, the peak associated with O_2_ adsorption shifts from 1577 to 1581 cm^−1^. A new peak at 733 cm^−1^ emerges, which is attributed to the bending vibration of monodentate *O intermediate^34^. This observation suggests that the *O-O intermediate undergoes dissociation on CN-KCs and CN-Cs, resulting in the formation of *O. And the intensity of *O on CN-KCs is clearly stronger than CN-Cs. In contrast, the presence of *O was not observed on CN and CN-K. We speculate that the dissociation of *O-O is due to the strong adsorption of O by Cs and the formation of frustrated Lewis pairs on the dual-end Cs-N sites, denoted as Cs*O-O*N. The dissociated *O subsequently couples with *H_2_O to form *H_2_O_2_. Therefore, peaks corresponding to *H_2_O_2_ are also observed on CN-Cs and CN-KCs at 1312 and 1314 cm^−1^, respectively^4^. These findings indicate that all catalysts yield the final H_2_O_2_ product, with CN-KCs demonstrating the most significant intensity of the *H_2_O_2_ FTIR peak, suggesting higher catalytic activity. Conversely, CN exhibits the weakest peak intensity, correlating with the lowest H_2_O_2_ production activity, consistent with previous activity data. Additionally, the FTIR peaks associated with the characteristic intermediates of the ORR demonstrate a gradual increase in intensity with prolonged light illumination in a certain time. In contrast, under dark conditions, there are virtually no corresponding signals (Supplementary Fig. 52), indicating that the observed intermediates peaks for the final H_2_O_2_ production are driven by incident photons. Moreover, when O_2_ was replaced with isotopically labeled ^18^O_2_ (Supplementary Fig. 53), the *O stretching vibration shifted from 733 to 712 cm^−1^, consistent with the isotope effect using the harmonic oscillator model, while no O signal appeared under Ar. These results confirm that the 733 cm^−1^ peak originates from O_2_ reduction, with O atoms in O_2_ directly participating in H_2_O_2_ formation.

**Fig. 5:**
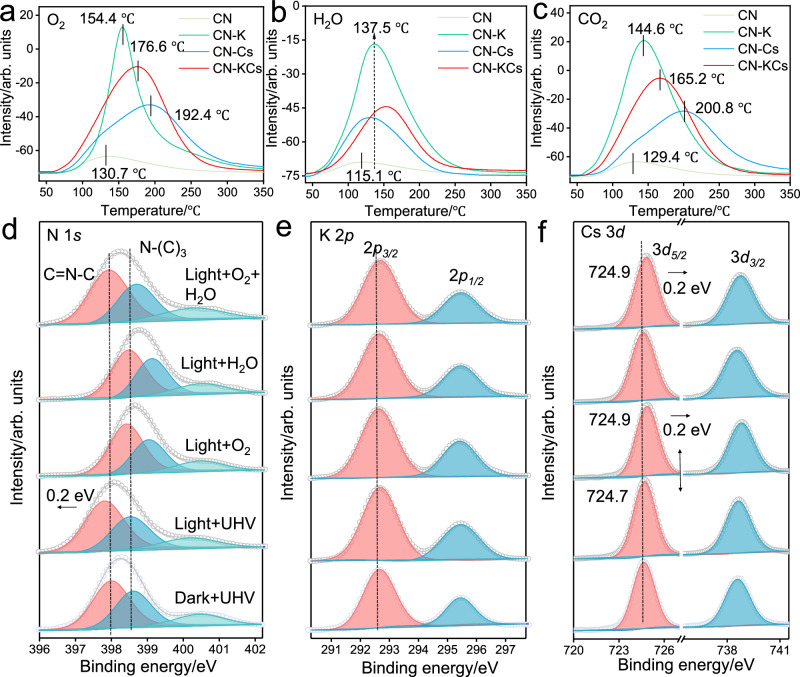
Investigation of surface absorption capacity and chemical state variation. TPD spectra of (**a**) O_2_, **b** H_2_O and **c** CO_2_ on catalysts, black short lines mark the temperatures corresponding to the respective peak positions. In situ XPS experiments of (**d**) N 1 *s*, **e** K 2*p* and **f** Cs 3 *d* spectra under different condition on catalysts, the black dashed lines indicate the reference positions for the shifts of the corresponding XPS peaks; arrows denote the shifts in binding energy for the respective species.

The role of Cs on O_2_ adsorption and activation is further substantiated by temperature-programmed desorption (TPD) and in situ X-ray photoelectron spectroscopy (XPS) analyses. In the TPD-O_2_ curve (Fig. 5a), an enhancement in O_2_ adsorption capacity is observed upon the addition of alkali metals, K and Cs, compared to CN. The O_2_ adsorption peak for CN is located at 130.7 °C. Notably, while the TPD-O_2_ peak temperature for CN-Cs rises dramatically to 192.7 °C, indicating a substantial increase in O_2_ adsorption strength due to the presence of Cs. Further peak fitting of CN-Cs reveals two distinct peaks (Supplementary Fig. 54). Peak 1, which is similar to that of CN, corresponds to pyridinic nitrogen (N_2_) adsorption sites, while peak 2, located at approximately 198.9 °C, is attributed to the O_2_ adsorption sites caused by Cs. This suggests that the incorporation of Cs into CN facilitates the formation of a dual adsorption site configuration (further verified by in-situ XPS). Similar to CN-Cs but with a more pronounced capacity, CN-KCs also exhibits the dual Cs-N_2_ sites configuration (Supplementary Fig. 54) that enhances strong O_2_ adsorption^35^. Moreover, in CN-KCs, the presence of dual adsorption sites increases the likelihood of simultaneous adsorption of each one oxygen atom in O_2_ by both Cs and N_2_, resulting in a bridging adsorption configuration of Cs*O-O*N_2_. This configuration, due to the polar interactions between the Cs and N_2_ dual sites, promotes the dissociation of *O-O, generating the critical *O intermediate in consistent with ATR-FTIR results. In contrast, for CN-K, it is challenging to fit the peaks into two distinct components, and its adsorption peak position remains close to that of CN. This indicates that the presence of K tends to enhance the inherent N_2_ adsorption sites of CN for O_2,_ further illustrated the reason that the FTIR peak intensity of *O on CN-KCs is stronger than CN-Cs. This may explain why the reaction pathways for H_2_O_2_ generation via ORR are similar for CN-K and CN, while CN-Cs and CN-KCs exhibit the dissociation of *O-O pathway. Regarding H_2_O adsorption (Fig. 5b)^36^, modified CN show enhanced H_2_O adsorption capacity than CN. Unlike the TPD-O_2_ adsorption, it is difficult to fit the H_2_O adsorption into two peaks, suggesting that the adsorption sites for H_2_O remain as pyridinic nitrogen (N_2_) and with the introduction of K/Cs enhancing the N_2_ site’s capacity for H_2_O adsorption. The TPD-CO_2_ peak, reflecting the Lewis basic sites^37^, significantly increased to 200.8 °C for CN-Cs, further demonstrating that Cs may act as a new active site with strong adsorption capabilities.

We further conducted in situ XPS experiments to further verify the adsorption sites of CN-KCs. In Fig. 5d, under ultra-high vacuum (UHV) conditions, the binding energy of C = N-C (N_2_) in the XPS N 1 *s* spectrum shifts to lower binding energy by nearly 0.2 eV upon light illumination. In contrast, the binding energy of N-(C)_3_ (N_3_) remains relatively unchanged^20^. This indicates that upon light excitation, the excited carriers generated within the tri-s-triazine network predominantly accumulate at the N_2_ sites, signifying that these are intrinsic active sites. The accumulation of excited electrons reduces the binding energy of the outer electrons of N_2_ sites, leading to a decrease in binding energy. However, upon introducing O_2_ (light+O_2_) or H_2_O (light+H_2_O), the binding energy of the N containing functional groups substantially increases, indicating that the excited carriers are swiftly transferred to O_2_ or H_2_O, forming reactive intermediates and drawing electrons from the original N 1 *s* surface. In the presence of simultaneous light, O_2_, and H_2_O (light+O_2_ + H_2_O), redox reactions can occur, enabling the excited intermediates generated from O_2_ or H_2_O to react and be consumed. In this scenario, continuous light exposure may result in excess excited carriers, leading to a leftward shift in binding energy compared to light+O_2_ or light+H_2_O alone. These results were further confirmed by comparing experiment results between the dark and light time under different conditions (Supplementary Figs. 55–57).

In the XPS K 2*p* spectra, no significant changes in the K 2*p* peak position are observed under different conditions, indicating that excited carriers do not migrate onto K species, further confirming that the introduction of K does not create active sites (Fig. 5e). In contrast, a shift to higher binding energy by nearly 0.2 eV in the XPS Cs 3 *d* spectra (Fig. 5f) is observed under light+O_2_ and light+O_2_ + H_2_O conditions (724.9 eV), compared to the light+UHV condition (724.7 eV). This suggests a strong effect of Cs on O_2_ adsorption. The excited electrons near Cs are captured by O_2_, thereby enhancing the interaction between Cs and O atoms. Conversely, under light+H_2_O conditions, the Cs 3 *d* peak remains unchanged, indicating minimal interaction between Cs and H_2_O, which is consistent with TPD results. Thus, the in situ XPS spectra further verify the presence of frustrated Lewis pair (FLP) on dual adsorption sites of Cs-N_2_ for O_2_ on CN-KCs, on which the dual-end adsorption of O_2_ is strong enough to trigger the dissociation of the O-O bond to monodentate *O as jointly confirmed by ATR-IR tests.

**Fig. 6:**
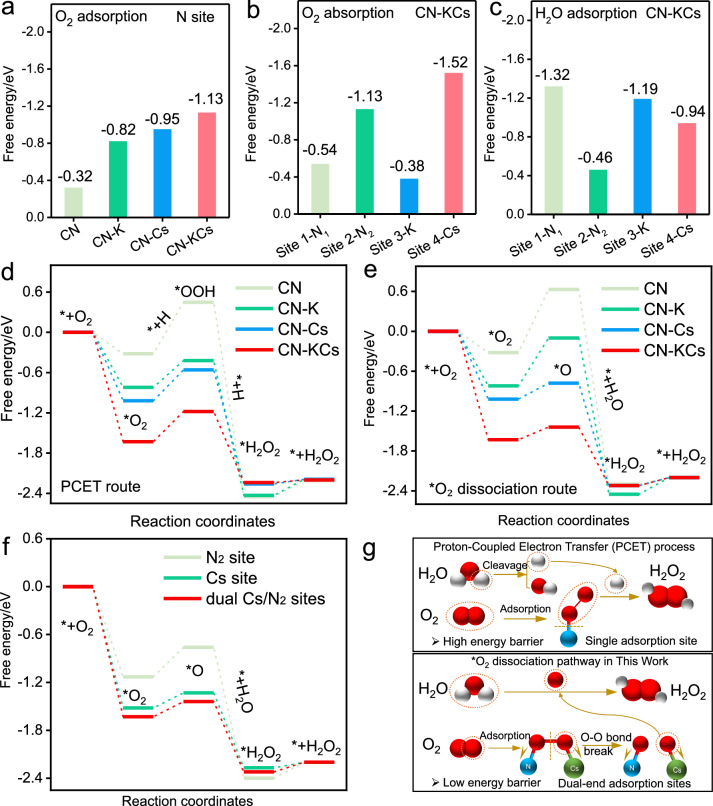
Investigation of energy barrier during H_2_O_2_ generation. DFT Energy barrier for (**a**) O_2_ adsorption on catalysts. **b** O_2_ adsorption over different sites on CN-KCs and **c** O_2_ adsorption on catalysts. Calculated free energy diagram from O_2_ to H_2_O_2_ via (**d**) the PECT route or **e** *O_2_ dissociation pathway on different catalysts and **f** the different sites on CN-KCs, dashed lines indicate the transitions between reaction intermediates, while horizontal solid lines represent specific reaction intermediate states. **g** Proposed reaction mechanism for photocatalytic H_2_O_2_ production via the *O_2_ dissociation pathway and comparison with the typical proton coupled electron transfer (PCET) process.

The enhanced oxygen adsorption on CN-KCs is further complemented by Density functional theory (DFT) calculations (Fig. 6a and Supplementary Figs. 58–61, Supplementary Data 1). The O_2_ adsorption energy on CN is calculated to be −0.32 eV, indicating a weak physical adsorption. Whereas a much higher O_2_ adsorption energy of −0.82, −0.95, −1.13 eV is obtained on CN-K, CN-Cs, and CN-KCs respectively (Fig. 6a), suggesting a trend of strengthened adsorption of O_2_ on KCs. Further investigation into the O_2_ adsorption energy on CN-KCs points to Cs as the most favorable adsorption site with a high adsorption energy of −1.52 eV (Fig. 6b), followed by the pyridinic nitrogen (N_2_) site near Cs with an adsorption energy of −1.13 eV. This results suggest that both Cs and Cs coordinated N_2_ sites exhibit strong O_2_ adsorption, while K itself shows weak O_2_ adsorption. And the stronger H_2_O adsorption site is located at site N1 (Fig. 6c). The reaction pathway from O_2_ to H_2_O_2_ formation was further explored, as depicted in Fig. 6d–f and Supplementary Data 2. The energy barrier for the transition from *O_2_ to *OOH via the proton-coupled electron transfer (PCET) process on different catalyst is endothermic^18^, representing the rate-determining step in the oxygen reduction reaction (ORR) to H_2_O_2_. However, with the incorporation of K or Cs species, the energy barrier is clearly lowered from 0.77 eV on CN to 0.51, 0.46, and 0.45 eV on CN-K, CN-Cs, and CN-KCs, respectively. This suggests that the introduction of K and Cs may promote the PCET reaction pathway^38^.

Notably, for CN-Cs and CN-KCs, the energy barrier for *O_2_ dissociation to *O is predicted to be lower compared to CN and CN-K (Fig. 6e). Unlike CN and CN-K possessing only single N_2_ site, CN-Cs and CN-KCs enjoying Cs-N_2_ dual-sites exhibit a lower energy barrier for the *O_2_ dissociation pathway compared to the PCET pathway to *OOH in our models, indicating that the introduction of Cs creates a O_2_ dissociation pathway towards H_2_O_2_ synthesis. In contrast, CN and CN-K are thermodynamically more inclined to produce H_2_O_2_ through the PCET pathway. DFT calculations further performed on the N_2_ site, Cs site, and Cs-N_2_ dual-site on CN-KCs (Fig. 6f) suggest that the Cs-N_2_ dual-site exhibits the lowest reaction energy barrier through a favorable thermodynamic process, further promoting the O_2_ dissociation pathway for enhancing the efficiency of H_2_O_2_ synthesis. Noted that while providing theoretical guidance, these idealized models may not fully account for the dynamic evolution and solvent effects of the real catalytic process.

Based on the above results, it is noteworthy that for CN and CN-K, the ORR pathway for H_2_O_2_ production follows the PCET route, where O_2_ is transformed into *O_2_ (as discussed vide infra), subsequently forming *OOH and *H_2_O_2_ via the following Eqs. 1–3^39^:

1 $${{{\rm{O}}}}_2+\ast \to*{{{\rm{O}}}}_2\,({{\rm{on}}}\,{{\rm{single}}}\,{{\rm{N}}}\,{{\rm{site}}})$$

2 $$*{{{\rm{O}}}}_2+{{{\rm{H}}}}^{+}+{{{\rm{e}}}}^{-}\to*{{\rm{OOH}}}$$

3 $$*{{\rm{OOH}}}+{{{\rm{H}}}}^{+}+{{{\rm{e}}}}^{-}\to*{{{\rm{H}}}}_{2}{{{\rm{O}}}}_{2}$$

However, for CN-Cs and CN-KCs, the presence of Cs introduces a new oxygen dissociation pathway. The formed *O_2_, due to its strong adsorption, tends to dissociate to form *O, which then directly couples with *H_2_O to generate *H_2_O_2_ following Eqs. 4–9:

4 $${{{\rm{O}}}}_2+2*\to*{{\rm{O}}}-{{\rm{O}}}*\,({{\rm{on}}}\,{{\rm{double}}}\,{{\rm{N}}}+{{\rm{Cs}}}\,{{\rm{sites}}})$$

5 $$*{{\rm{O}}}-{{\rm{O}}} \ast+{{{\rm{e}}}}^{-}\to*{{\rm{O}}}+{{{\rm{O}}}}^{-}$$

6 $$*{{\rm{O}}}+\ast {{{\rm{H}}}}_{2}{{\rm{O}}}\to*{{{\rm{H}}}}_{2}{{{\rm{O}}}}_{2}$$

7 $${2{{\rm{O}}}}^{-}+\ast {{{\rm{H}}}}_2{{\rm{O}}}\to*{{{\rm{H}}}}_{2}{{{\rm{O}}}}_{2}+2{{{\rm{OH}}}}^{-}$$

8 $${{\rm{HO}}}-{{{\rm{CH}}}}_2-{{{\rm{CH}}}}_2{{\rm{OH}}}({{\rm{EG}}})+{2{{\rm{h}}}}^{+}\to {{\rm{HO}}}-{{{\rm{CH}}}}_2-{{\rm{CHO}}}+2{{{\rm{H}}}}^{+}$$

9 $${2{{\rm{OH}}}}^{-}+2{{{\rm{H}}}}^{+}\to 2{{{\rm{H}}}}_{2}{{\rm{O}}}$$

Based on which, we propose a plausible mechanism for the photocatalytic ORR to H_2_O_2_ via a *O_2_ dissociation pathway, as illustrated in Fig. 6g.

## Discussion

In summary, we successfully achieved *O_2_ dissociation pathway for the photocatalytic oxygen reduction to H_2_O_2_ with simultaneous EG oxidation on the dual Cs and K atoms co-functionalized carbon nitride. The CN-KCs offers a high photocatalytic H_2_O_2_ yield of 1806.6 μmolh^−1^ with a maximum quantum efficiency of 74.8% and 72.3% at 380 nm and 420 nm, respectively, and exhibits a solar-to-H_2_O_2_ conversion efficiency of 6.1% under ambient conditions, competitive with all the state-of-art photocatalysts. It is disclosed that both the K and Cs increase the oxygen absorption capacity of CN. Specifically, the introduction of Cs significantly strengthens the attraction of nearby N on oxygen, and thus leads to the formation of frustrated Lewis pair (FLP) on dual-end Cs-N_2_ sites for O_2_ adsorption. Compared to the traditional PCET H_2_O_2_ synthesis route with a high endothermic proton extraction process, the CN-KCs with dual-end Cs-N_2_ configuration enables easy dissociation of *O_2_ into *O intermediate and facilitates the consequent coupling of *O with *H_2_O to yield the final H_2_O_2_ product. This work demonstrates the *O_2_ dissociation pathway to produce H_2_O_2_ for the first time and emphasizes the importance of regulating dual-end adsorption sites for efficient ORR.

## Methods

### Materials

Dicyandiamide (C_2_H_4_N_4_, analytical reagent-AR), lithium chloride (LiCl, 99.5%, anhydrous), sodium chloride (NaCl, 99.5%, AR), potassium chloride (KCl, 99.5%, AR), rubidium chloride (RbCl, 99%), cesium chloride (CsCl, 99%), ethanol (C_2_H_5_OH, EtOH, AR), triethylamine (TEA, ≥99.0%, AR), ethanediamine ( ≥ 99.0%, AR), Ethylene glycol (EG, C_2_H_6_O_2_ ≥ 99.9%, AR), Ethylene glycol-d6 (C_2_D_6_O_2_, D, 99%), horseradish peroxidase (HRP, 250 u/ mg) were purchased from Aladdin Bio-Chem Technology Co.,LTD. Deuterium oxide (D_2_O, D, 99.9%), Glycerol (C_3_H_8_O_3_, 99%, AR), D-Erythrose (C_4_H_8_O_4_, 98%), D-Xylose (C_5_H_10_O_5_, 98%), D-Glucose (C_6_H_12_O_6_, 99%) were obtained from Energy Chemical. Glycolic acid (C_2_H_4_O_3_, 97%), N, N-diethyl-p-phenylenediamine (DPD, 99%), Dipotassium hydrogen phosphate (K_2_HPO_4_, AR, 99%), Monopotassium phosphate (KH_2_PO_4_, AR, 99.5%), Manganese dioxide (MnO_2_, AR, 99.5%) were purchased from Macklin Chemical. 5,5-dimethyl-1-pyrroline-N-oxide (DMPO), Heavy-oxygen water (H_2_^18^O, D, 99.7%) was provided from Sigma-Aldrich Chemical Co. High purity nitrogen (Ar, ≥99.9999%), oxygen-^18^O labeled gas (^18^O_2_, ≥99%), high purity oxygen (O_2_, ≥99.9999%), high purity carbon dioxide (CO_2_, ≥99.9999%) and helium gas (He, ≥99.999%) were purchased from Air Liquide. Holey Formvar stabilized with Carbon used for TEM image was purchased from Zhongjingkeyi (Beijing) Film Technology Co.,Ltd. All materials were employed without additional purification.

### Catalyst synthesis

Synthesis of CN-KCs**:** 10 g of dicyandiamide (DCDA) were placed in a ceramic crucible and transferred to a tube furnace. It is directly endured the thermal polymerization process of which the temperature was raised to 570 °C at a heating rate of 7 °C/min under an Ar atmosphere and held for 4 hours. After natural cooling, the resulting yellow bulk entity was ground into a fine powder using a milling machine and denoted as CN. Subsequently, 2 g of CN was dispersed in 30 mL of water and stirred for 15 min. A specific amount of KCl and CsCl (0.8 g KCl + 0.8 g CsCl) was added, followed by 10 min of ultrasonication and 6 h of stirring at 50 °C. The resulting suspension was then subjected the freeze-dried process to remove water. The obtained mixed solid was ground thoroughly and evenly spread on an open porcelain boat. The boat was placed in a tube furnace and heated to 550 °C at a heating rate of 7 °C/min under an Ar atmosphere for 4 h. After natural cooling, the sample turned green and was washed repeatedly with water to remove residual metal salts. The sample was then dried at 100 °C in an oven and finally obtained as a K and Cs-containing sample, denoted as CN-KCs. For comparative studies, a series of additional CN samples modified with binary alkali metals, including CN-NaK, CN-NaRb, CN-NaCs, CN-KRb, CN-RbCs, and CN-LiK, etc, were prepared following an identical procedure to that described above. The amounts of the added metal chlorides were equivalent to those used for KCl/CsCl. The samples were subjected to thermal treatment at 550 °C for 4 h under an argon atmosphere with a heating rate of 7 °C/min.

For comparative studies, a series of additional CN samples modified with binary alkali metals, including CN-NaK, CN-NaRb, CN-NaCs, CN-KRb, CN-RbCs, CN-LiK and CN-LiCs, etc, were prepared following an identical procedure to that described above. The amounts of the added metal chlorides were equivalent to those used for KCl/CsCl. The samples were subjected to thermal treatment at 550 °C for 4 h under an argon atmosphere with a heating rate of 7 °C/min.

Synthesis of CN-K and CN-Cs: To facilitate a comparative study, 2 g of the synthesized CN material was subjected to identical preparation procedures involving dispersion in 30 mL of deionized water followed by the addition of either 0.8 g KCl or 0.8 g RbCl. The thermal treatment was carried out at 550 °C for 4 h under an Ar atmosphere with a heating rate of 7 °C/min. The final products, denoted as CN-K and CN-Cs, were obtained through this synthetic route.

### Photocatalytic H_2_O_2_ production reaction

Photocatalytic O_2_ reduction to H_2_O_2_ coupled was investigated in a 60 mL open-top flat quartz reactor. A PCX-50C Discover Multi-channel photochemical reaction system equipped with a 10 W white LED light panel (Beijing PerfectLight) was employed as the illumination source. 15 mg of the synthesized photocatalyst was dispersed in 20 mL of a 0.5% (v/v) EG aqueous solution and sonicated for 10 min to ensure uniform dispersion. The reactor was illuminated from below with a LED lamp at an intensity of 100 mW/cm^2^, driving the photocatalytic H_2_O_2_ synthesis coupled with EG oxidation reaction. The irradiation intensity was measured using a PL-MW2000 high-intensity optical power meter. A magnetic stirrer maintained a constant rotational speed of 250 rpm, and the reaction temperature was maintained at 25 °C. The H_2_O_2_ production activity of each sample was determined independently in three times. During illumination, the reaction vessel was left open to the atmosphere, eliminating the need for external O_2_ bubbling.

The concentration of H_2_O_2_ products was quantified using the N,N-diethyl-p-phenylenediamine (DPD) method, which involves the oxidation of DPD by H_2_O_2_ catalyzed by horseradish peroxidase (HRP). The characteristic absorption peak of the oxidized DPD was measured at 551 nm using a Hitachi U-4100 spectrophotometer in the range of 400–650 nm. A standard curve was established by preparing solutions containing known concentrations of H_2_O_2_, followed by the addition of DPD and HRP. Specifically, To construct a calibration curve, 1.00 mL aliquots of H_2_O_2_ solutions was sampled from 20 mL mixture with varying concentrations, and then diluted with 1.20 mL of deionized water and 0.80 mL of phosphate buffer. Subsequently, 50 μL of DPD solution (100 mg DPD dissolved in 100 mL of 0.1 M H_2_SO_4_) and 50 μL of 1 g/L HRP aqueous solution were added to each mixture. For analysis, 0.1 mL of the collected reaction supernatant, after being filtered through a Millipore filter (0.22 μm), was added to 2.1 mL of water and 0.8 mL of phosphate buffer solution. Then, 50 μL of DPD and 50 μL of HRP aqueous solution were added, followed by rapid mixing and immediate measurement using the UV-vis spectrophotometer. Note that compared to the standard curve, adding 0.1 mL of the reaction supernatant corresponds to a 10-fold dilution.

Additionally, the oxidation products of EG within the reaction mixture were analyzed via high-performance liquid chromatography (HPLC) using a Fuli Instruments LC5090 system equipped with a UV detector operating at a wavelength of 210 nm. A Bio-Rad Aminex HPX-87H column (300 mm × 7.8 mm × 9 μm) was employed, and the mobile phase consisted of 5 mM H_2_SO_4_ at a flow rate of 0.6 mL/min. The detector temperature was maintained at 55 °C, and the total analysis time was 30 min. Product concentrations were determined by referencing a calibration curve constructed using external standards.

For stability tests, catalyst stability was evaluated by adding 15 mg of the catalyst to 20 mL of a 0.5% (v/v) aqueous EG solution. Following 2 h of irradiation, a 200 μL of liquid was taken for H_2_O_2_ analysis. The catalyst was then recovered by filtration, washed with water, and dried at 100 °C. It is important to avoid using ethanol for washing as it may leave residues. The regenerated catalyst was subsequently used in the same reaction system for the next 2 h of illumination for ten additional cycles.

### Calculation of quantum efficiency (QE) and solar-to-chemical conversion efficiency (SCC)

The wavelength-dependent quantum efficiency (QE) for H_2_O_2_ production was evaluated using a PCX-50C Discover multi-channel photochemical reaction system. Monochromatic illumination was provided at discrete wavelengths of 385, 420, 450, 485, 535, 595, and 630 nm (each with a ± 2 nm bandwidth). After 1 h of continuous irradiation at a stabilized temperature of 25 °C, the yield of H_2_O_2_ was quantified. The QE values at these specific excitation wavelengths were subsequently derived using the following equation:

10 $${{\rm{QE}}}\left(\%\right)=\frac{{{{\rm{N}}}}_{{{\rm{e}}}}}{{{{\rm{N}}}}_{{{\rm{P}}}}}=\frac{{{{\rm{number}}}} \, {{{\rm{of}}}} \, {{{\rm{reacted}}}} \, {{{\rm{electrons}}}}}{{{{\rm{number}}}} \, {{{\rm{of}}}}\, {{{\rm{incident}}}} \, {{{\rm{photons}}}}}\times 100\% $$

Note that herein, it is estimated that approximately 42–55% of the H_2_O_2_ is generated via the*O + *H_2_O pathway (a single-electron transfer process), while the rest is produced through the conventional 2e^–^ proton-coupled electron transfer (PCET) pathway. The photon number entered into the flat quartz reactor was determined by a light power meter (PL-MW2000).

The solar-to-chemical conversion (SCC) efficiency was evaluated under simulated solar illumination (100 mW/cm^2^) provided by a 300 W Xe lamp (Microsolar 300) with an AM 1.5 G filter. In a typical run, 15 mg of the photocatalyst was suspended in 20 mL of an aqueous solution containing 0.5% (v/v) ethylene glycol (EG). After 1 h of continuous light exposure, the SCC efficiency was determined using the following formula (11):

11 $${{{\rm{SCC}}}}=\frac{{\Delta {{{\rm{G}}}}}_{{{{{\rm{H}}}}}_{2}{{{{\rm{O}}}}}_{2}}\times {{{{\rm{N}}}}}_{{{{{\rm{H}}}}}_{2}{{{{\rm{O}}}}}_{2}}}{{{{\rm{I}}}}\times {{{\rm{S}}}}\times {{{\rm{T}}}}}\times 100\% $$

Where ΔG_H2O2_ represents the Gibbs free energy change associated with the formation of H_2_O_2_ (117 kJ/mol), N_H2O2_ is the moles of produced H_2_O_2_, I signifys the light intensity of simulated sunlight (100 mW cm^−2^), S represents the illuminated surface area (cm^2^), and T means the irradiation time (3600 s).

### Isotope labeling experiments

Isotope labeling experiments were performed in a sealed, flat quartz reactor under identical conditions to the photocatalytic evaluation experiments, with the exception of substituting ^18^O_2_ for O_2_ or H_2_^18^O for H_2_O as the reactant. After 2 h of irradiation, the reaction supernatant was collected by filtration through a 0.22 µm Millipore filter and transferred to a separate, sealed flat quartz reactor. To ensure a completely inert atmosphere, the reactor was subjected to multiple cycles of vacuum evacuation and Ar purging using an AC1000 Atmosphere controller. Benzoic acid (0.1 mM) was then added as a hydroxyl radical probe, followed by the introduction of Fe^2+^ ions (0.2 mM) to initiate a Fenton-like reaction, generating hydroxyl radicals (·OH) from H_2_O_2_. The mixture was stirred at room temperature for 60 min to ensure sufficient conversion of BA into hydroxylated products, primarily p-hydroxybenzoic acid (p-HBA). After the reaction, the mixture was extracted with ethyl acetate. The organic phase was collected, and concentrated under reduced pressure. The resulting products were analyzed by gas chromatography-mass spectrometry (GC-MS). The isotopic composition of oxygen incorporated into p-HBA was determined by monitoring the molecular ion peaks:m/z = 138 corresponding to ^16^O-containing p-HBA; m/z = 140 corresponding to ^18^O-labeled p-HBA. A Thermo Scientific Trace GC Ultra with a DSQ II GC/MS system was employed for the analysis. Helium was used as the carrier gas at a flow rate of 1.0 mL/min. The injector, electron ionization (EI) source, and GC column oven were maintained at temperatures of 200 °C, 230 °C, and 200 °C, respectively.

### Scalable continuous photocatalytic performance evaluation

To assess the scalable continuous photocatalytic performance of CN-KCs, a fixed-bed reactor was constructed using three interconnected cylindrical acrylic columns (19 mm inner diameter, 25 mm outer diameter, 300 mm height). The reactor was filled with CN-KCs beads to a volumetric filling ratio of 55%, and a 0.5% (v/v) aqueous EG)solution was continuously fed into the reactor at a flow rate of 0.6 L/h using a peristaltic pump. The concentration of produced H_2_O_2_ was monitored periodically, the hydraulic retention time (HRT) was extended to 72 h and using the white LED as the light source.

To immobilize the CN-KCs catalyst, a calcium alginate gel was employed. 0.5 g of of CN-KCs was added to 250 mL of a 3% (w/v) sodium alginate solution prepared using deionized water. The mixture was sonicated for 10 min to ensure uniform dispersion. The resulting suspension was then extruded dropwise into a cold 3% (w/v) CaCl₂ solution using a peristaltic pump. The CN-KCs beads were allowed to solidify at 4 °C for 2 h, washed with deionized water, and stored at 4 °C for subsequent experiments.

### General information for characterization

High-resolution synchrotron X-ray diffraction and total scattering assessments We performed scanning electron microscopy (SEM) to observe the micro-morphology by using a Field-Emission Hitachi SU8220 electron microscope. For transmission electron microscopy (TEM) imaging and STEM-EDX elemental mapping, we utilized a JEM-2100 JEOL transmission electron microscope. Aberration-corrected high-angle annular dark-field scanning transmission electron microscopy (AC HAADF-STEM) imaging was conducted with a FEI Titan Themis Z electron microscope from Thermo Fisher Scientific, operating at 200 kV. X-ray diffraction (XRD) patterns of the samples were recorded by employing a D-POWER X-ray powder diffractometer (GKINST Co.,LTD.) with Cu Kα1 radiation, spanning a 2θ range of 5-60°. Fourier-transform infrared (FTIR) spectra were recorded on a Thermo Fisher Nicolet IS50 II spectrophotometer within the 4000–400 cm-1 wavenumber range. N_2_ adsorption-desorption isotherms were measured at 77 K utilizing a Micromeritics ASAP 2020 system, following a 10 hours degassing pretreatment at 150 °C. The specific surface area was calculated via the Brunauer-Emmett-Teller (BET) method within the P/P_0_ range of 0.05–0.99.

For surface chemical analysis, X-ray photoelectron spectroscopy (XPS) was conducted with a Thermo Fisher K-Alpha+ instrument. Samples were prepared by compacting the sample onto aluminum foil, and all binding energies were calibrated to the adventitious C 1 s peak (284.8 eV) based on an average of 15 scans^37^. Organic elemental analyses for carbon, nitrogen, and hydrogen were conducted using an Elementar Vario MICRO cube, while metallic element analyses for K and Cs were performed with an Agilent 720ES Series inductively-coupled plasma optical emission spectrometer (ICP-OES). Diffuse reflectance UV-Vis spectra (DRS) were recorded with a range of 300–800 nm using a Hitachi U-4100 spectrophotometer, with barium sulfate (BaSO_4_) serving as the reference material. The optical band gaps were determined by applying the Kubelka-Munk function to the diffuse spectra.

Flat-band potential data were derived from Mott-Schottky plots recorded on a CHI760e workstation in a standard three-electrode configuration. A catalyst-modified FTO glass, a platinum plate, and an Ag/AgCl (in 3.5 M KCl) served as the working, counter, and reference electrodes, respectively. The experiments utilized a 0.2 M Na_2_SO_4_ aqueous solution as the electrolyte, with an AC potential applied at frequencies of 3000, 4000, and 5000 Hz.

The number of electrons transferred during the ORR process was evaluated via an RRDE setup (DC-DSR ROTATOR, PHYCHEMI). The working electrode was fabricated by coating 20 μL of the photocatalyst ink onto a glassy carbon disk, followed by air-drying at room temperature. A platinum ring served as the complementary part of the working electrode, while Ag/AgCl and Pt foil were employed as the reference and counter electrodes, respectively. The entire assembly was immersed in a 0.1 M KOH aqueous solution. The n values were subsequently calculated based on the slopes obtained from Koutecky–Levich analysis:

12 $${j}^{-1}={j}_{{{\rm{k}}}}^{-1}+{{{\rm{B}}}}^{-1}{{{{\rm{\omega }}}}}^{-1/2}$$

13 $${{{\rm{B}}}}=0.2{{{\rm{n}}}}F{{{\rm{v}}} }^{-1/6}C{D}^{2/3}$$

where *j* represents the measured current, j_*k*_ is the kinetic current density, B stands the proportionality coefficient and ω is the electrode rotating speed (r.p.m.), respectively. *F* is the Faraday constant. ν denotes the kinetic viscosity of solution, C is the concentration and *D* is the diffusion coefficient, refrenced to a standard ferricyanide system.

### High-resolution synchrotron X-ray total scattering experiments

Synchrotron-based high-resolution X-ray diffraction and total scattering data were acquired at the European Synchrotron Radiation Facility (ESRF, beamline ID31)^40^. Powdered samples were carefully sealed in Kapton-windowed cylindrical slots (1 mm thick) housed within a high-capacity sample loading system designed for fast screening. Measurements were executed in transmission geometry at a fixed incident energy of 75.00 keV, corresponding to a wavelength (λ) of 0.16531 Å. To ensure maximum signal acquisition, the diffraction data were captured by a Pilatus CdTe 2 M area detector (1475 × 1679 pixels with a 172 × 172 μm^2^ pixel size) offset from the primary beam center.

The sample-to-detector distance for total scattering measurements was maintained at around 0.3 m, and background measurements for the empty windows were recorded and subsequently subtracted. Geometry calibration was performed using NIST SRM 660b (LaB6) with the pyFAI software, followed by image integration that included flat-field, geometry, solid-angle, and polarization corrections. The PDF (Pair Distribution Function) data derived from the experiment were processed utilizing PDFgetX3 software with a Qmax value of 20 Å^−1^ applied during the processing. To enhance data accuracy and mitigate termination effects alongside high-frequency contributions, the data were further refined using the Lorch correction function. The X-ray absorption near-edge structure (XANES) measurements of C and N K-edge on the catalysts have been performed through the BL20A beam line of synchrotron radiation in the Singapore Synchrotron Light Source.

### In situ Fourier-transform infrared spectroscopy (ATR-FTIR) experiments

A Thermo Fisher Nicolet IS50 II spectrometer, equipped with a mercury cadmium telluride (MCT) detector cooled by liquid nitrogen, was employed for Attenuated total reflectance Fourier-transform infrared spectroscopy (ATR-FTIR) spectral acquisition. The experimental setup was integrated with a photocatalytic in-situ photochemical infrared module (Linglu Instrument (Shanghai) Co., Ltd.). For the optical interface, a ZnSe internal reflection element (IRE) with a 60° face angle, was also sourced from the Linglu Instrument, was employed. The sample was prepared by depositing 50 μL of the catalyst suspension (5 mg/mL) onto the IRE surface to create a slurry. The catalyst-coated IRE was subsequently securely positioned in the Photochemical Infrared Accessory. A 5 mL aliquot of a 0.5% ethylene glycol (EG) aqueous solution was deposited onto a single-crystal ZnSe ATR element within the sample chamber. Prior to acquiring the FTIR spectrum, the system was subjected to a 20 min degassing procedure under a flow of Ar to remove residual atmospheric gases. Background acquisition involved three iterations of 256 scans each. Following this, the ATR-FTIR absorbance data were captured over the wavenumber range of 4000–700 cm^−1^ with a resolution of 4 cm^−1^ and 32 scans averaged per spectrum. The background spectrum was subtracted to yield the final spectrum. A reference IR spectrum was recorded prior to the introduction of O₂ into the sample chamber. Subsequently, the pure O_2_ gas was introduced at a flow rate of 30 mL/min, and the spectrum was collected after 15 min of O_2_ flowing (designated as the 0-minute point) and at 5-minute intervals under white LED light illumination (light intensity was 50 mW/cm^2^). Comparative ATR-FTIR analysis was performed for the series of catalysts (CN, CN-K, CN-Cs, and CN-KCs) using a 0.5% EG aqueous solution. A control experiment consisting of the pure 0.5% EG solution without any solid material was included to account for background signals. A multi-channel mass flow control system, comprising INHA MF 200D Series Mass Flow Controllers & Meters, was employed to meticulously regulate both the flow rate and gas composition. Prior to experimentation, each mass flow controller underwent a gravimetric calibration.

### Temperature-programmed desorption (TPD) experiments

Oxygen temperature-programmed desorption (O_2_-TPD) analysis^37^ was executed using a Vdsorb-91i Chemisorption Analyzer. Before analysis, 10 mg of samples were held in a 2 mm diameter cylindrical glass microreactor, resting on a fixed clay bed with a height of 0.1–0.2 cm. Prior to TPD measurements, samples were pretreated at 400 °C for 4 h under a 30 mL/min He stream to remove moisture and adsorbed air. After cooling to 40 °C, the fixed bed was exposed to high-purity O_2_ for approximately 1 h to reach adsorption saturation. Residual physisorbed gas was then flushed out with He flow (30 mL/min) at the same temperature. Finally, the O_2_-TPD profiles were recorded by ramping the temperature to 400 °C at a rate of 20 °C/min. CO_2_-TPD analysis was performed using an identical protocol with CO_2_ as the probe gas.

For temperature-programmed desorption measurements involving liquid water (H_2_O), the pretreatment process mirrored that of O_2_-TPD to eliminate surface contaminants. The target H_2_O molecule was subsequently injected through the fixed clay bed at 120 °C. To ensure complete surface saturation, H_2_O was introduced in a pulse-counting mode, comprising 20 sequential doses with a 120 s interval for each. After flushing out physisorbed water molecules with a He stream, the H_2_O-TPD profiles were recorded by applying a linear temperature ramp of 10 °C/min up to a final temperature of 400 °C.

### In situ XPS experiments

In situ XPS measurements were conducted on a Thermo Fisher Escalab 250Xi X-ray photoelectron spectrometer to monitor the chemical evolution of CN-KCs under various conditions. A monochromatic Al Kα1 X-ray source with a spot size of approximately 500 μm and a fixed pass energy of 80 eV was employed. The catalyst sample, mounted on a silicon wafer, was introduced into analysis chamber. After vacummized to the ultra high vacuum level (UHV, i.e., up to 10^−10^ mbar), the sample was subjected to XPS analysis. Subsequent XPS spectra were acquired after 15 min of irradiation with a 300 W Xe lamp (Beijing PerfectLight Microsolar300). To investigate the effects of oxygen and water adsorption, the sample was exposed to 20 ml/min of pure O_2_ for 20 min to achieve adsorption equilibrium, followed by XPS analysis. The third condition, 0.5 mbar of H_2_O vapor was introduced at a flow rate of 20 ml/min for 20 min, and XPS spectra were collected after 15 min of irradiation. Finally, the sample was exposed to both O_2_ and H_2_O vapor simultaneously under similar conditions, and XPS spectra were acquired.

### Femtosecond transient absorption spectroscopy experiments

Femtosecond transient absorption spectroscopy was performed using a commercial Helios system (Ultrafast Systems LLC) with a time resolution of 190 fs and a noise level of approximately 0.1 mOD^41^. The excitation source was derived from a fundamental laser system (Coherent, 1030 nm center wavelength, 150 fs pulse duration, 8.9 kHz repetition rate). This beam was split, with the 9 W portion used to pump an optical parametric amplifier (TOPAS-PRIME, Light Conversion) and a mode-locked Ti:sapphire laser (Micra 5), generating tunable pump pulses spanning 317–2700 nm. Broadband probe pulses were generated by focusing 800 nm light onto a sapphire plate. The pump and probe beams were spatially overlapped on the sample using an aluminum parabolic reflector. A motorized optical delay line controlled the temporal delay between pump and probe pulses (0–8 ns). The transmitted probe pulse was then directed through a mechanical chopper (500 Hz) and collected. The sample, a suspension in a 2 mm quartz cuvette, was continuously stirred with a Teflon stir bar. To minimize pump scatter during the TA measurements, the probe light was filtered with a long-pass colored glass filter and spatially filtered with an iris before detection.

### Density functional theory (DFT) calculations

All DFT calculations were conducted via the Materials Studio Dmol3 package. We employed the generalized gradient approximation (GGA) with the Perdew–Burke–Ernzerhof (PBE) functional to manage electron exchange-correlation effects during geometric optimization. For K and Cs elements, density functional effective core potentials (ECPs) were employed to replace core electrons, while N and C were treated as all-electron cases. The double numerical plus polarization (DNP) basis set was used for calculations. Convergence criteria were set to a total energy tolerance of 2.0 × 10⁻^5^ hartree, atomic forces of less than 0.004 hartree/Å, and atomic displacements of less than 5 × 10⁻^3^ Å. To enhance computational efficiency, a Fermi smearing of 0.005 hartree was applied to orbital occupancy. A vacuum slab of 15 Å was introduced to prevent periodic interactions between unit cells. The Gibbs free energy change (ΔE) was calculated using the following equation: ΔE = E_total_ - E_sur_ - E_mol_ +ΔE_ZPE_-TΔS.

The Gibbs free energies (G) were derived from the most stable adsorption configurations, with vibrational corrections (ΔE_ZPE_ and TΔS) accounted for via the harmonic approximation. Here, the electronic energies of the adsorption system (E_total_), the pristine surface (E_sur_), and the isolated molecule (E_mol_) were employed. The zero-point energy difference (ΔE_ZPE_) and entropy change (ΔS) were incorporated to construct the final free energy profiles.

## Supplementary information

Supplementary Information

Descriptions of Additional Supplementary Files

Supplementary Data 1

Supplementary Data 2

Transparent Peer Review file

## Source data

Source Data

## Data Availability

The authors declare that all data supporting the findings of this study are available within the paper, supplementary information files, supplementary data and the provided source data files. Source data are provided with this paper.
