# Supplementary material for: Oxygen-oxygen bond cleavage enables efficient photocatalytic H2O2 production via an *O2 dissociation pathway
Source: Nat Commun. 2026 Jun 5;17:7218. doi: 10.1038/s41467-026-73685-x (PMC13396225; doi:10.1038/s41467-026-73685-x)
Supplement: Supplementary file 1 — Supplementary Information [file 41467_2026_73685_MOESM1_ESM.pdf]

Supplementary Information

Oxygen-oxygen bond cleavage enables efficient photocatalytic H_2O_2 production via an *O_2 dissociation pathway

Qiong Liu[1, 2], Tianxiang Chen[3], Tsz Woon Benedict Lo[3], Fuxian Wang[4, 5]*, Gangfeng Ouyang[4]*

[1] College of Environment and Climate, Guangdong Provincial Key Laboratory of Environmental Pollution and Health, Jinan University, Guangzhou 510632, China

[2] Institute of Analysis, Guangdong Academy of Sciences (China National Analytical Center, Guangzhou), Guangzhou, Guangdong, 510070, China

[3] Department of Applied Biology and Chemical Technology. The Hong Kong Polytechnic University, Hong Kong, China

[4] School of Chemical Engineering and Technology, Sun Yat-sen University, Zhuhai 519082, China

[5] Huaxin Chuangneng (Guangdong) Technology Co., Ltd., Foshan, 528000, China.

*E-mail: wangfx25@mail.sysu.edu.cn; cesoygf@mail.sysu.edu.cn

Table of Contents

Table of Contents 2

1. Supplementary Note 3

2. Figures and discussions 8

3. Tables 49

4. References 50

1. Supplementary Note

Supplementary Note 1, apparent quantum efficiency (AQE) calculation

It is estimated that approximately 42%–55% of the H_2O_2 is generated via the*O + *H_2O pathway (a single-electron transfer process), while the rest is produced through the conventional 2e⁻ proton-coupled electron transfer (PCET) pathway. For the revised calculation, we used a weighted average approach, assuming that 48.5% of the H_2O_2 yield follows the 1e⁻ pathway and 51.5% follows the 2e⁻ PCET pathway. The number of reacted electrons (N_e) is no longer simply $2\times N_{H2O2}$. Instead, it is calculated as follows: N_e=2×PCET yield of H_2O_2 (51.5%) +1×single electron of H_2O_2 (48.5%).

To calculate the AQE at the wavelength of 420±2 nm on using CN-KCs, the calculated process as follows:

$$N_e\text{=number of reacted electrons (mols}^{-1}\text{)}$$

$$=2\times\frac{n2}{t}+1\times\frac{n1}{t}=\frac{(2\times372.83+1\times351.12)\times10^{-6}}{3600}=3.047\times10^{-7}\ \text{mols}^{-1}$$

The number of H_2O_2 molecules from the production rate of H_2O_2 under 420 nm was calculated to be 723.95 μmol h^{-1}. Accordingly, the H_2O_2 generated via the single-electron reduction pathway is 351.12 μmol h^{-1} (723.95×48.5%), while that produced through the two-electron proton-coupled electron transfer (PCET) process is 372.83 μmol h^{-1}. Then, the received reacted electron number of the H_2O_2 products (N_e) is measured as $(2\times372.83+1\times351.12)\times10^{-6}/3600=3.047\times10^{-8}$ $mols^{-1}$.

The energy of a single photon (E_s) at λ = 420 nm is calculated according to hc/λ, where h and c are the Planck constant 6.63×10^{-34} J s and the speed of light 3×10^{8} m/s, respectively, E_s was found to be 4.736×10^{-19} J.

$$E_S=\frac{hc}{\lambda}=\frac{6.63\times10^{-34}\times3\times10^{8}}{4.2\times10^{-7}}=4.736\times10^{-19}\ \text{J}$$

The Avogadro's number (denoted as A) representing the number in one mole of a substance is 6.022 × 10^{23}. The incident light intensity (I) was measured as 12.5 mW/cm^2, calibrated by a light power meter (PL-MW2000). The reactor's illumination window, approximately 3.5 cm in diameter, yielded

an irradiation area (S) of 9.616 cm^2. Consequently, the total number of photons incident (Np) offered in the reaction vessel per hour was calculated to be 4.214×10^{-7} mol s^{-1}.

$$N_p=\frac{I\times S}{E_s\times A}=\frac{12.5\times10^{1}\times9.616\times10^{-4}}{4.736\times10^{-19}\times6.022\times10^{23}}=4.214\times10^{-7}$$

Thus, according to equation (1), the AQE (%) =72.3%.

$$AQE(\%)=\frac{N_e}{N_P}=\frac{\text{number of reacted electrons}}{\text{number of incident photons}}\times100\%$$

$$=\frac{3.047\times10^{-7}}{4.214\times10^{-7}}\times100\%=72.3\%$$

Take AQY at the wavelength of 385±2 nm of CN-KCs as an other example,

The number of H_2O_2 744.8molecules from the production rate of H_2O_2 under 385 nm was calculated to be 352.32 μmol h^{-1}. Accordingly, the H_2O_2 generated via the single-electron reduction pathway is 170.88 μmol h^{-1} (352.32×48.5%), while that produced through the two-electron proton-coupled electron transfer (PCET) process is 181.44 μmol h^{-1}. Consequently, the total number of reacted electrons for the H_2O_2 products (N_e) is determined to be (2×181.44+1×170.88)×10^{-6}/3600=1.483×10^{-7} mols^{-1}.

$$N_e=\text{number of reacted electrons (mol}s^{-1})$$

$$=2\times\frac{n2}{t}+1\times\frac{n1}{t}=\frac{(2\times181.44+1\times170.88)\times10^{-6}}{3600}=1.483\times10^{-7}\ mols^{-1}$$

The energy of a single photon (E_s) at λ = 385 nm is calculated to be 5.234×10^{-19} J, the light intensity (I) was measured as 6.5 mW/cm^2. Consequently, the total number of photons incident (Np) offered in the reaction vessel per hour was calculated to be 1.983×10^{-7} mol s^{-1}.

$$N_p=\frac{I\times S}{E_s\times A}=\frac{6.5\times10^{1}\times9.616\times10^{-4}}{5.234\times10^{-19}\times6.022\times10^{23}}=1.983\times10^{-7}$$

Thus, according to equation (1), the AQE (%) =74.8%.

$$AQE(\%)=\frac{N_e}{N_P}=\frac{\text{number of reacted electrons}}{\text{number of incident photons}}\times100\%$$

$$=\frac{1.483\times10^{-7}}{1.983\times10^{-7}}\times100\%=74.8\%$$

Supplementary Note 2, the solar-to-chemical conversion (SCC) efficiency calculation

The SCC efficiency on CN-KCs is calculated by the following equation :

$$SCC(\%)=\frac{\Delta G_{H_2O_2}\times N_{H_2O_2}}{I\times S\times T}\times 100\%$$

Where ΔG_{H2O2} represents the Gibbs free energy change associated with the formation of H_2O_2 (117 kJ/mol), N_{H2O2} stands the moles of produced H_2O_2 of which was calculated to be 1806.6 μmol h^{-1}. I signifys the light intensity of simulated sunlight (100 mW cm^{-2}), S represents the illuminated surface area irradiation area of 9.616 cm^2, and T means the irradiation time of 3600 s. As such, the calculated total input energy was 3461.76 J, the power generated by H_2O_2 formed was 211.37 J.

$$SCC(\%)=\frac{117\times 10^3\times 1806.6\times 10^{-6}}{100\times 10^1\times 9.616\times 10^{-4}\times 3600}\times 100\%$$

$$=\frac{211.37\ J/s}{3461.76\ W}\times 100\%=6.1\%$$

Supplementary Note 3, the *O + *H_2O pathway calculation

Based on a semi-quantitative estimation from the isotopic peak ratios, we infer that approximately 42%–55% of H_2O_2 is generated via the *O + *H_2O pathway, while the remaining fraction is produced through the conventional PCET route. The detailed calculation process is as follows:

To quantitatively determine the contribution of each pathway, we denote the molar fraction of H_2O_2 produced via the $2e^-$ PCET pathway as x and that via the $1e^-$ *O + *H_2O as y. The total H_2O_2 yield is normalized as x + y = 1.

In Experiment A (**$^{18}O_2 + H_2{}^{16}O$**), the isotopic composition of the generated H_2O_2 is as follows:

- $x\cdot H_2{}^{18}O_2$: Derived from the PCET pathway.
- $y\cdot H_2{}^{18}O^{16}O$: Derived from the *O+ *H_2O pathway, where one O atom originates from $^{18}O_2$ and the other from $H_2{}^{16}O$.

Upon the addition of Fe^{2+} for PHB detection, the O-O bond of H_2O_2 undergoes homolytic cleavage to generate·OH radicals. The distribution of the resulting·OH isotopes is:

1. From $H_2{}^{18}O_2$: Produces 2x moles of ^{18}OH.

2. From $H_2{}^{18}O^{16}O$: Produces y moles of ^{18}OH and y moles of ^{16}OH (equimolar distribution).

The intensity of the MS peaks for ^{18}O-PHB (m/z=140) and ^{16}O-PHB (m/z=138) is proportional to the concentration of their respective ·OH precursors:

- $I(m/z=140) \propto 2x + y$
- $I(m/z=138) \propto y$

Based on the experimental intensity ratio $I(138)/I(140) \approx 0.34$, we have the following equation:

$y/(2x + y) = 0.34$

Substituting this into $x + y = 1$:

$y \approx 0.507$ (50.7%)

$x \approx 0.493$ (49.3%)

This derivation demonstrates that 50.7% of the H_2O_2 incorporates one oxygen atom from the H_2O solvent via the $*O + *H_2O$ pathway."

"In Experiment B (O_2 + $H_2{}^{18}O$), experimental intensity ratio $I(140)/I(130) \approx 0.30$, Based on a similar calculation formula, it can be derived that the $*O + *H_2O$ pathway accounts for approximately 46.2%, while the PCET pathway accounts for approximately 53.8%.

- $I(m/z=138) \propto 2x + y$
- $I(m/z=140) \propto y$

Based on the experimental intensity ratio $I(140)/I(138) \approx 0.30$, we have the following equation:

$y/(2x + y) = 0.30$

Substituting this into $x + y = 1$:

$y \approx 0.462$ (46.2%)

$x \approx 0.538$ (53.8%)

This derivation demonstrates that 46.2% of the H_2O_2 incorporates one oxygen atom from the H_2O solvent via the $*O + *H_2O$ pathway.

Based on this improved analysis, the isotopic distribution is consistent with our proposed mechanism, indicating that approximately 42–55% of H_2O_2 originates from the *O+ *H_2O pathway, while the remaining 45–58% is produced via the conventional PCET pathway.

2. Figures and discussions

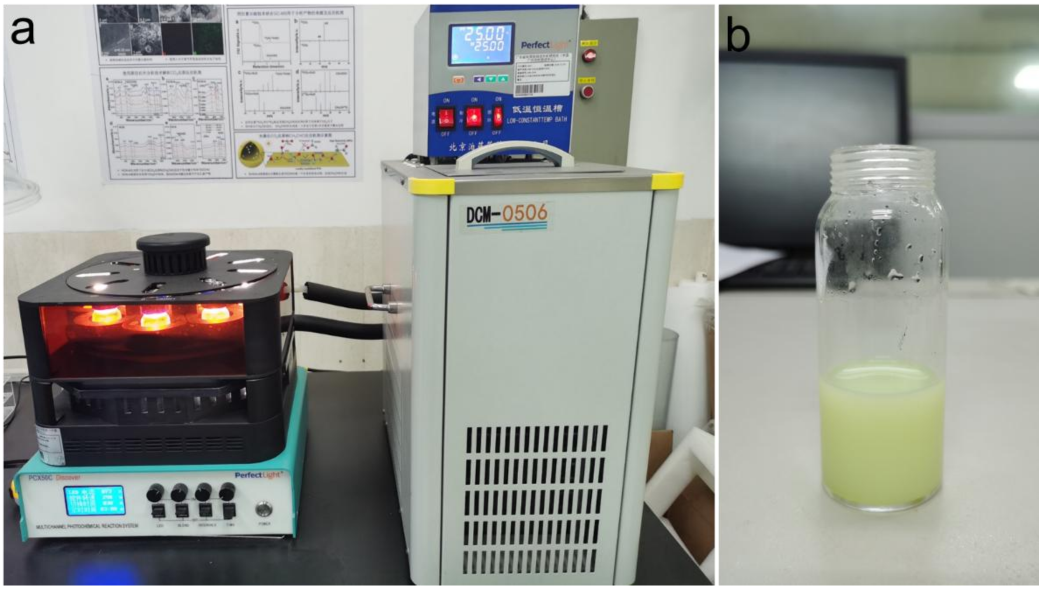

Supplementary Fig. 1. (a) Digital photograph showing the photocatalytic reaction apparatus and (b) reaction reactor used in this study.

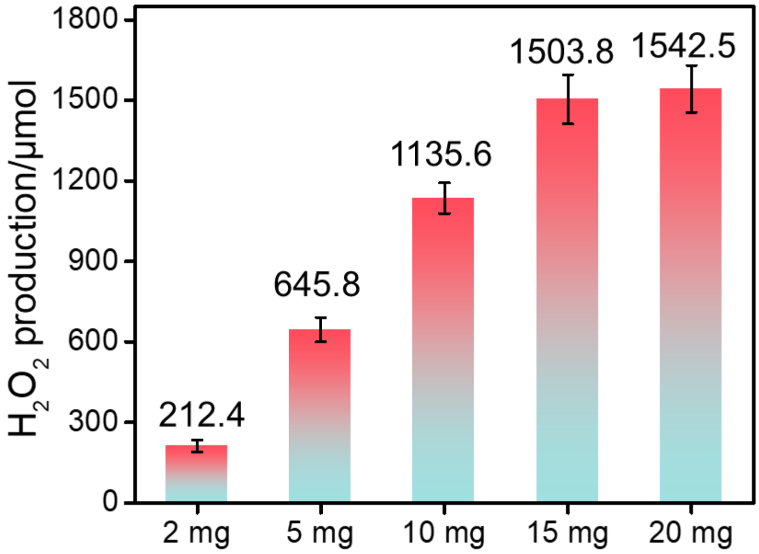

Supplementary Fig. 2. The average H_2O_2 generation rate at different catalyst mass using CN-KCs catalyst with air, 20 mL of a 0.5% (v/v) EG aqueous solution, 25 °C and illuminated by white LED lamp

at an intensity of 100 mW/cm². Error bars represent the standard deviation from three independent replicates.

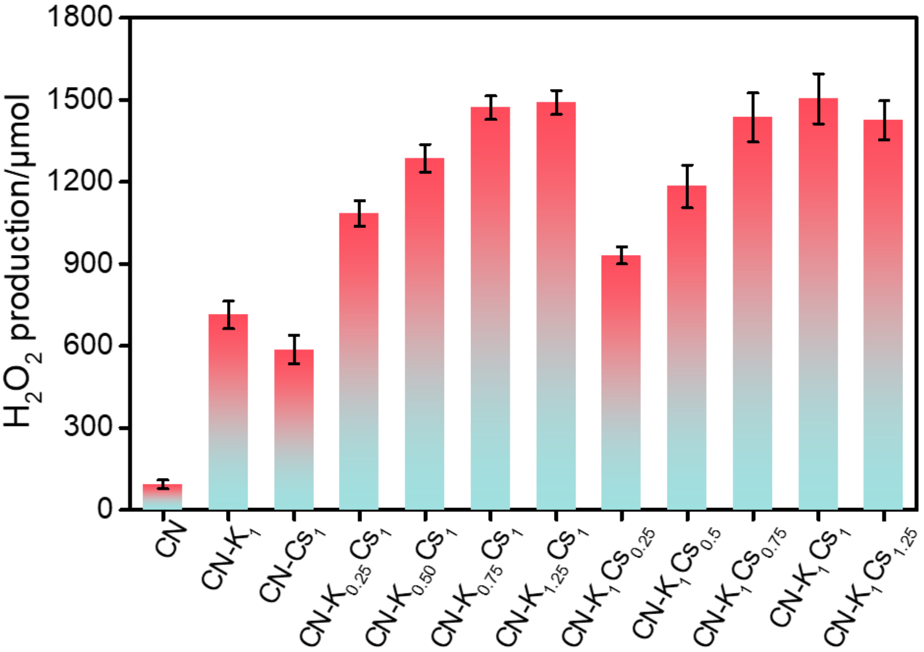

Supplementary Fig. 3. The average H_2O_2 generation rate at different different K and Cs loadings using CN-KCs catalyst with air, 20 mL of a 0.5% (v/v) EG aqueous solution, 25 °C and illuminated by white LED lamp at an intensity of 100 mW/cm^2. Error bars represent the standard deviation from three independent replicates.

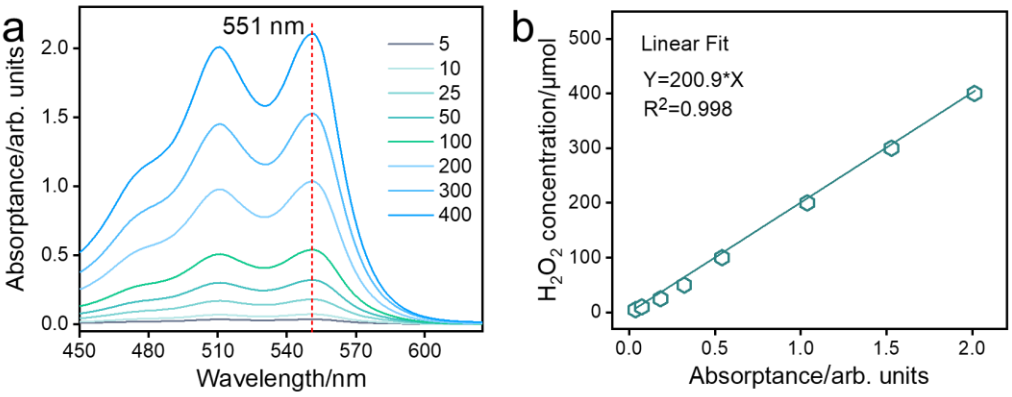

Supplementary Fig. 4. (a) UV-Vis spectra of H_2O_2 at various concentrations and (b) The corresponding calibration curve for H_2O_2 concentration determination using N,N-diethyl-p-phenylenediamine (DPD) method. Note that compared to the standard curve, adding 0.1 mL of the reaction supernatant corresponds to a 10-fold dilution. To construct a calibration curve, 1.00 mL aliquots of H_2O_2 solutions was sampled from 20 mL mixture with varying concentrations, and then diluted with 1.20 mL of deionized water and 0.80 mL of phosphate buffer.

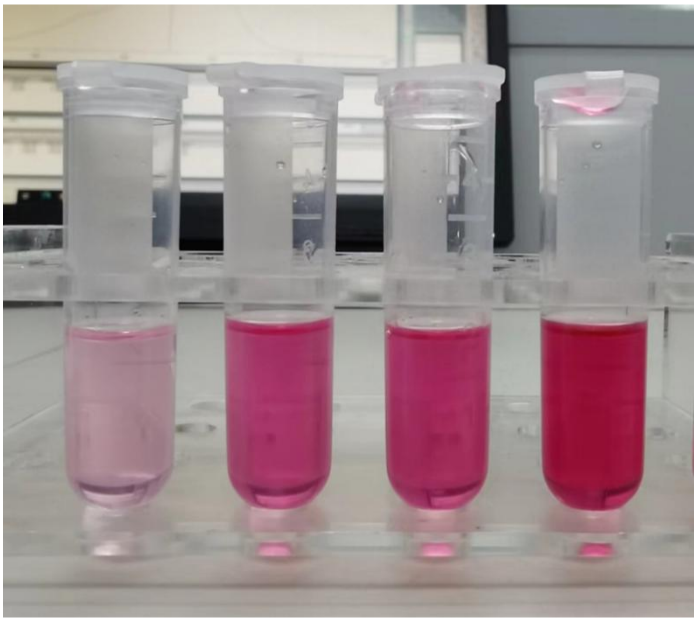

Supplementary Fig. 5. The digital picture of generated H_2O_2 concentration using N,N-diethyl-p-phenylenediamine (DPD) method after adding 50 µL DPD and 50 µL HPR, Note that compared to the standard curve, adding 0.1 mL of the reaction supernatant corresponds to a 10-fold dilution.

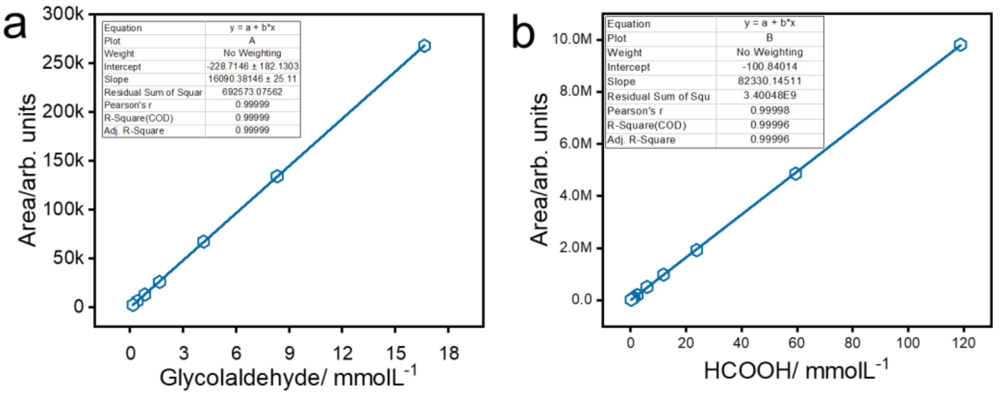

Supplementary Fig. 6. The standard fitting curve of (a) glycolaldehyde and (b) HCOOH concentration, detected by using high-performance liquid chromatography (HPLC).

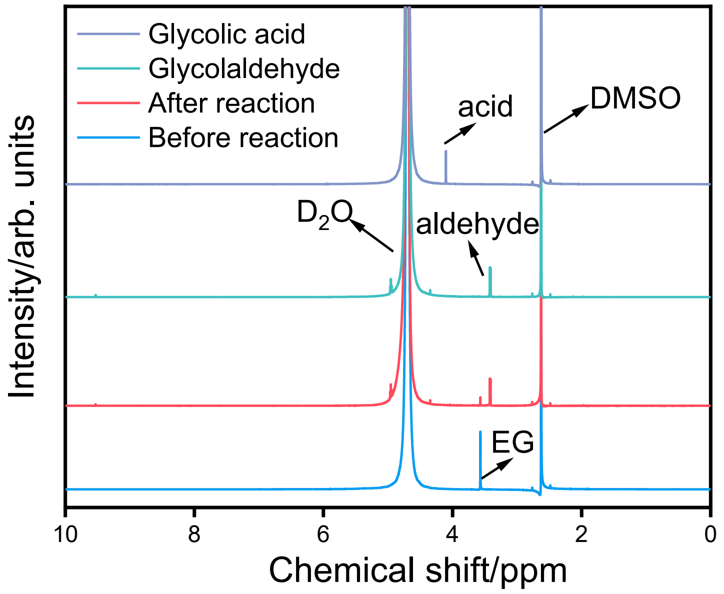

Supplementary Fig. 7. The NMR spectrum of the oxidative product from EG oxidation, for the NMR analysis, 0.7 ml target liquid sample, 50 µL D_2O and 10 ul of DMSO.

Concurrently, ethylene glycol (EG) was oxidized into formic acid, glycolaldehyde, and glycolic acid (Figure 1c and Supplementary Figs. 6–7). The CN-K shows a selectivity of 67.7% for glycolaldehyde, just slightly higher than CN, along with approximately 26.5% glycolic acid. Both CN-Cs and CN-KCs

exhibit over 90% selectivity for glycolaldehyde generation. CN-KCs achieves the highest yield for glycolaldehyde from EG with a high selectivity of 91.9%, consistent with its high ORR activity. It should be noted that selectively oxidizing of EG into glycolaldehyde can alleviate the sluggish kinetics compared with water oxidation, and enhancing the value of the oxidation half-reaction.

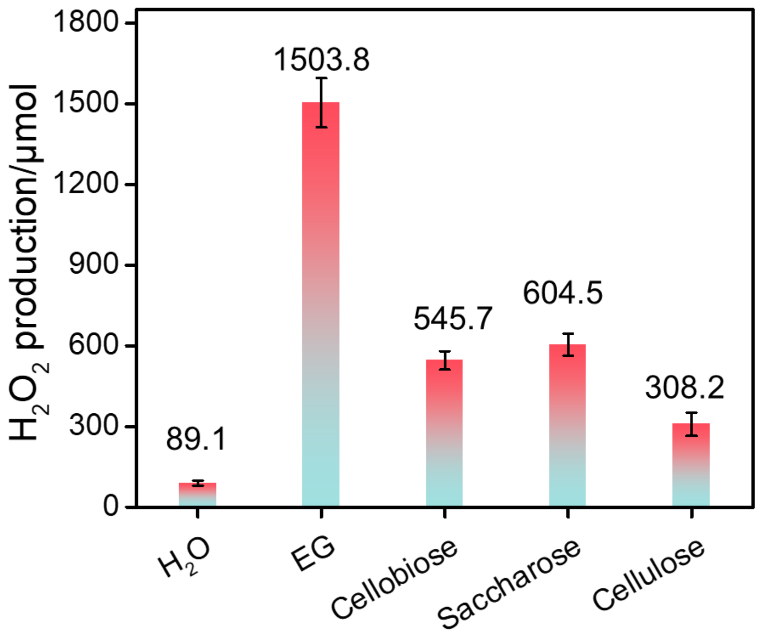

Supplementary Fig. 8. Photocatalytic H_2O_2 generation rates in different 0.5% (v/v) or 0.5% wt biomass derivatives aqueous solution using CN-KCs. Error bars represent the standard deviation from three independent replicates.

Moreover, when EG was omitted and only H_2O and air were present, the H_2O_2 yield dropped significantly to 89.1 μmol. It is indicating that while the reaction could still proceed, its rate was severely limited by the oxidation half-reaction. This result demonstrates the addition of EG accelerated this oxidation half-reaction, thereby facilitating the overall photocatalytic redox process. Replacing ethylene glycol (C_2) with other biomass-derived compounds such as glycerol (C3), erythritol (C4), xylitol (C5), and glucose (C6) (Fig. 1d) also resulted in excellent photocatalytic H_2O_2 yield, indicating the broad applicability of biomass substrates for accelerating the oxidation half-reaction. The trend in yield using the different catalysts was generally consistent with that of EG, with CN-KCs

still demonstrating the highest activity. We further applied the disaccharide of cellobiose, saccharose or the typical polysaccharide of cellulose as substrate (Supplementary Fig. 8), it is found the clearly increased H_2O_2 yield compared to the pure H_2O. This signifies that the substrate is not limited to EG. In this reaction system, biomass-derived polyols, monosaccharides, disaccharides, and cellulose can all serve as substrates to accelerate the kinetics of the oxidative half-reaction, thereby promoting the synthesis of H_2O_2. Replacing EG (C2) with other biomass-derived compounds also resulted in excellent photocatalytic H_2O_2 yield (Figure 1d and Supplementary Figs. 8-9), indicating the broad applicability of biomass substrates. The comparison shows that the significantly higher activity using EG, compared to the lower activity of commonly used alcohols such as MeOH or EtOH, confirms the advantages of EG (Supplementary Fig. 9).

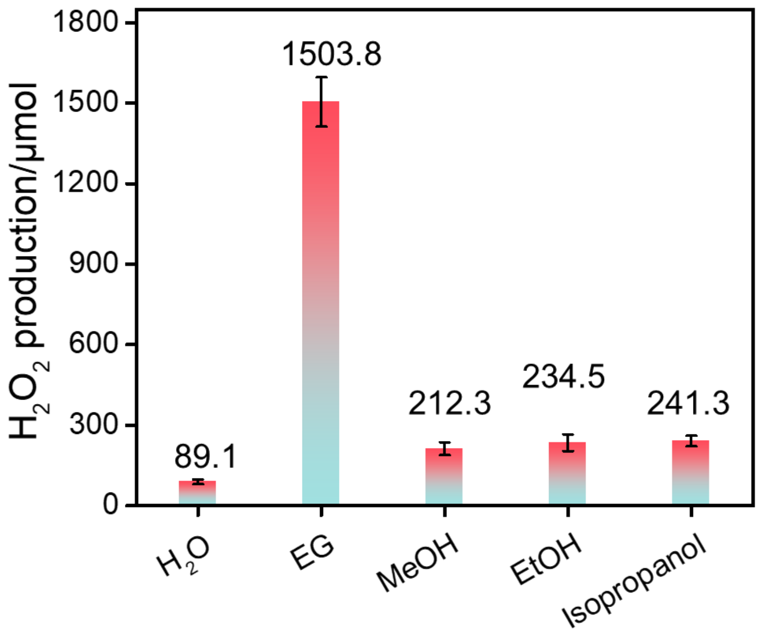

Supplementary Fig. 9. Photocatalytic H_2O_2 generation rates in different 0.5% (v/v) substrate aqueous solution using CN-KCs. As shown in the results, when using commonly used hole scavengers such as MeOH, EtOH, or isopropanol at the same concentration, their activity is significantly lower than that of EG. This highlights the advantages of the biomass-derived EG. Error bars represent the standard deviation from three independent replicates.

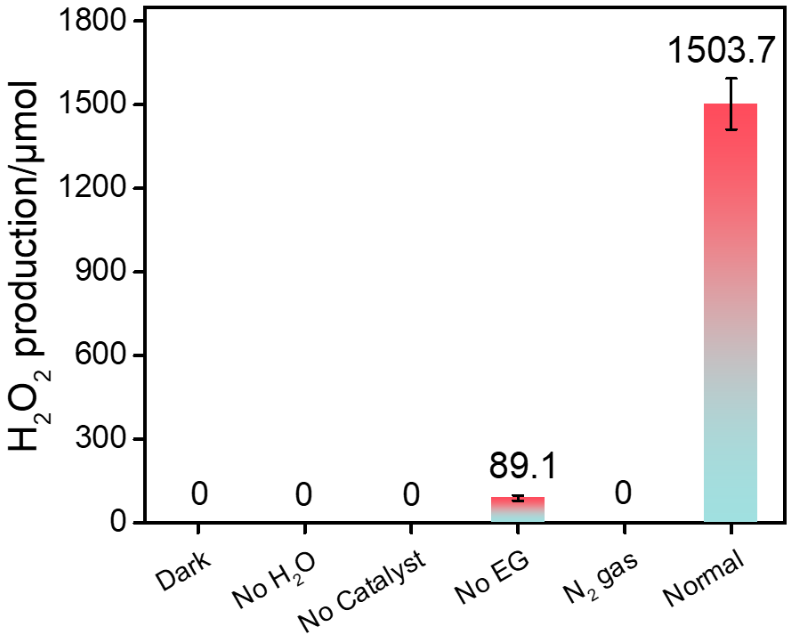

Supplementary Fig. 10. H_2O_2 generation rates in different control experiments by using CN-KCs. Error bars represent the standard deviation from three independent replicates.

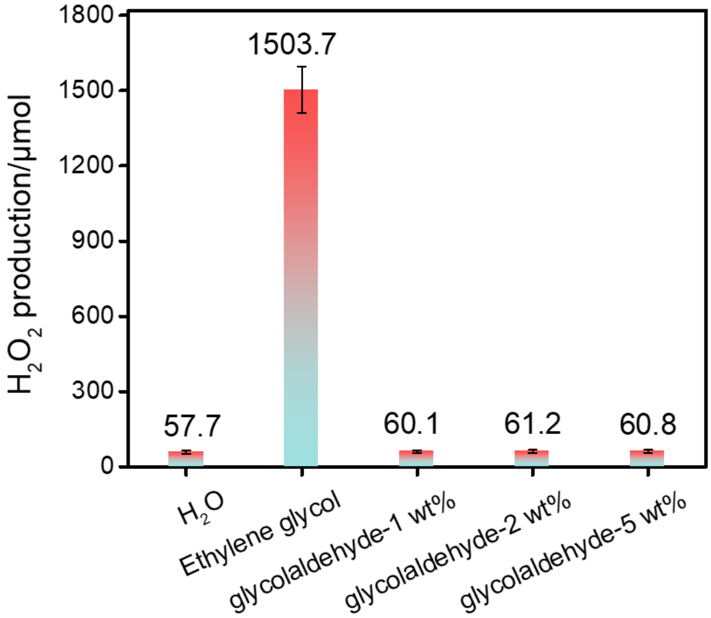

Supplementary Fig. 11. The H_2O_2 generation activity with varying amounts of glycolaldehyde compared with that using ethylene glycol as the substrate. This result clearly indicates that

glycolaldehyde does not contribute to H_2O_2 formation. Error bars represent the standard deviation from three independent replicates.

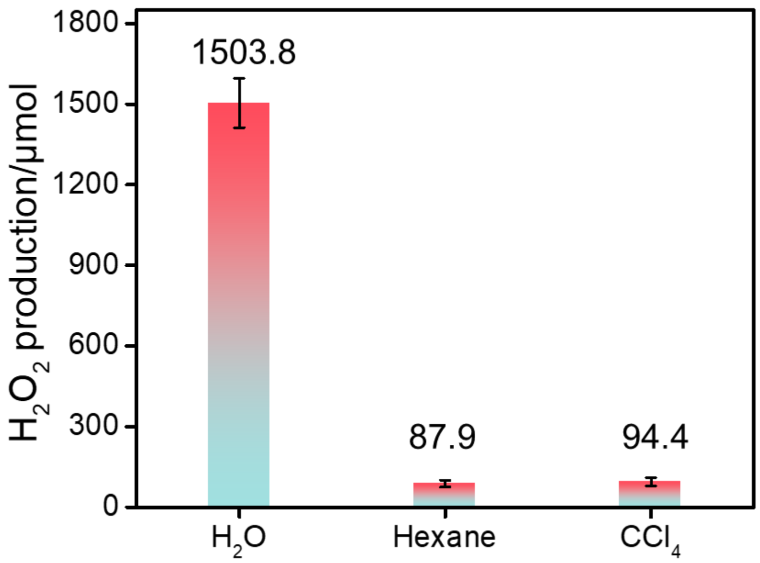

Supplementary Fig. 12. H_2O_2 generation rates in different solvent with a 0.5% (v/v) EG using CN-KCs. Error bars represent the standard deviation from three independent replicates.

When non-protic solvents were used instead of H_2O (Supplementary Fig. 12), the reaction was largely ineffective, highlighting the necessity of the H_2O involved process for H_2O_2 generation.

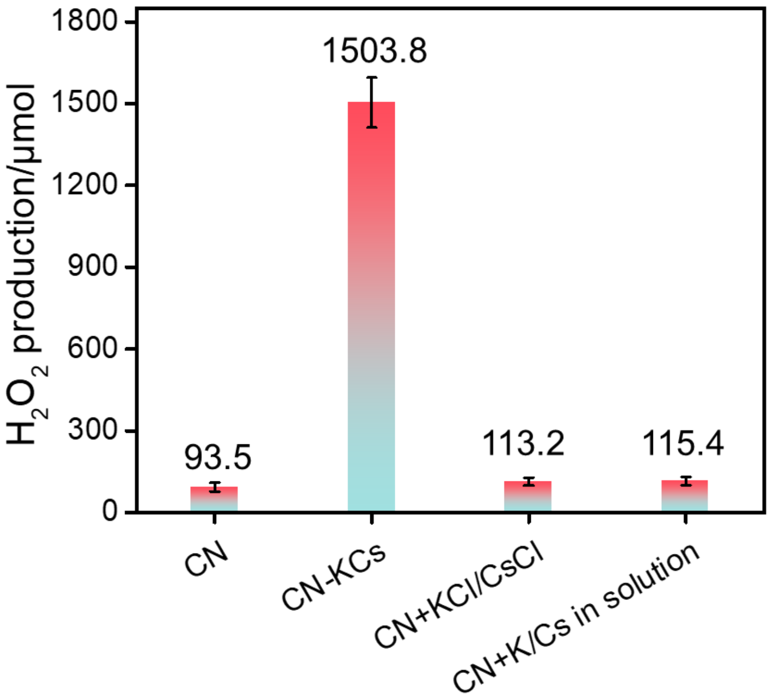

Supplementary Fig. 13. Photocatalytic H_2O_2 generation rates in different 0.5% (v/v) EG aqueous solution using different catalyst. The sample labeled CN+KCl/CsCl refers to the mixture of CN, KCl, and CsCl that has been ground together for 10 minutes. In contrast, CN+K/Cs in solution indicates that CN is used as the catalyst, with the addition of 0.2 wt% KCl/CsCl salt in the aqueous solution. The catalytic activity for H_2O_2 production is then compared after exposure to light. Error bars represent the standard deviation from three independent replicates.

Further control experiments reveal that neither physically mixing CN with KCl/CsCl nor adding a certain amount of KCl/CsCl to the reaction solution (Supplementary Fig. 13) improves H_2O_2 synthesis activity. This finding suggests that a high yield of H_2O_2 can only be achieved through the formation of the chemical bonding between CN and KCl/CsCl.

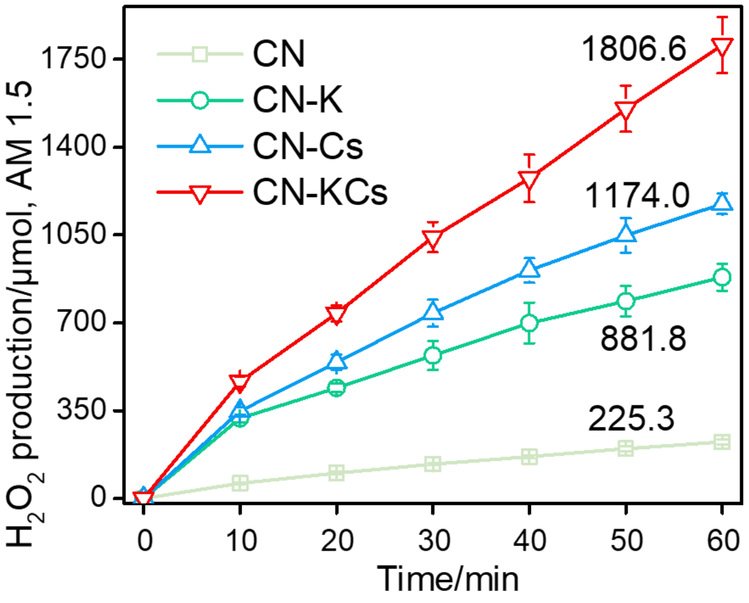

Supplementary Fig. 14. Time course of H_2O_2 generation on CN, CN-K, CN-Cs and CN-KCs by using AM 1.5 as the illuminated source at an intensity of 100 mW/cm². Error bars represent the standard deviation from three independent replicates.

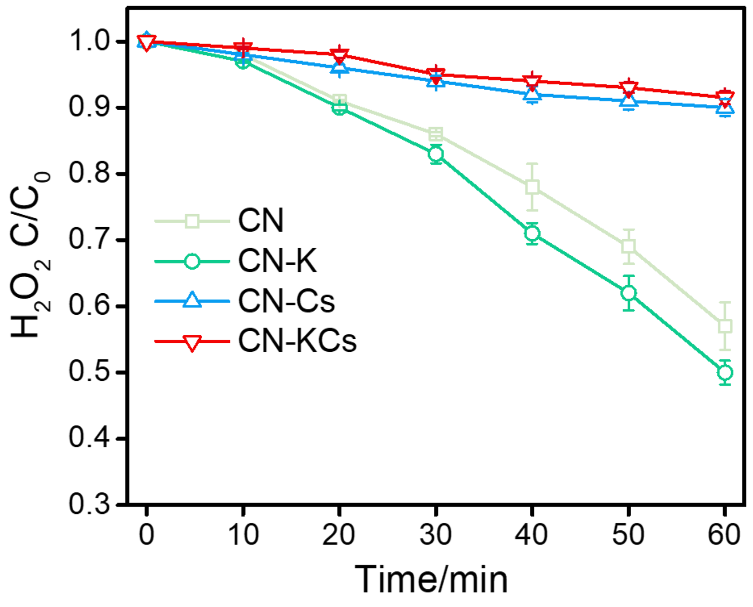

Supplementary Fig. 15. The degradation rate of H_2O_2 using the obtained photocatalysts with the initial 5 mmol concentration of H_2O_2. Error bars represent the standard deviation from three independent replicates.

To evaluate the photocatalytic performance in terms of H_2O_2 overoxidation, the catalysts were dispersed in a 5 mmol aqueous H_2O_2 solution (Supplementary Fig. 15). It was observed that both CN and CN-K decomposed more than 40% of the H_2O_2 within 1 hour, whereas the decomposition rates on CN-Cs and CN-KCs were less than 10%. This indicates that the CN-Cs and CN-KCs systems can effectively minimize the side reaction of H_2O_2 overoxidation and keep the stability of H_2O_2 generation during the process.

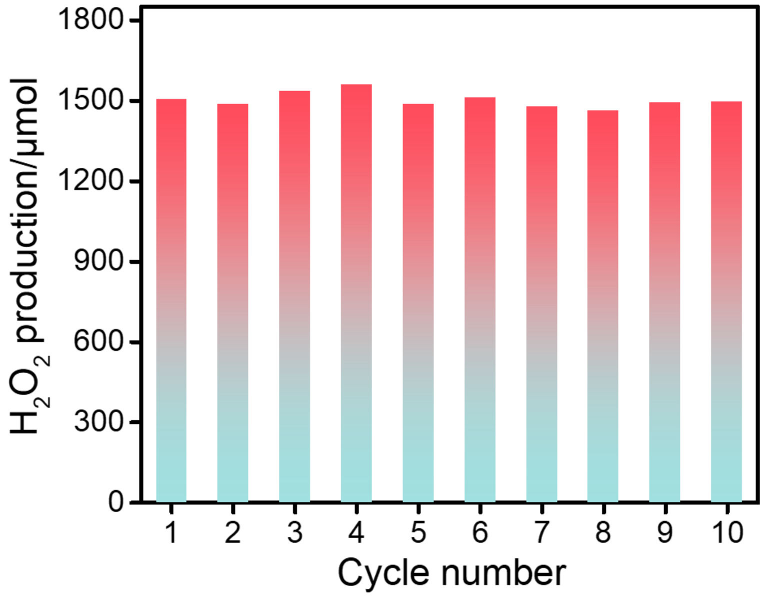

Supplementary Fig. 16. Photocatalytic H_2O_2 generation rates for the 10 cycles stability test using CN-KCs in different 0.5% (v/v) EG aqueous solution.

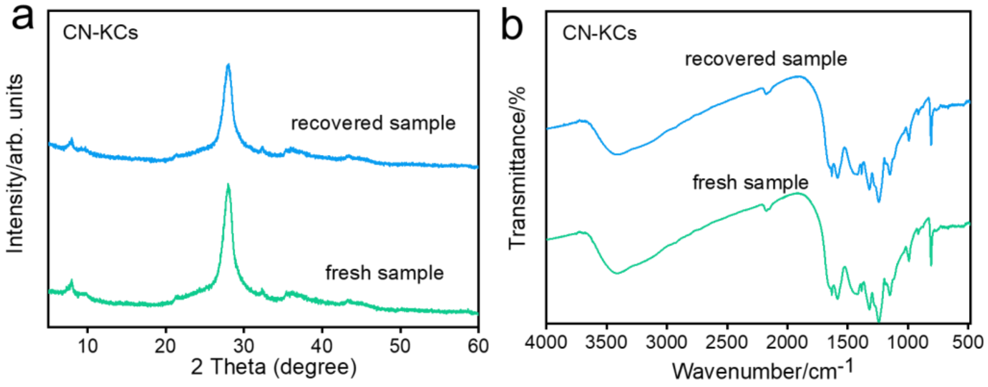

Supplementary Fig. 17. (a) XRD patterns and (b) FTIR spectrum of the fresh and tested CN-KCs.

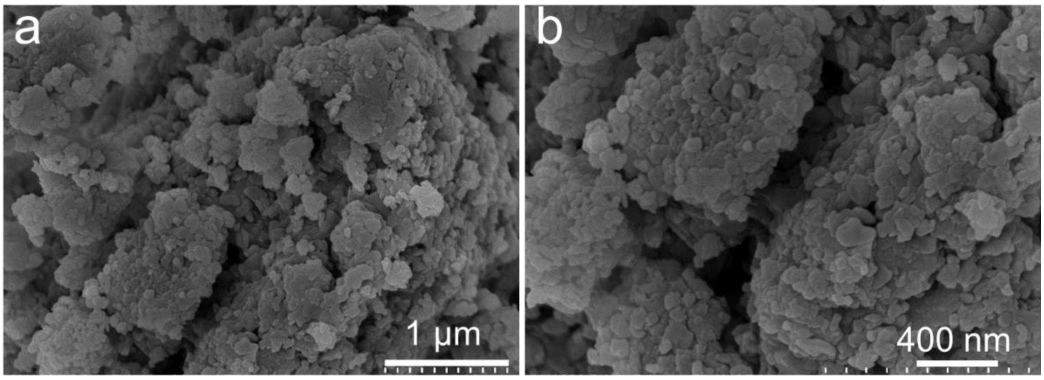

Supplementary Fig. 18. (a-b) SEM image of the tested CN-CuKCs.

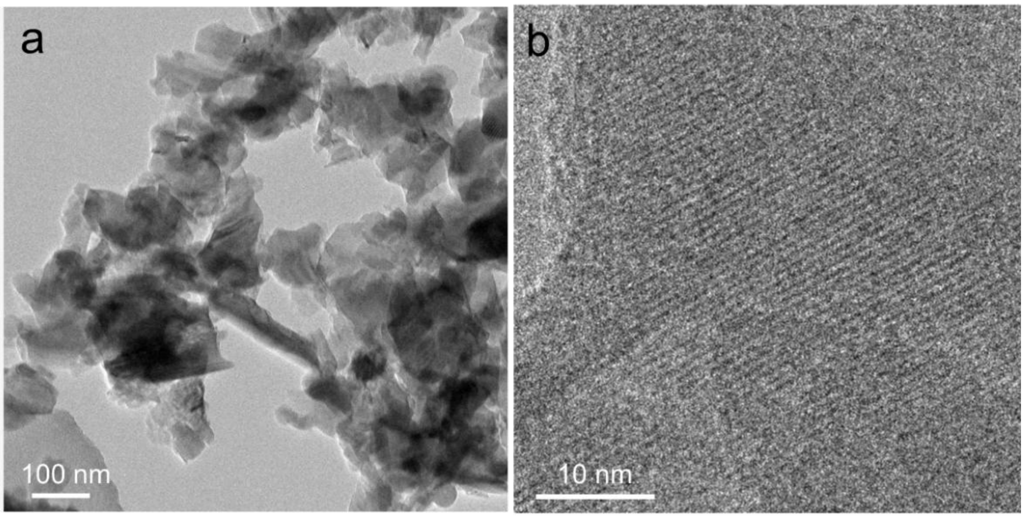

Supplementary Fig. 19. (a-b) TEM image of the tested CN-KCs.

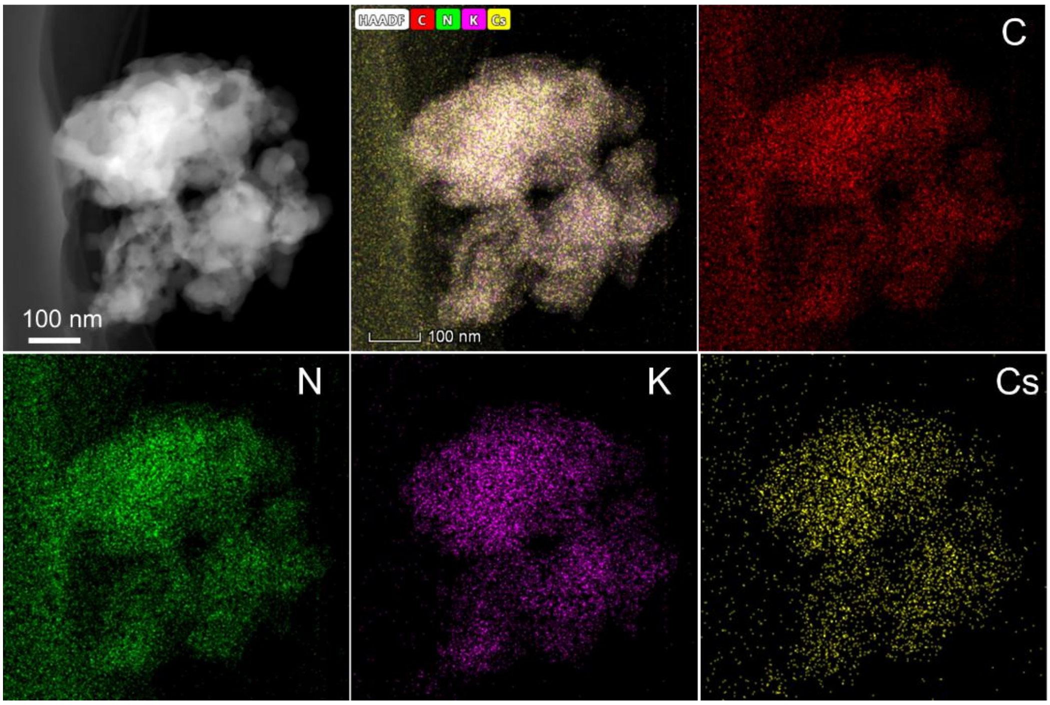

Supplementary Fig. 20. STEM elemental mapping images of C, N, K and Cs for the tested CN-KCs.

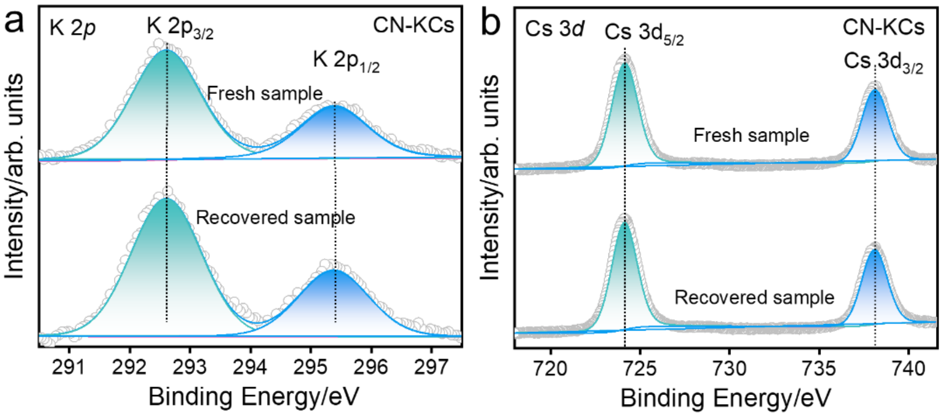

Supplementary Fig. 21. High-resolution XPS spectra of (a) K 2*p* peaks and (b) Cs 3*d* peaks for fresh and tested CN-KCs catalyst.

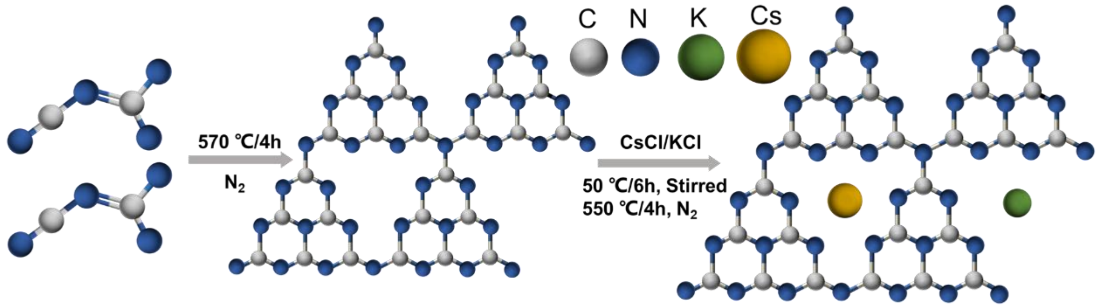

Supplementary Fig. 22. Schematic diagram of CN-KCs catalyst synthesis.

The synthetic pathway for introducing dual K and Cs atoms into CN consists of two primary steps. Initially, pristine carbon nitride is synthesized via the high-temperature thermal polymerization of dicyandiamide. The resulting carbon nitride (CN) is then combined with KCl and CsCl in water, followed by heating, stirring, and drying. Subsequently, high-temperature ionothermal treatment is applied to incorporate K and Cs into the CN matrix, leading to the formation of CN-KCs (schematic illustration for the synthesis of CN-KCs shown in Supplementary Fig. 22).

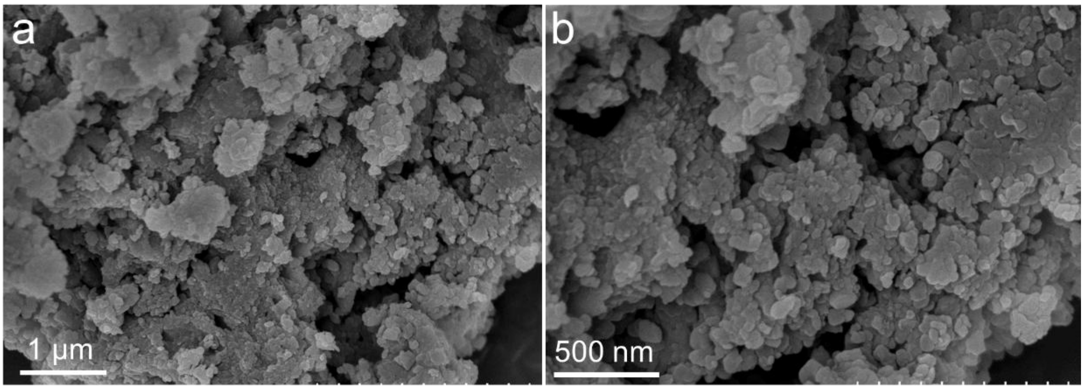

Supplementary Fig. 23. (a-b) SEM image of the CN-KCs.

The structure of CN-KCs is comprehensively characterized to elucidate the roles of K and Cs in enhancing performance. SEM image reveals the relatively uniform size of the 2D nanosheets of CN-KCs (Supplementary Fig. 23 and Fig. 2a), which is comparable to that of CN-Cs (Supplementary Fig. 24) but distinctly different from the irregularly stacked particles observed in CN and CN-K (Supplementary Fig. 25). TEM image of CN-KCs (Fig. 2b-2d)

corroborates the SEM findings, displaying nanosheet structures approximately 50-100 nm in size. Additionally, the selected area electron diffraction (SAED) pattern shows prominent diffraction rings and spots. Fig. 2d illustrates clear lattice fringes with a d-spacing of 0.78 nm, indicating a significant increase in crystallinity for CN-KCs compared to the typically synthesized amorphous CN. TEM images and SAED patterns for CN-K (Supplementary Fig. 27) and CN-Cs (Supplementary Fig. 28) reveal that CN-K exhibits bright diffraction rings and enhanced crystallinity, similar to CN-KCs, while CN-Cs shows slightly lower crystallinity than CN-K. This suggests that the incorporation of the alkali metal K/Cs improves the crystallinity of the CN. Crystal lattice or aggregates of K/Cs components is not observed in the high-resolution TEM (HR-TEM) image of CN-KCs.

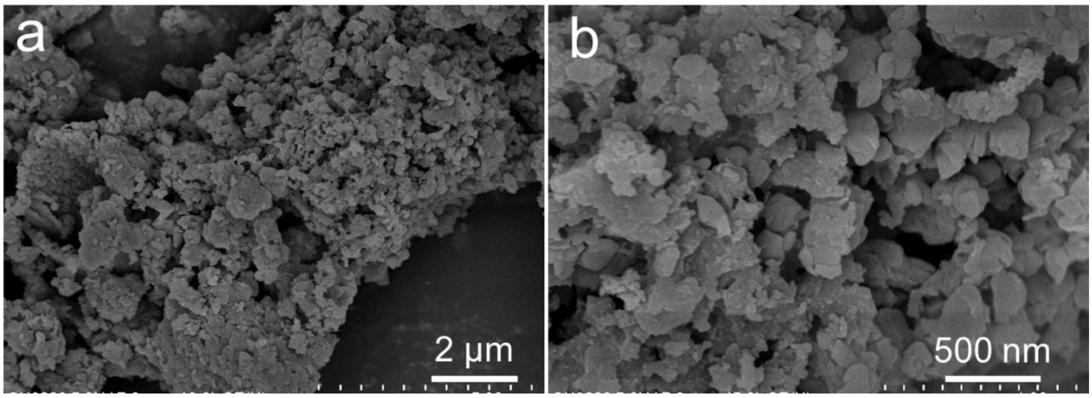

Supplementary Fig. 24. (a-b) SEM image of the CN-Cs.

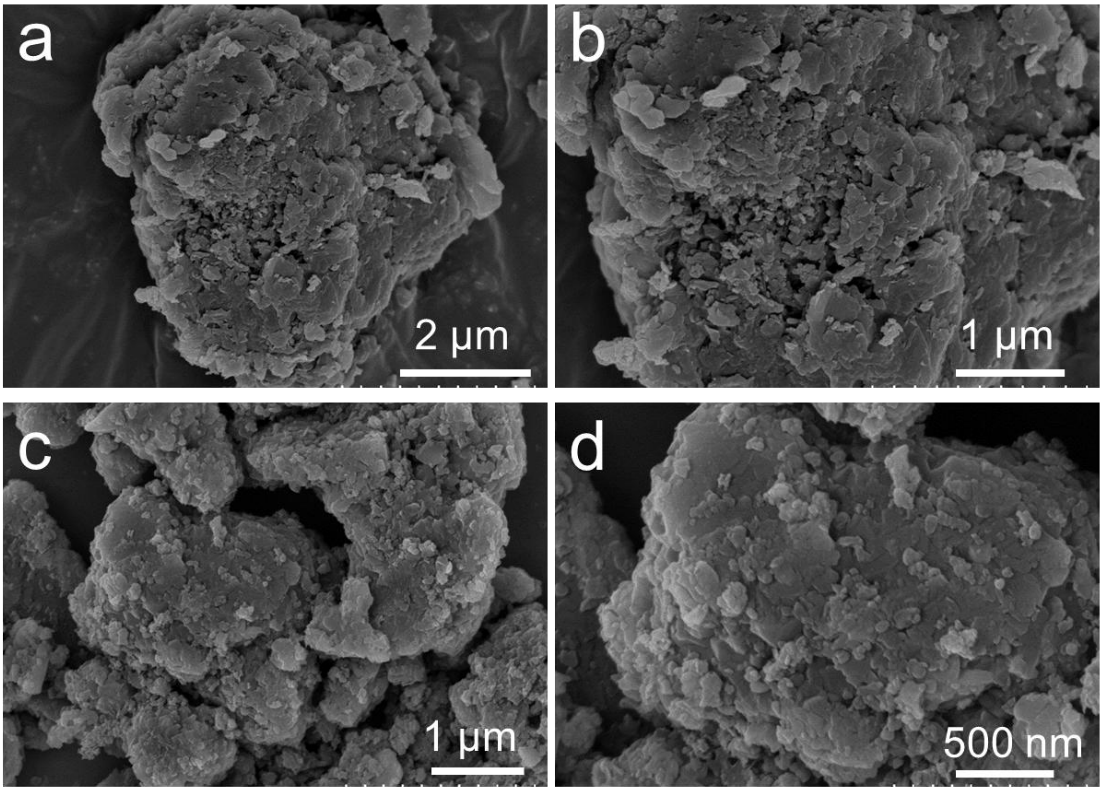

Supplementary Fig. 25. SEM image of the (a-b) CN and (c-d) CN-K.

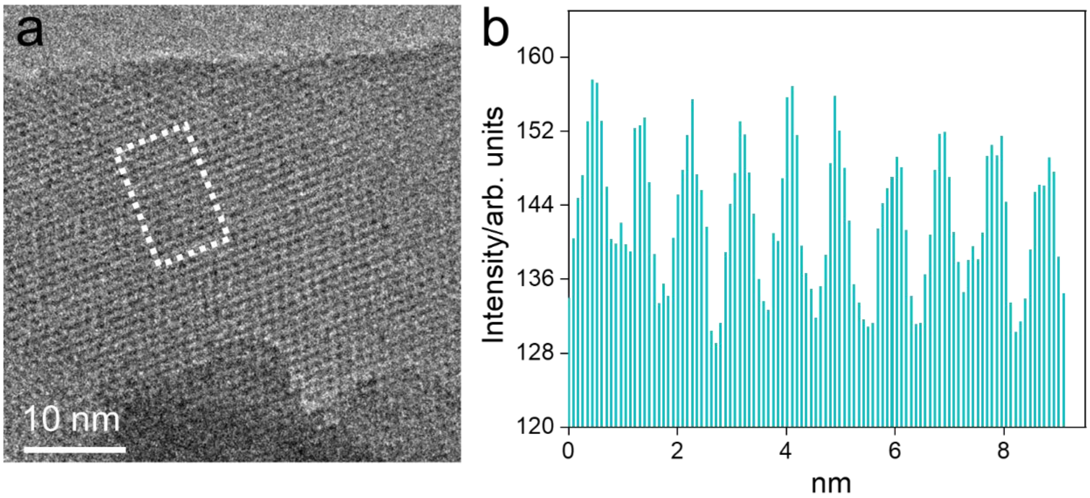

Supplementary Fig. 26. (a) high resolution-TEM of CN-KCs, (b) the rectangle profile in a.

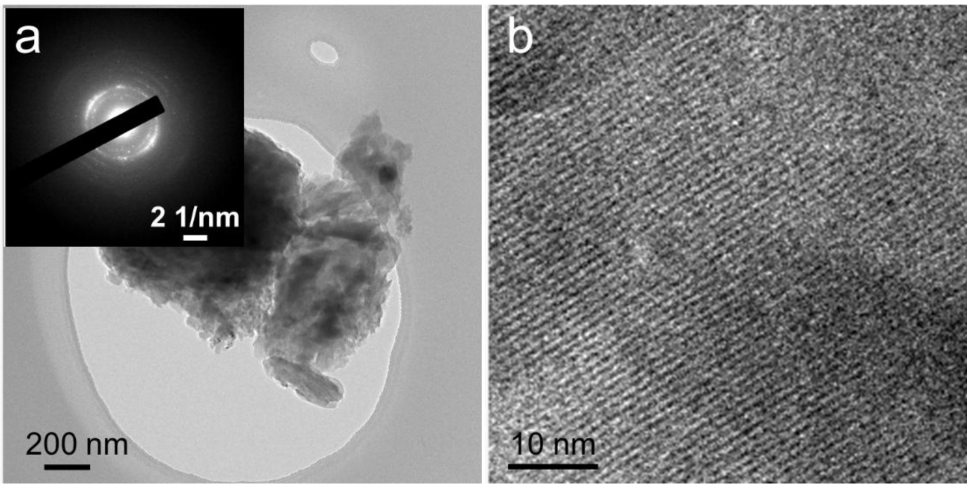

Supplementary Fig. 27. (a)TEM image and (b) high-resolution (HR) TEM image of the CN-K, inset in a is the selected area electron diffraction (SAED) image.

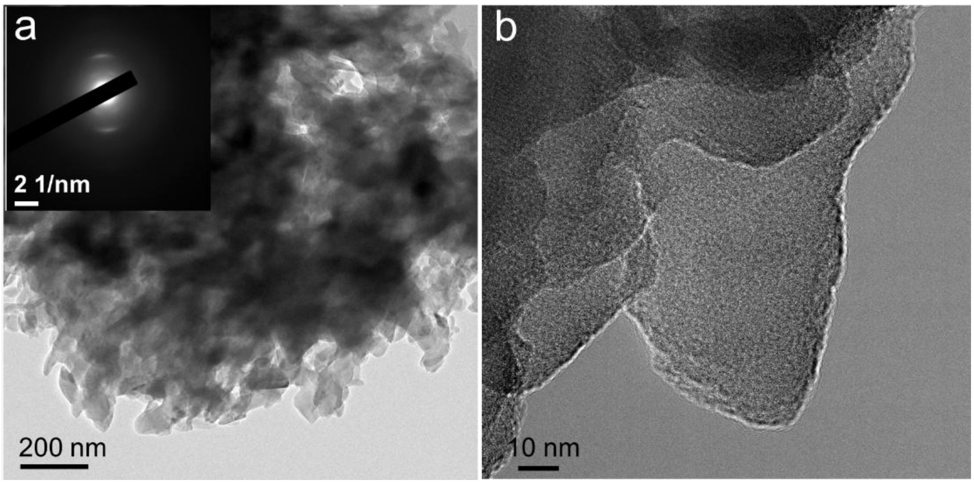

Supplementary Fig. 28. (a)TEM image and (b) high-resolution (HR) TEM image of the CN-Cs, inset in a is the selected area electron diffraction (SAED) image.

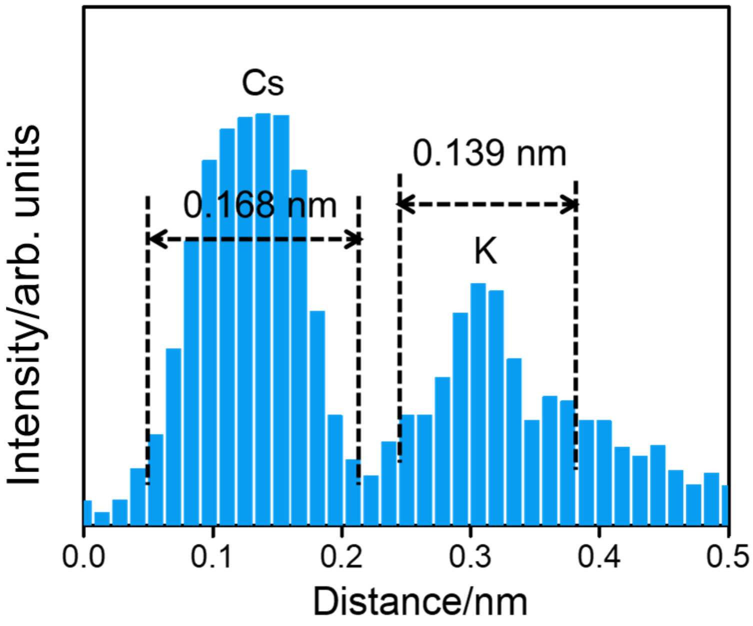

Supplementary Fig. 29. The correspondingly atom-atom distance profile from the image of Fig. 2g.

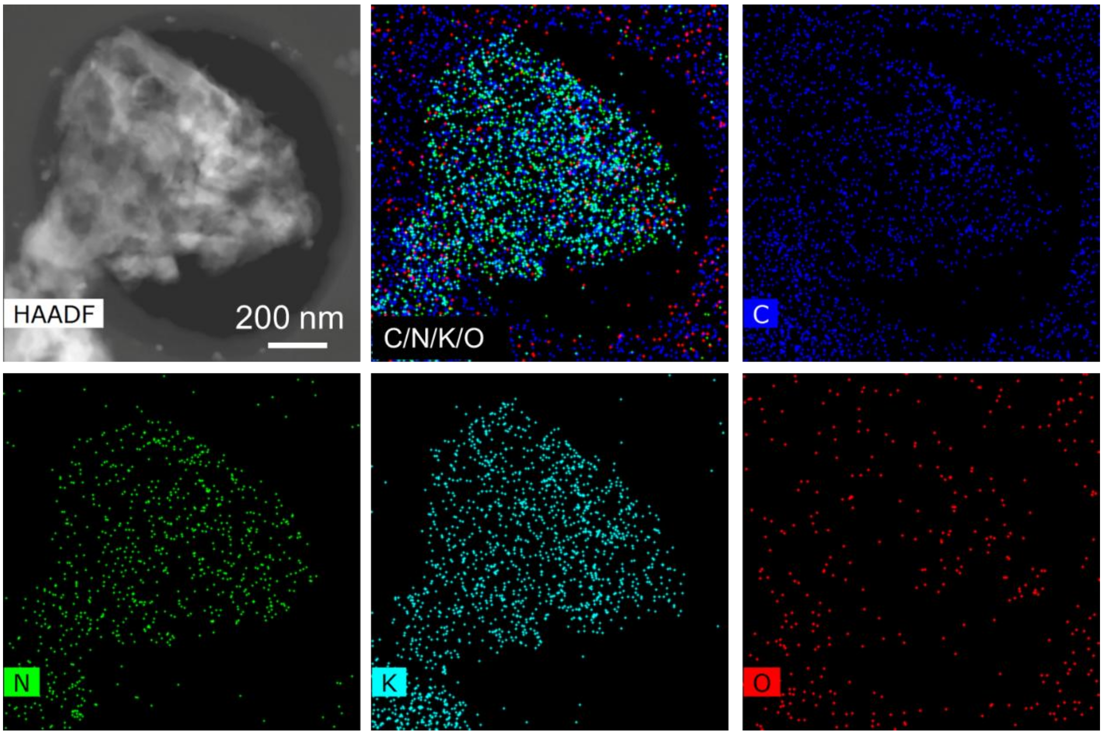

Supplementary Fig. 30. STEM elemental mapping images of C, N, K and O for CN-K.

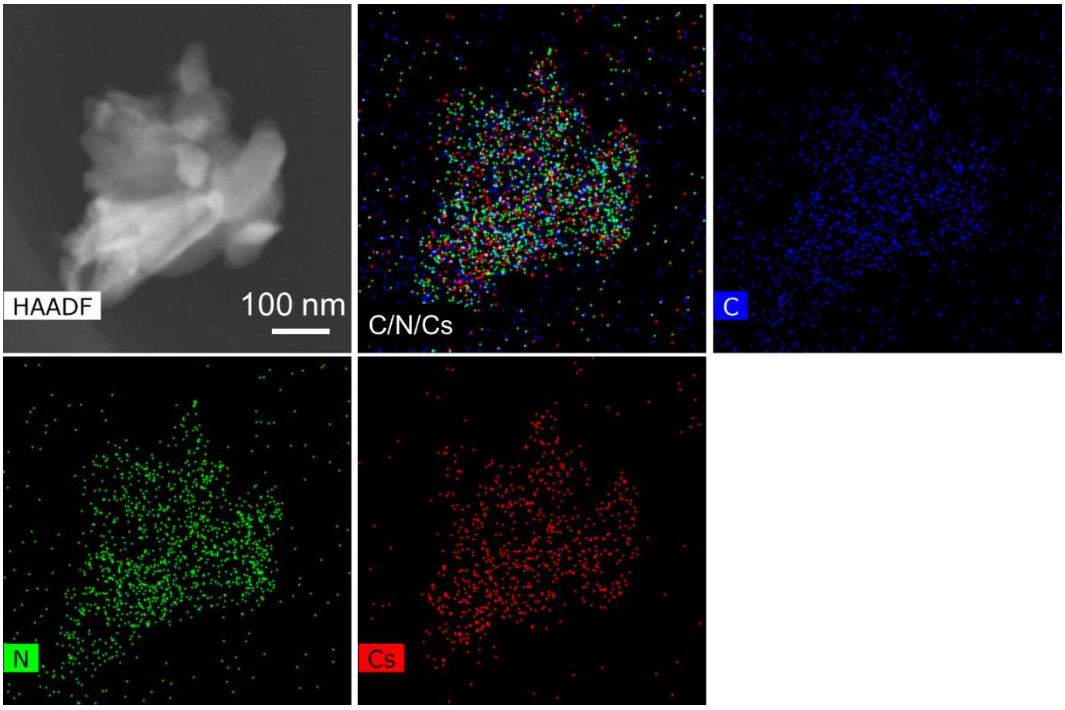

Supplementary Fig. 31. STEM elemental mapping images of C, N, and Cs for CN-Cs.

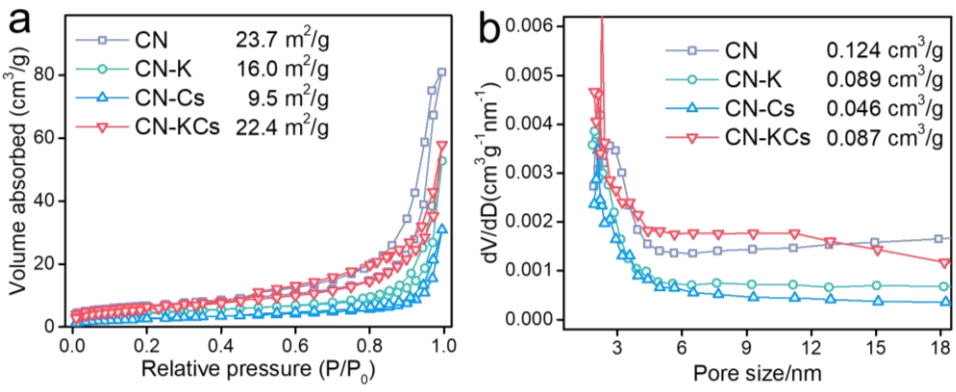

Supplementary Fig. 32. (a) The specific surface area and (b) pore volume of CN, CN-K, CN-Cs and CN-KCs catalysts.

The specific surface area of CN-KCs is measured at 22.4 m^2/g, with a pore volume of 0.087 m^3/g, which is slightly smaller than that of pristine CN but larger than that of CN-Cs (Supplementary Fig. 32). Providing that CN-KCs exhibits the highest photocatalytic performance, it can be concluded that surface area is not the main factor that affects the ORR activity.

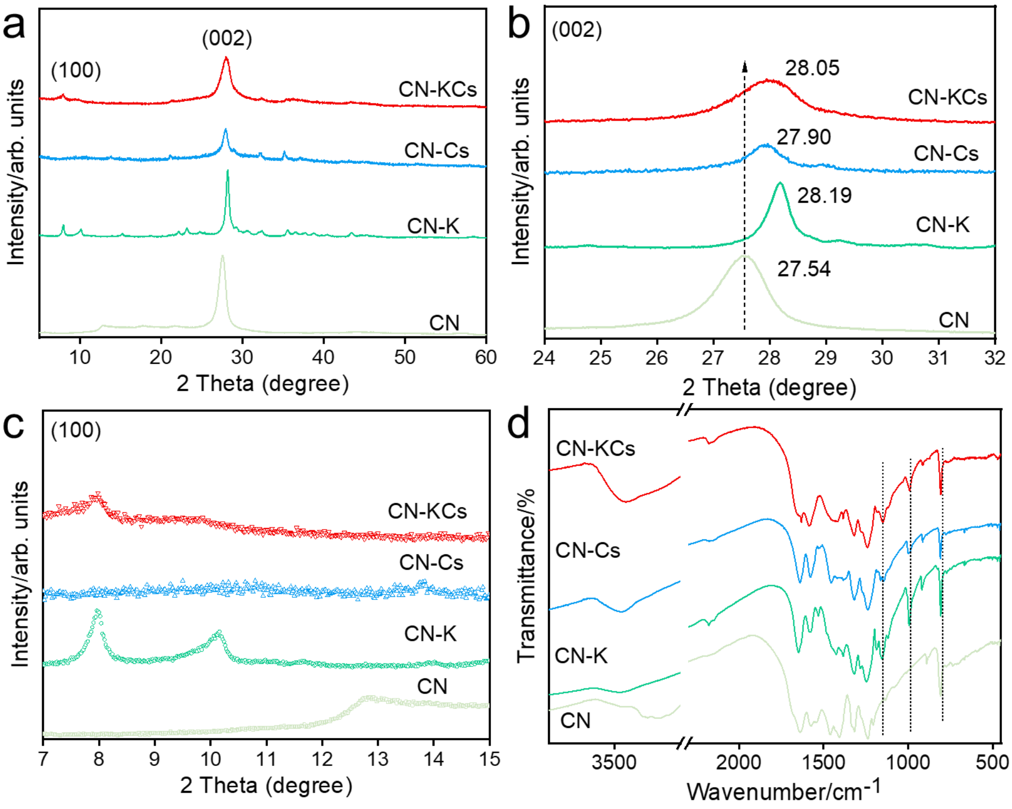

Supplementary Fig. 33. (a) XRD patterns, (b) the enlarged (002) plane and (c) the enlarged (002) plane. (d) FTIR spectra of CN, CN-K, CN-Cs and CN-KCs catalysts.

The XRD pattern and FTIR spectra (Supplementary Fig. 33) of CN-KCs indicate that it retains its characteristic layered structure and the fundamental structural units of polymeric carbon nitride. The (002) peaks for CN-KCs, CN-Cs, and CN-K are observed at 28.05°, 27.90°, and 28.19°, respectively, compared to 27.54° for CN. This shift to higher diffraction angles suggests a reduced interplanar distance following the introduction of alkali metals. Additionally, new FTIR peaks appearing in the range of 980-1000 cm^{-1} in CN-KCs, CN-Cs, and CN-K indicate the formation of K or Cs-N_x conjugated bonding.

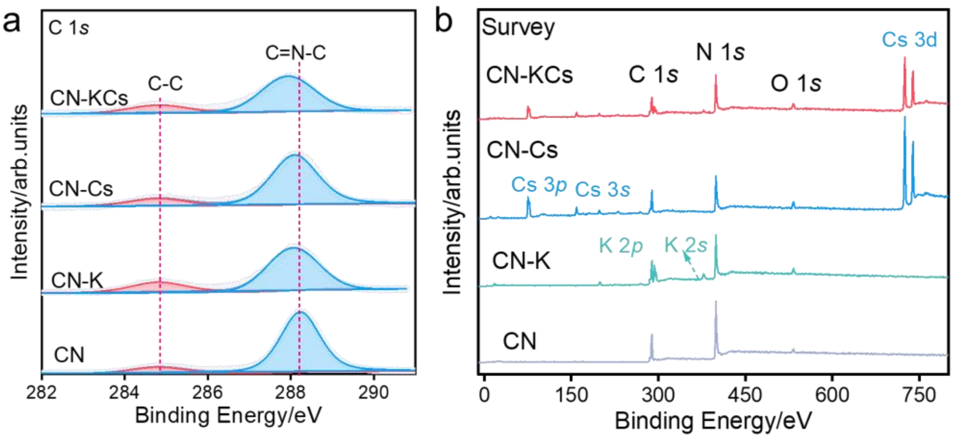

Supplementary Fig. 34. (a) High-resolution XPS spectra of C 1*s* peaks and (b) XPS survey peaks for CN, CN-K, CN-Cs and CN-KCs catalysts.

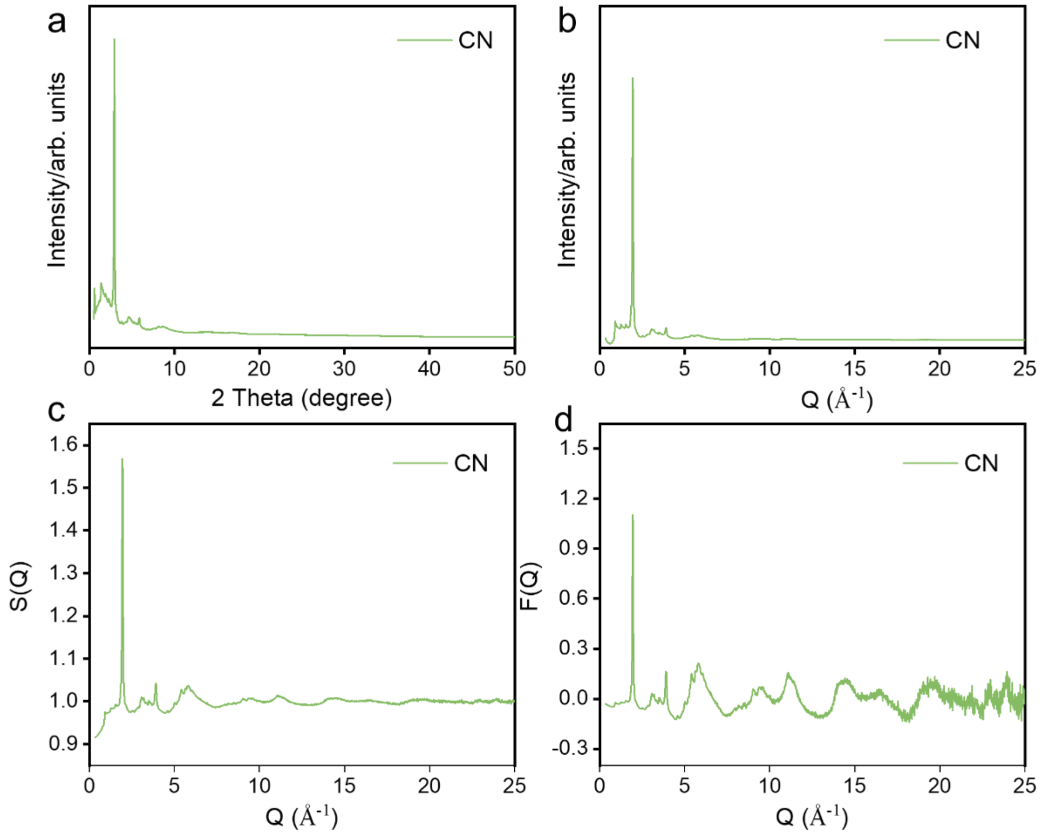

Supplementary Fig. 35. The synchrotron total sacttering data show in (a) 2theta and (b) Q vector (labma = 0.1652 A, energy = 75.052 KeV), (c) Structure factors S(Q), and (d) Reduced total scattering structure functions F(Q) processed w ith Q_{max} = 25 $Å^{-1}$ of CN.

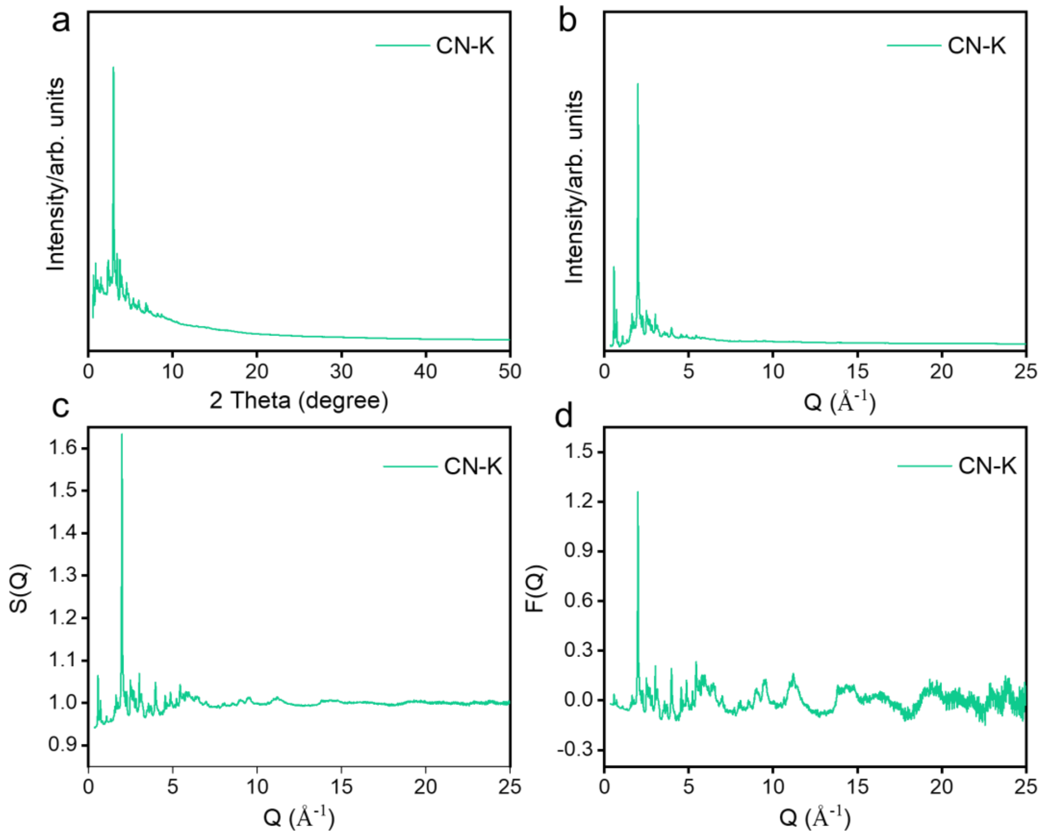

Supplementary Fig. 36. The synchrotron total sacttering data show in (a) 2theta and (b) Q vector (labma = 0.1652 A, energy = 75.052 KeV), (c) Structure factors S(Q), and (d) Reduced total scattering structure functions F(Q) processed w ith Q_{max} = 25 $Å^{-1}$ of CN-K.

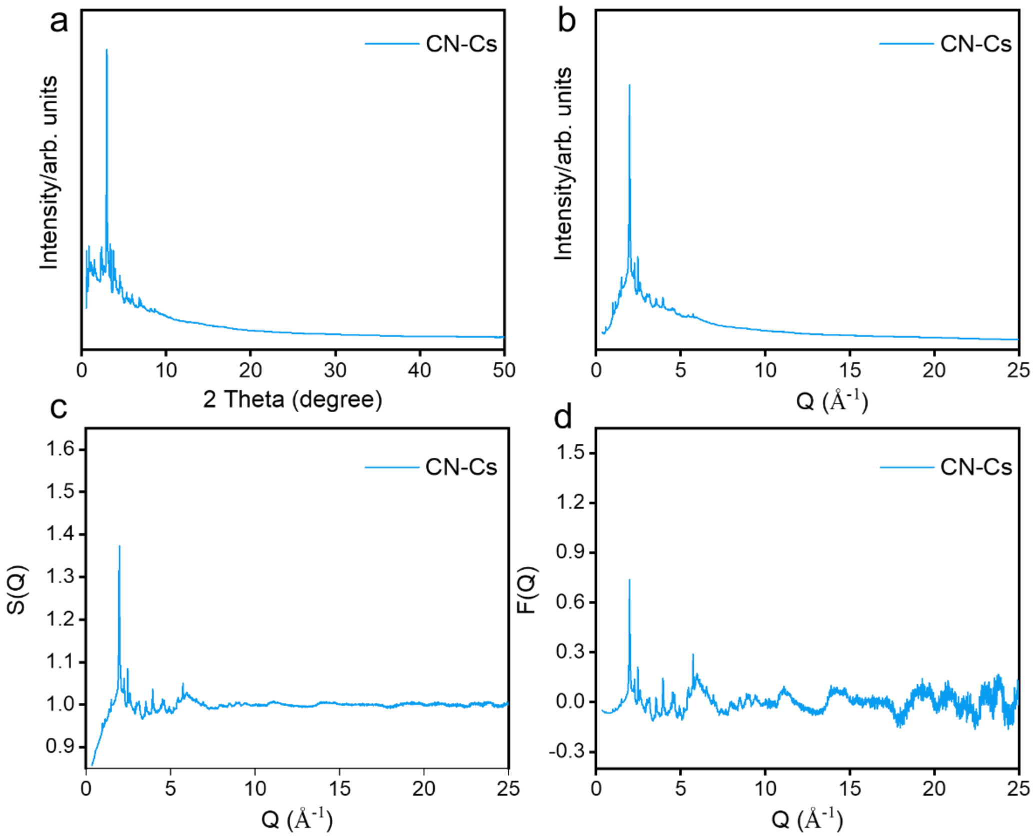

Supplementary Fig. 37. The synchrotron total sacttering data show in (a) 2theta and (b) Q vector (labma = 0.1652 A, energy = 75.052 KeV), (c) Structure factors S(Q), and (d) Reduced total scattering structure functions F(Q) processed w ith Q_{max} = 25 $Å^{-1}$ of CN-Cs.

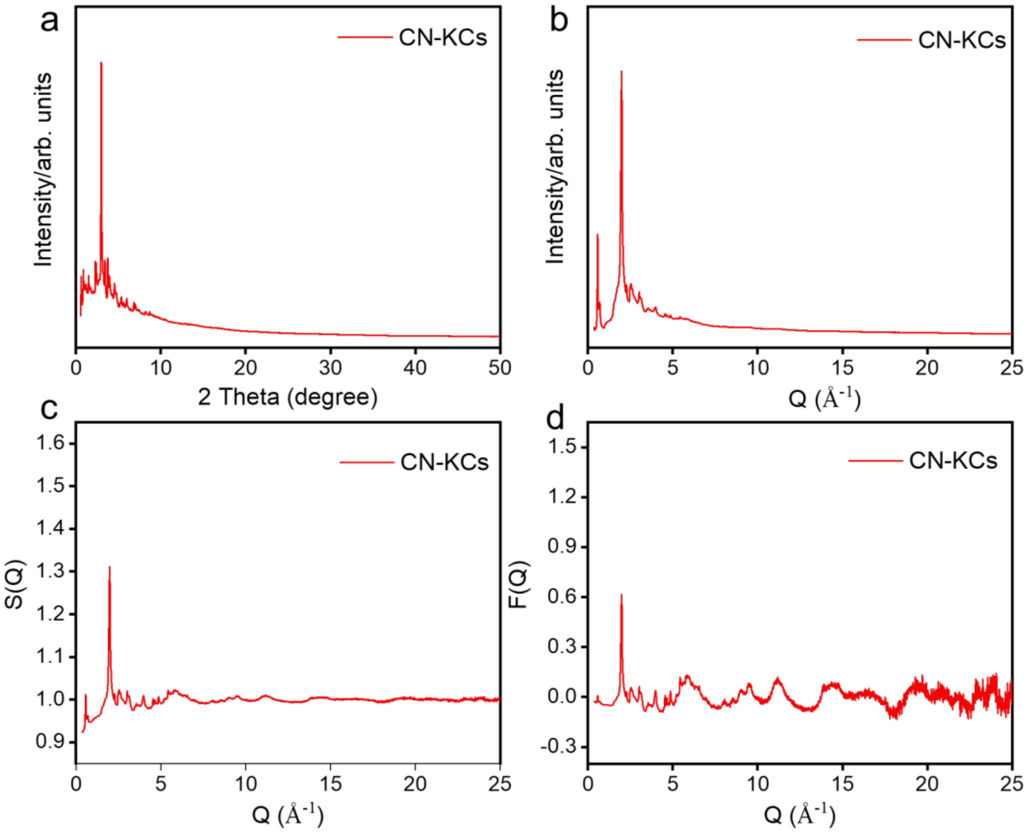

Supplementary Fig. 38. The synchrotron total sacttering data show in (a) 2theta and (b) Q vector (labma = 0.1652 A, energy = 75.052 KeV), (c) Structure factors S(Q), and (d) Reduced total scattering structure functions F(Q) processed w ith Q_{max} = 25 $Å^{-1}$ of CN-KCs.

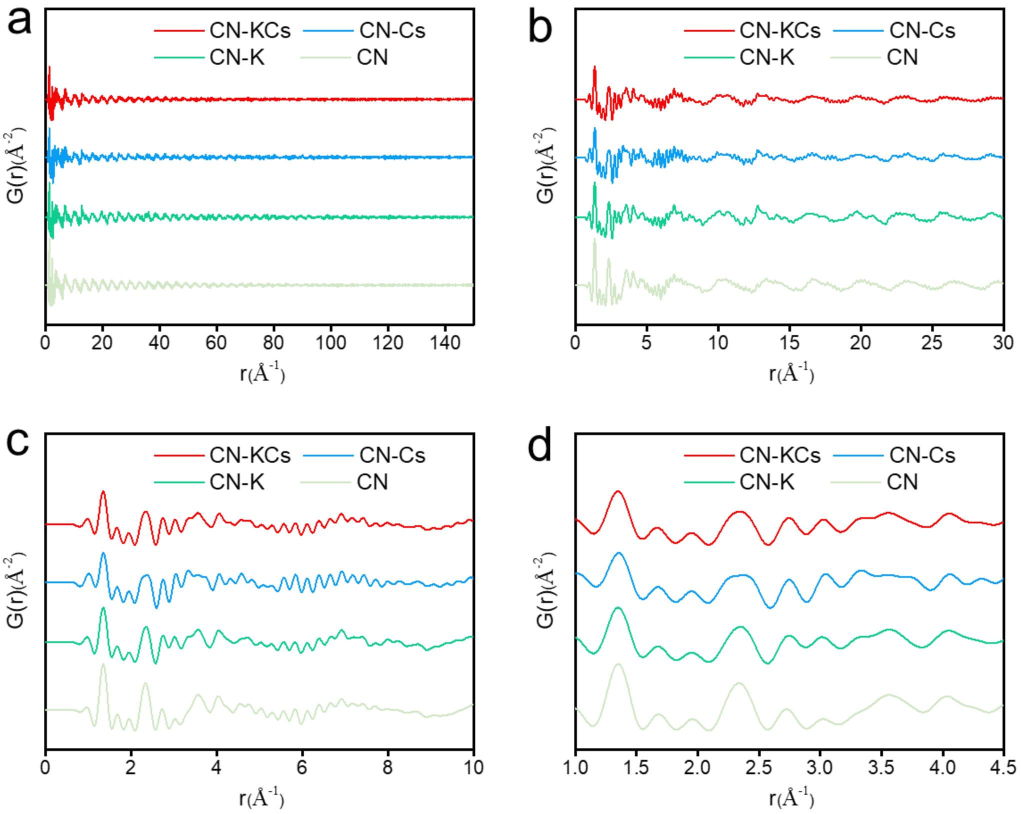

Supplementary Fig. 39. The pair distribution function (G(r)) with different ranges of (a) 0-150, (b) 0-30, (c) 0-10 and 1-4.5 $Å^{-1}$ for CN, CN-K, CN-Cs and CN-KCs catalysts.

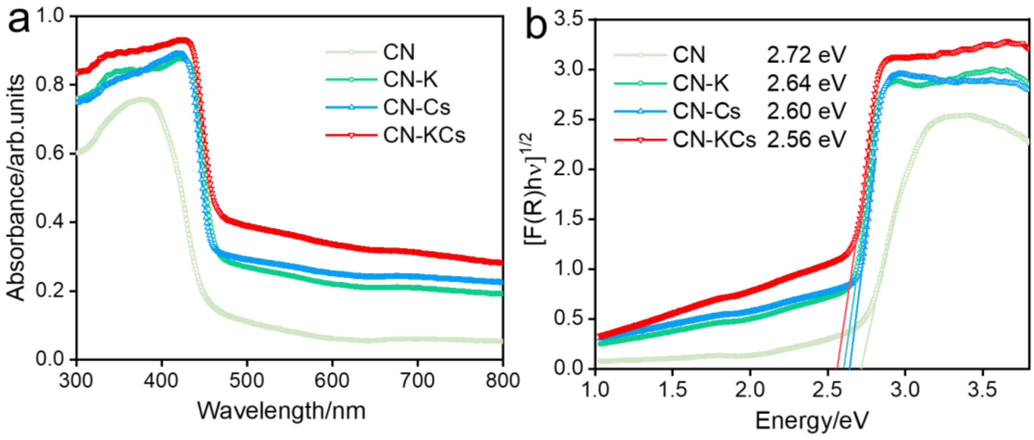

Supplementary Fig. 40. (a) Diffuse reflectance UV-Vis (DRS) spectra and (b) The calculated band gap of catalyst samples converted using the Kubelka-Munk function from DRS spectra of CN, CN-K, CN-Cs and CN-KCs catalysts.

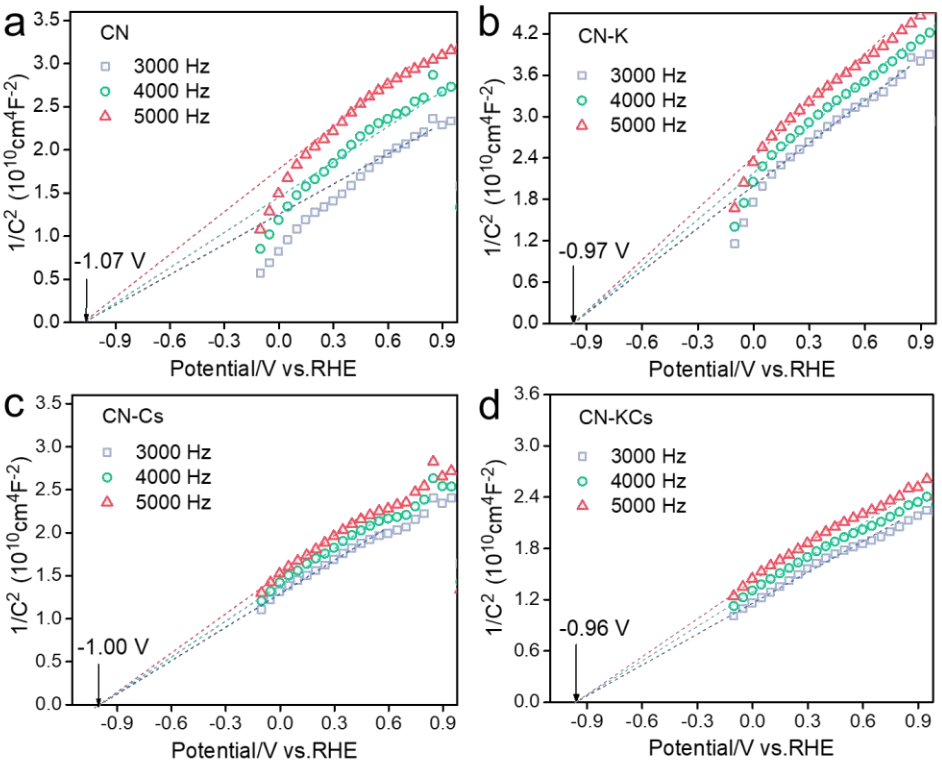

Supplementary Fig. 41. The Mott–Schottky (M–S) plots over the frequency of 3000, 4000, and 5000 Hz of (a) CN, (b) CN-K, (c) CN-Cs and (d) CN-KCs.

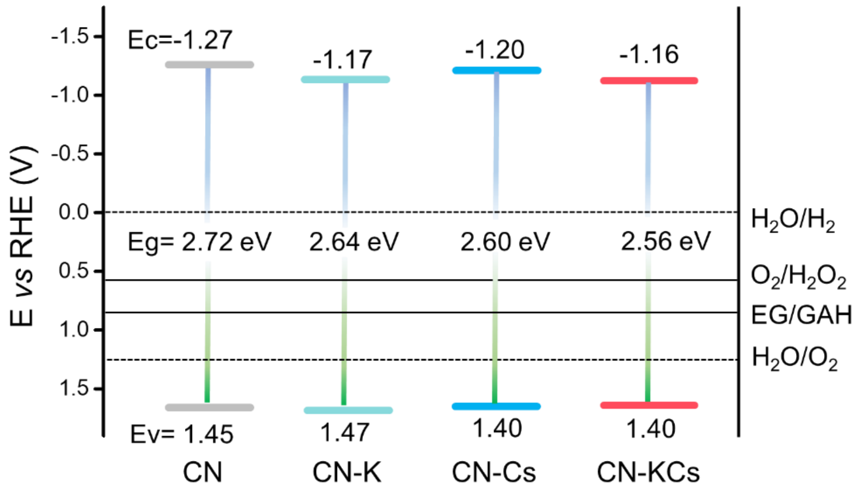

Supplementary Fig. 42. Band structure alignments for CN, CN-K, CN-Cs and CN-KCs catalyst samples.

The optical absorption and band alignment, as shown in Supplementary Figs. 40-42, are demonstrated through the DRS spectra and Mott–Schottky plots. A red shift in the absorption band edge is observed after the introduction of K/Cs. It is revealed the conduction band (CB) of CN-KCs is sufficiently negative to facilitate H_2O_2 generation from O_2 reduction, while the valence band (VB) is positive enough to enable selective oxidation of EG.

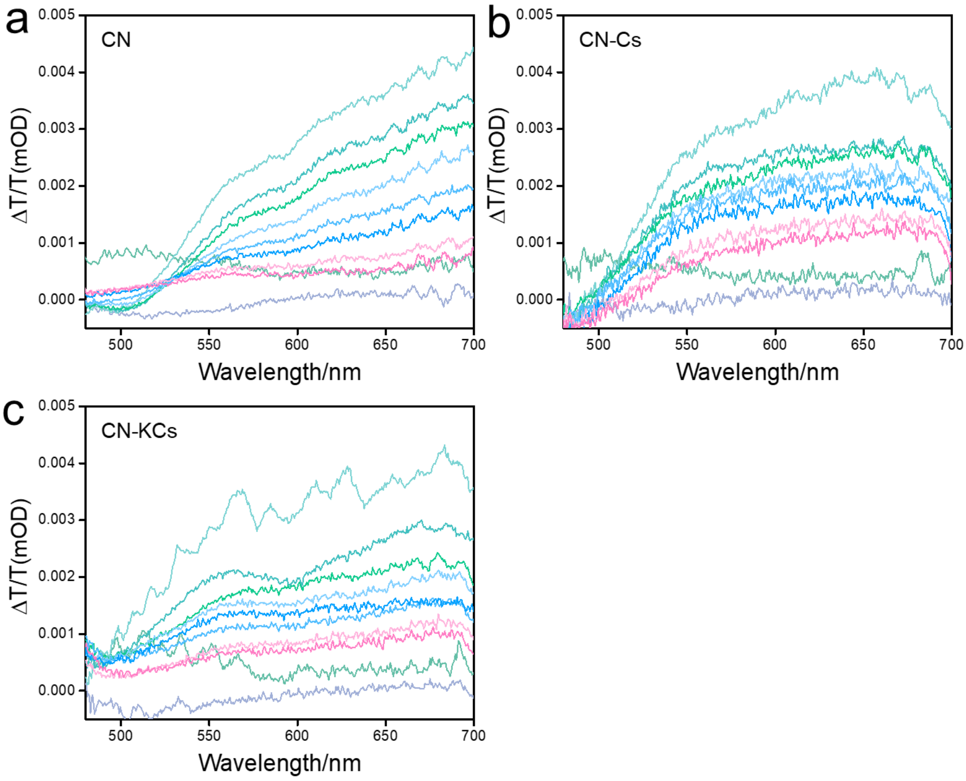

Supplementary Fig. 43. fs-TA decay spectra from approximately 0.2 ps to 500 ps among (a) CN, (b) CN-Cs and (c) CN-KCs catalyst.

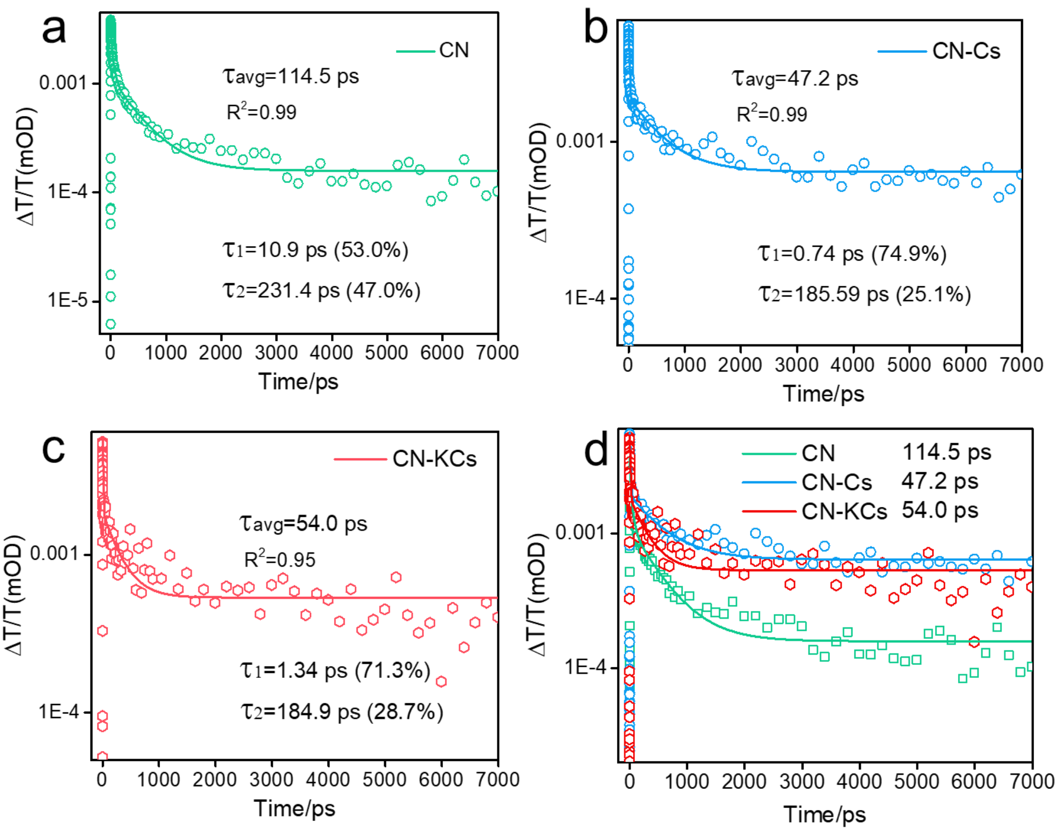

Supplementary Fig. 44. fs-TA decay spectra at approximately 600 nm over (a) CN, (b) CN-Cs, (c) CN-KCs catalyst and (c) the merge of three catalyst in one picture.

From the fs-TA spectra (Fig. 3a-3c and Supplementary Figs. 43-44), during the early decay phase, specifically within less than 10 ps, the intensity of excited-state absorption (ESA) in the region of approximately 520-700 nm for CN-Cs and CN-KCs is stronger than that of CN. This indicates a generation of more excited electrons and a broader spectral range of active responses, which is consistent with the AQE activities of CN and CN-KCs at different wavelengths. CN exhibits a slow decay rate within this timeframe, suggesting a low density of shallow traps within its molecular structure. In this case, excited electrons are more likely to undergo direct recombination or transfer to deeper traps, resulting in fewer excited electrons available for participation in catalytic reactions and, thus, lower reactivity. In CN-Cs and CN-KCs, the rapid decay of charge carriers (<10 ps) than CN signifies that excited electrons can be swiftly transferred. Over the decay period of 20 ns to 500 ns, CN-Cs displays a markedly slower decay process, indicating that deep traps begin to dominate the behavior of the carriers, allowing electrons to be stored within these traps for extended lifetimes. In contrast, CN-KCs

exhibits a slightly faster carrier decay rate, suggesting that the incorporation of K optimizes the synergistic transport of electrons between shallow and deep traps, thus continuously supplying excited electrons for facilitating surface reactions.

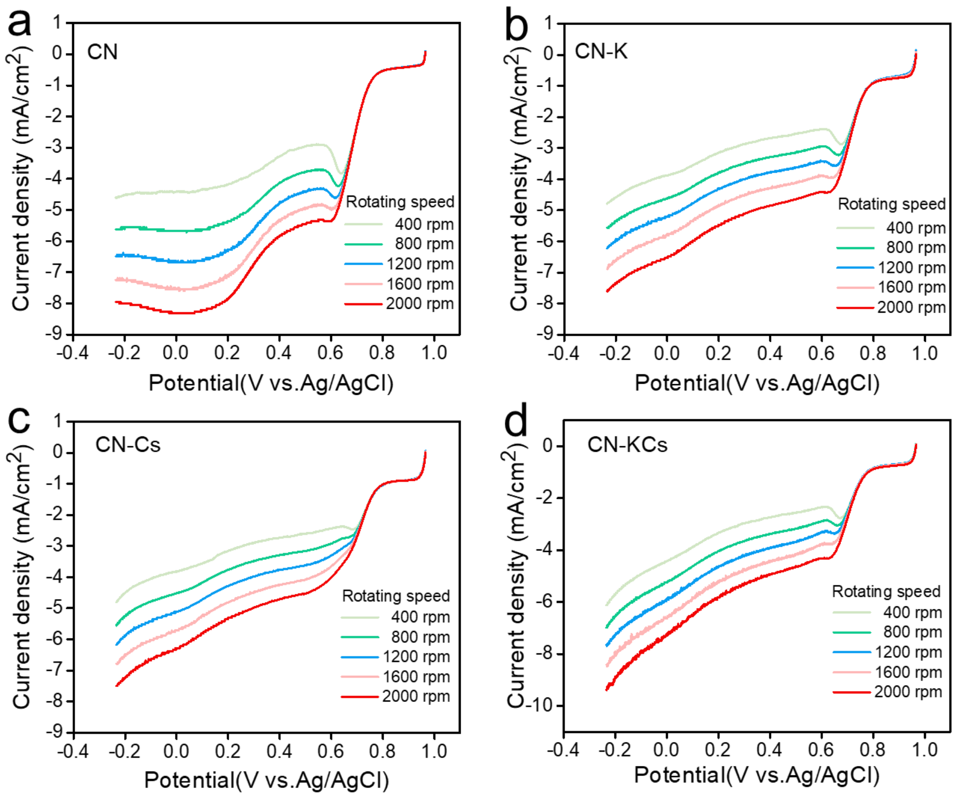

Supplementary Fig. 45. LSV of (a) CN, (b) CN-K, (c) CN-Cs, and (d) CN-KCs film electrodes in O_2-saturated 0.1 M $NaClO_4$ measured on RDE at different rotating speeds.

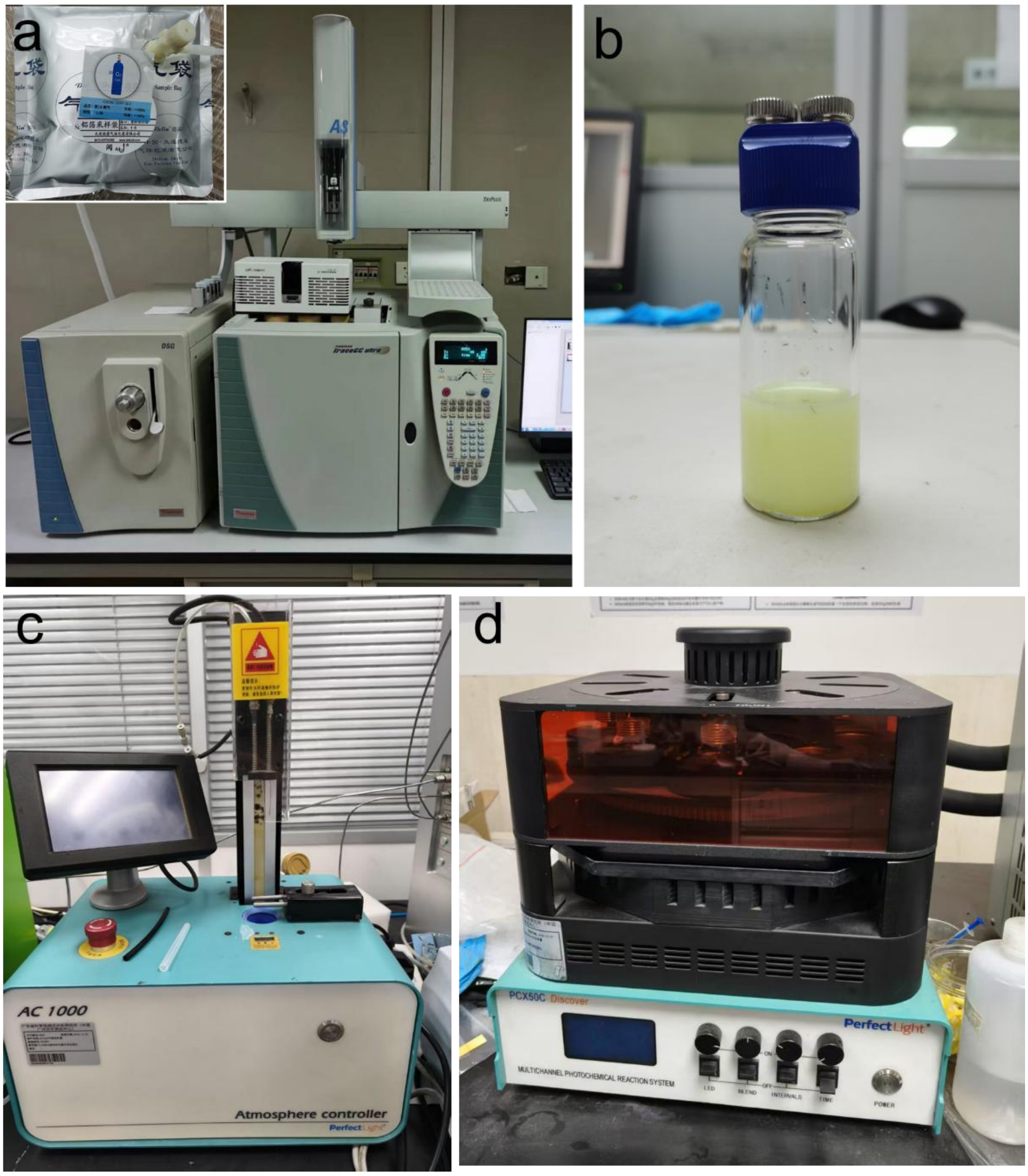

Supplementary Fig. 46. Digital picture of (a) Agilent 6890N-5973 inert gas chromatography−mass spectrometry, (b) reaction reactor. Inset is $^{18}O_2$ gas pocket, (c) Atmosphere controller and (d) photocatalytic reaction apparatus.

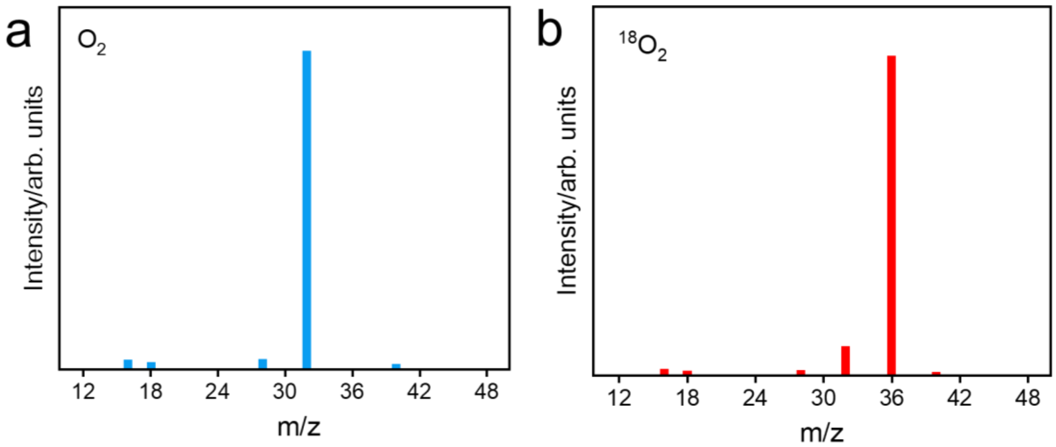

Supplementary Fig. 47. MS spectra of (a) pure O_2 and (a) >99% $^{18}O_2$ gas as comparison.

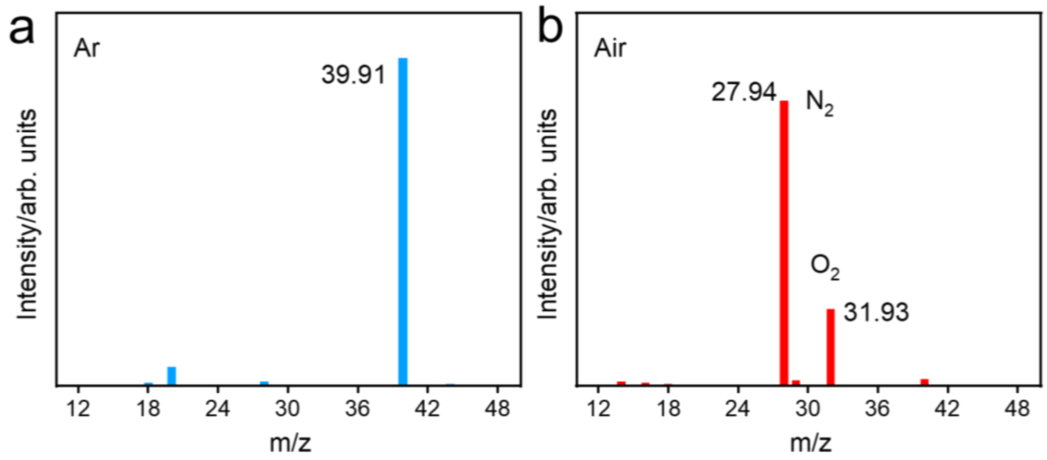

Supplementary Fig. 48. MS spectra of (a) pure Ar gas and (b) air gas. Noted that based on Ar mass specturm, we used Ar as the background and subtracted the Ar mass spectrum peaks to obtain the mass spectrum of the tested gas.

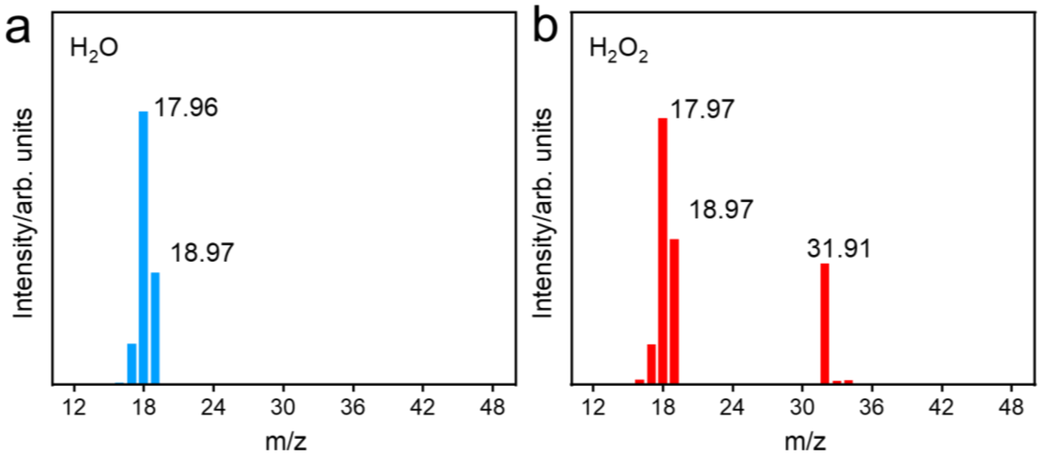

Supplementary Fig. 49. MS spectra of (a) H_2O and (b) H_2O_2 aqueous solution as comparison.

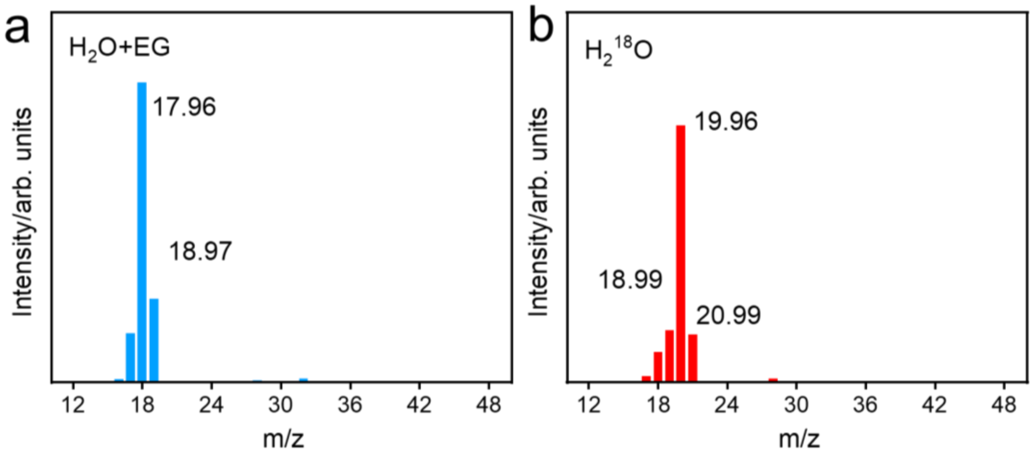

Supplementary Fig. 50. MS spectra of (a) 0.5% (v/v) EG aqueous solution and (b) $H_2{}^{18}O$ agent as comparison.

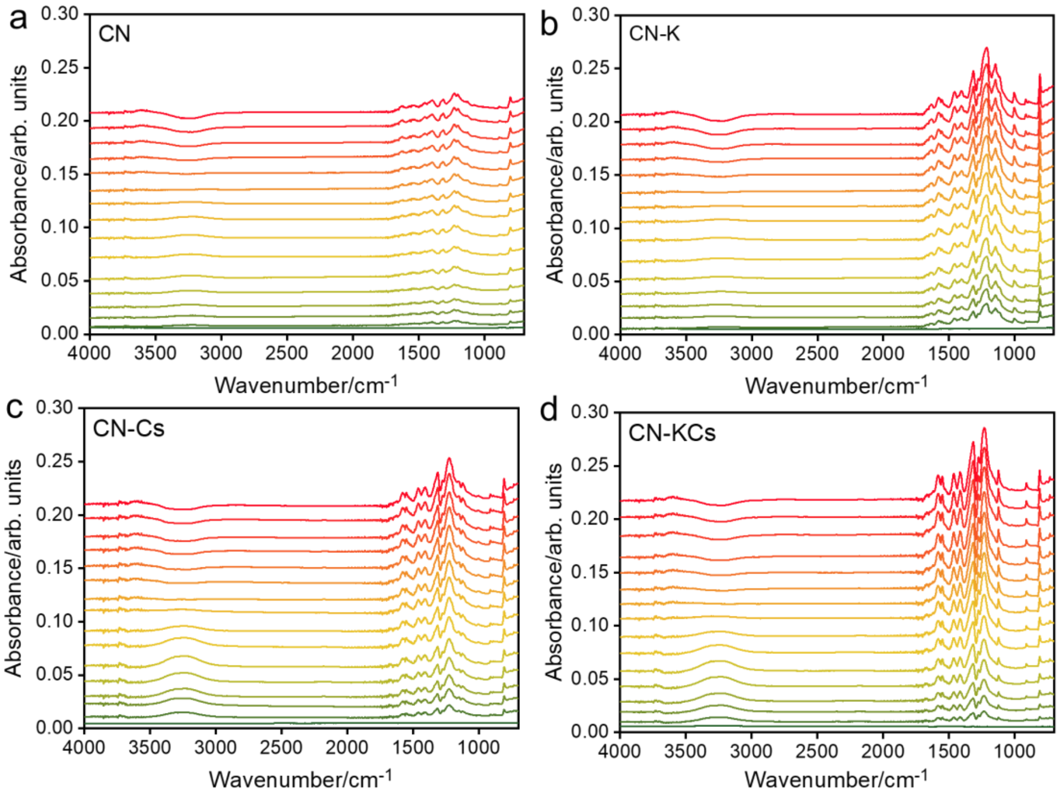

Supplementary Fig. 51. In situ attenuated total reflectance ATR-FTIR experiments of (a) CN, (b) CN-K, (c) CN-Cs, (d) CN-KCs in the light time over the region of 700 to 4000 cm^{-1}, the FTIR spectrum was collected before (0 min) and after purging air gas into sample cup at 5 min intervals.

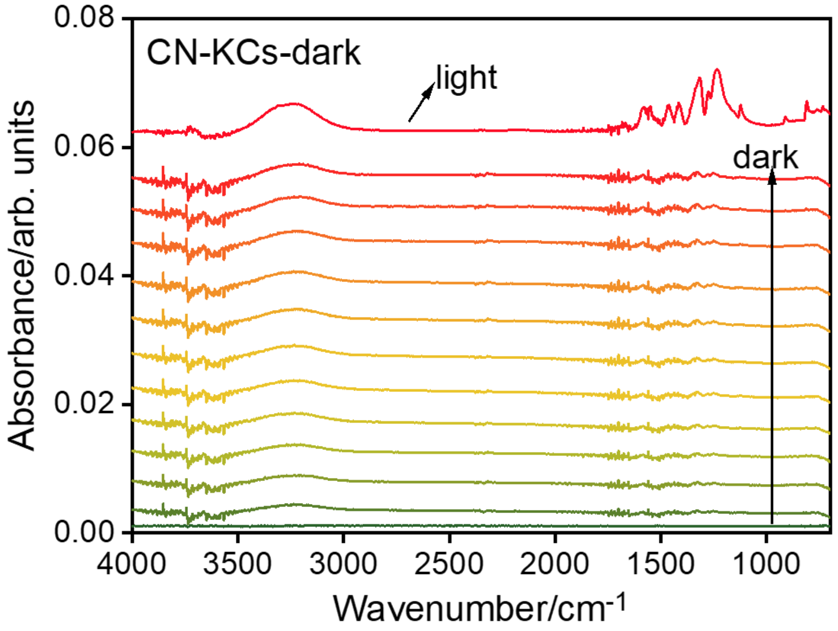

Supplementary Fig. 52. In situ attenuated total reflectance ATR-FTIR experiments of CN-KCs in the dark time over the region of 700 to 4000 cm^{-1}, the FTIR spectrum was collected before (0 min) and after purging air gas into sample cup at 5 min intervals.

These findings indicate that all catalysts yield the final H_2O_2 product, with CN-KCs demonstrating the most significant intensity of the $*H_2O_2$ FTIR peak, suggesting higher catalytic activity. Conversely, CN exhibits the weakest peak intensity, correlating with the lowest H_2O_2 production activity, consistent with previous activity data. Additionally, the FTIR peaks associated with the characteristic intermediates of the ORR demonstrate a gradual increase in intensity with prolonged light illumination in a certain time. In contrast, under dark conditions, there are virtually no corresponding signals (Supplementary Fig. 52), indicating that the observed intermediates peaks for the final H_2O_2 production are driven by incident photons.

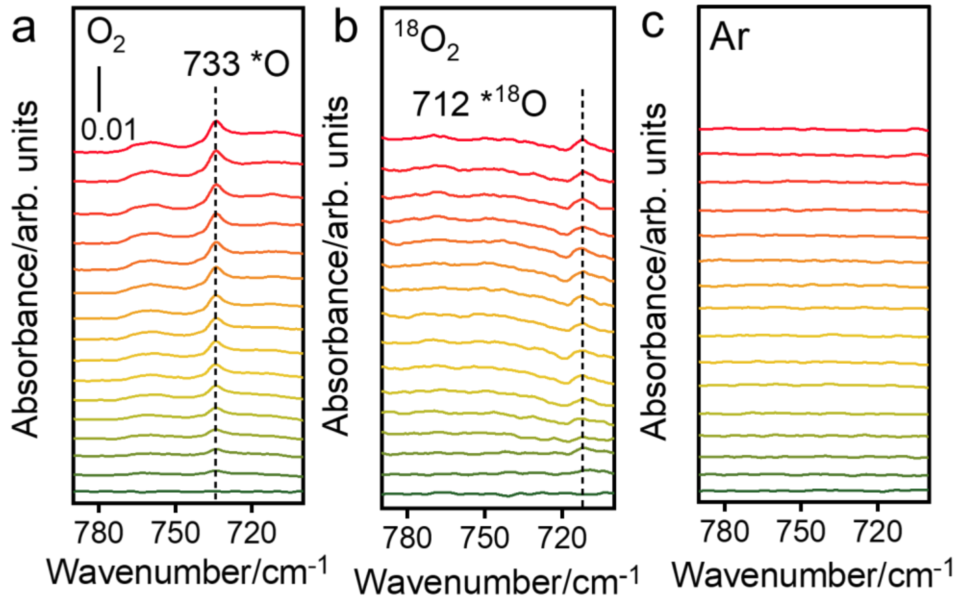

Supplementary Fig. 53. In situ ATR-FTIR experiments for CN-KCs under visible light illumination, conducted under (a) O_2 gas, (b) ^{18}O-labeled O_2 gas and (c) Ar gas. FTIR spectra were collected at 5-minute intervals.

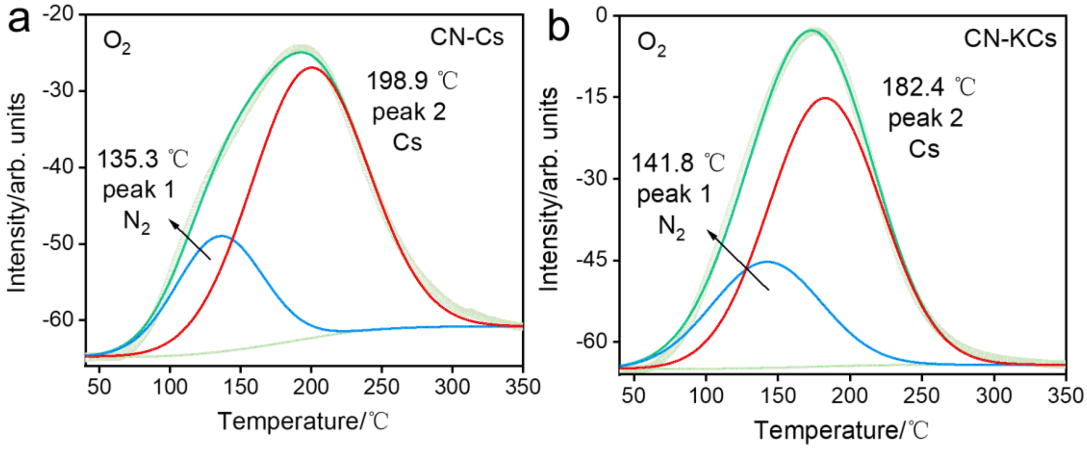

Supplementary Fig. 54. Temperature-programmed desorption (TPD)-O_2 spectra of (a) CN-Cs and (b) CN-KCs. The original spectrum was fitted into two peaks: one is the adsorption peak of the intrinsic N sites of CN, and the other peak, represented by the red line, corresponds to the adsorption peak of O_2 at the Cs sites.

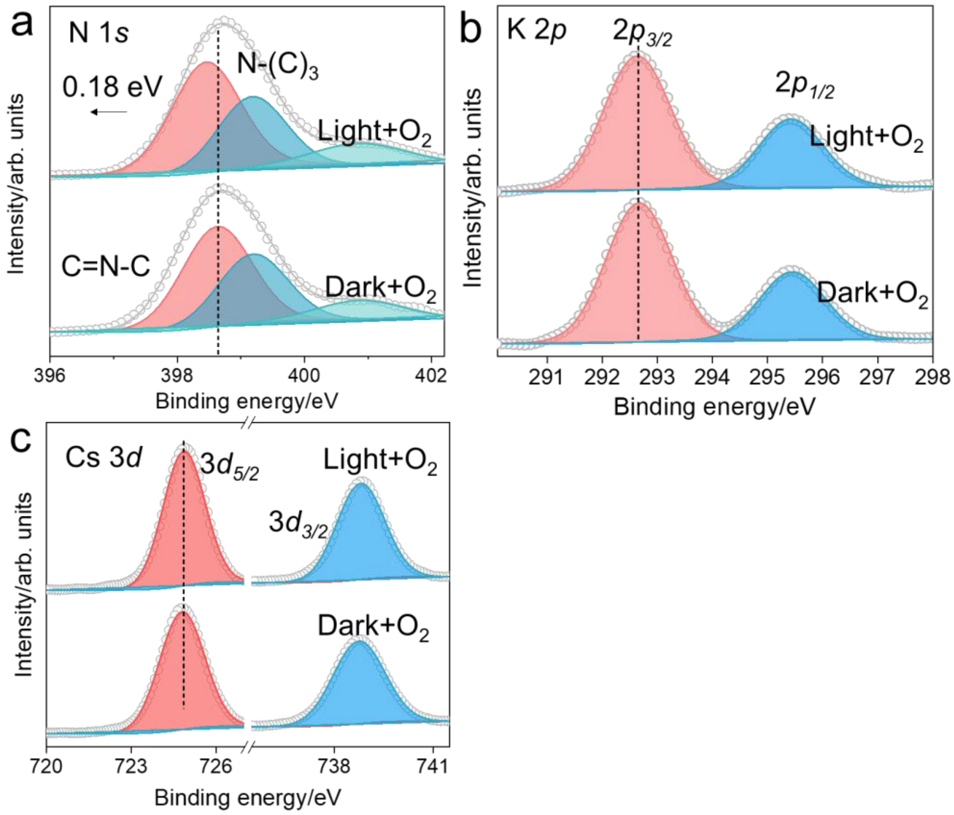

Supplementary Fig. 55. In situ XPS experiments of **a.** N 1*s*, **b.** K 2*p* and **c.** Cs 3*d* spectra under the O_2 gas with dark time or illumination on CN-KCs.

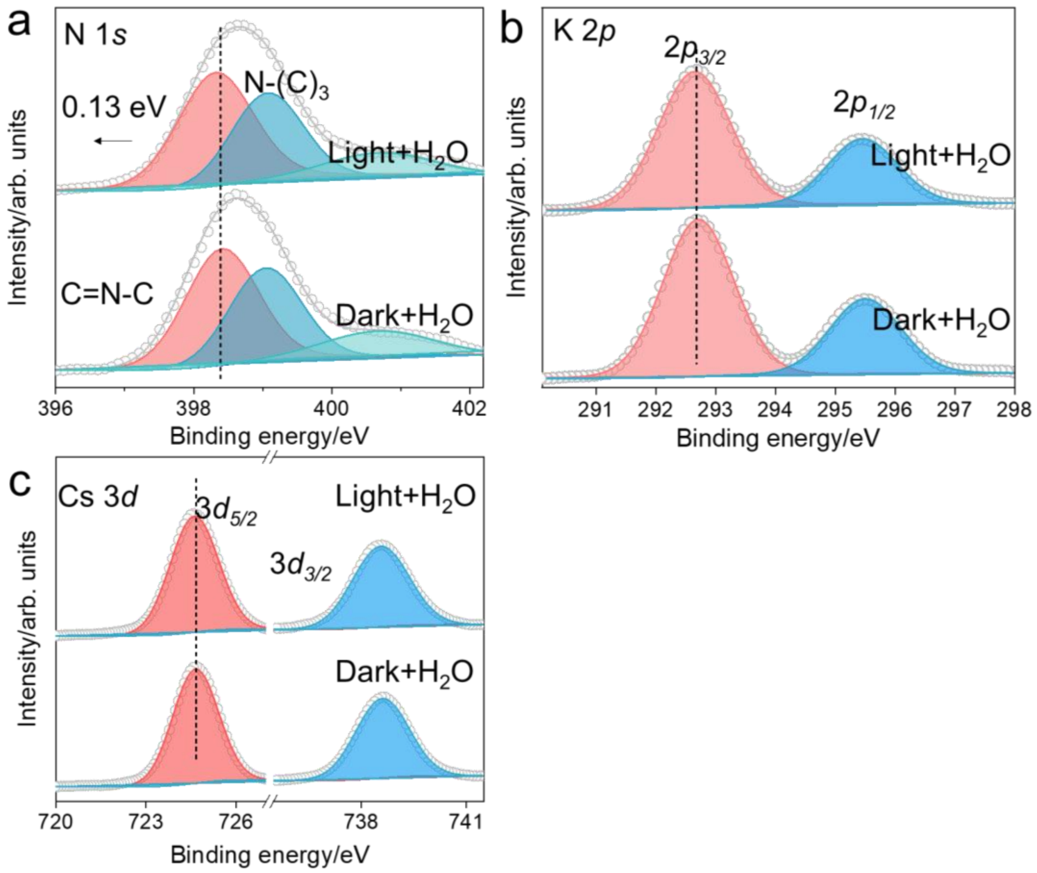

Supplementary Fig. 56. In situ XPS experiments of **a.** N 1*s*, **b.** K 2*p* and **c.** Cs 3*d* spectra under the H_2O steam with dark time or illumination on CN-KCs.

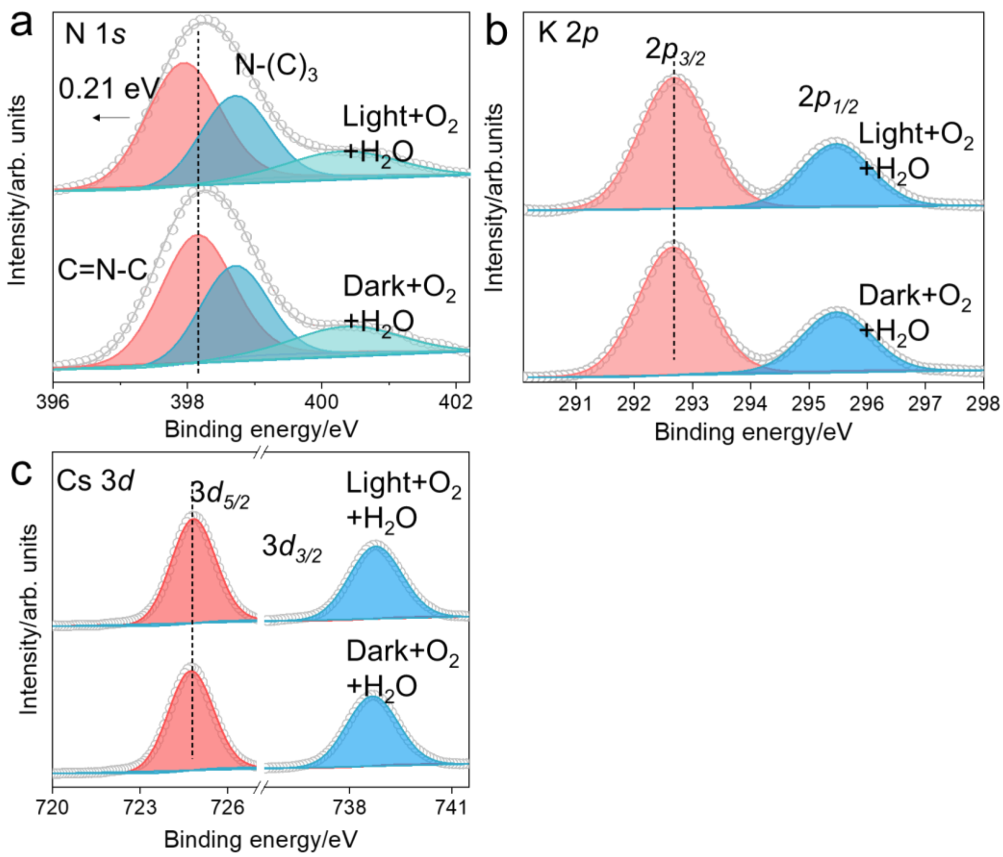

Supplementary Fig. 57. In situ XPS experiments of **a.** N 1*s*, **b.** K 2*p* and **c.** Cs 3*d* spectra under the O2+H_2O with dark time or illumination on CN-KCs.

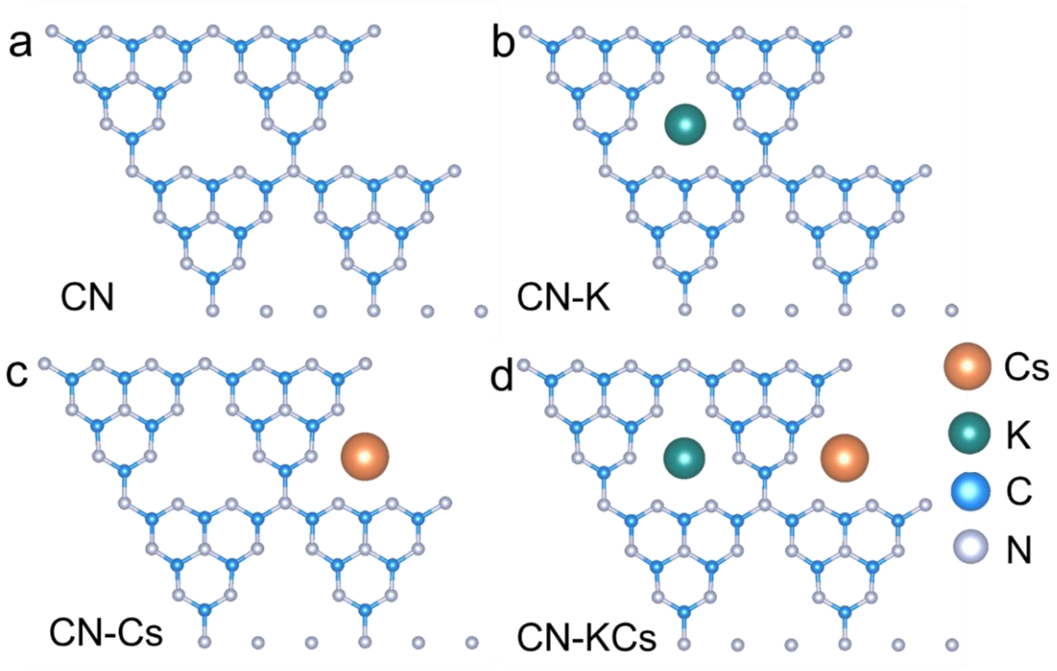

Supplementary Fig. 58. Proposed molecular model of (a) CN, (b) CN-K, (c) CN-Cs and (d) CN-KCs. The grey, blue, red, green, and orange color sphere denote the carbon, nitride, copper, potassium and cesium atom respectively.

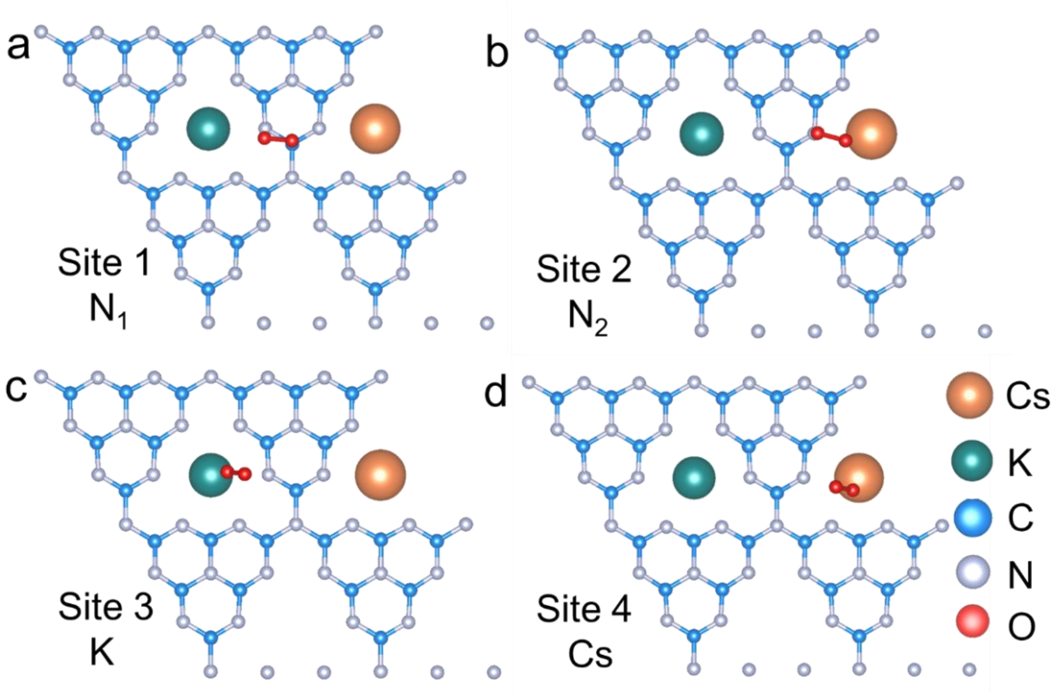

Supplementary Fig. 59. Proposed molecular model for the adsorption of O_2 on (a) site 1 (N_1), (b) site 2 (N_2), (c) site 3 (K) and (d) site 4 (Cs) on CN-KCs. The grey, blue, red, green, orange and red color sphere denote the carbon, nitride, copper, potassium, cesium and oxygen atom respectively.

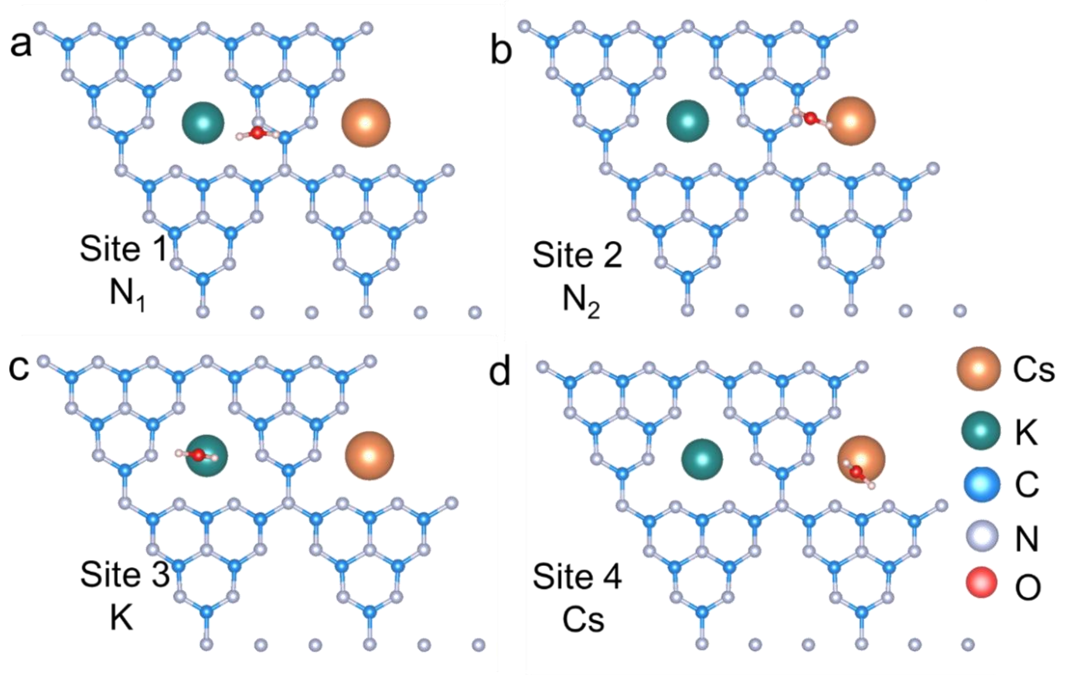

Supplementary Fig. 60. Proposed molecular model for the adsorption of H_2O on (a) site 1 (N_1), (b) site 2 (N_2), (c) site 3 (K) and (d) site 4 (Cs) on CN-KCs. The grey, blue, red, green, orange and red color sphere denote the carbon, nitride, copper, potassium, cesium and oxygen atom respectively.

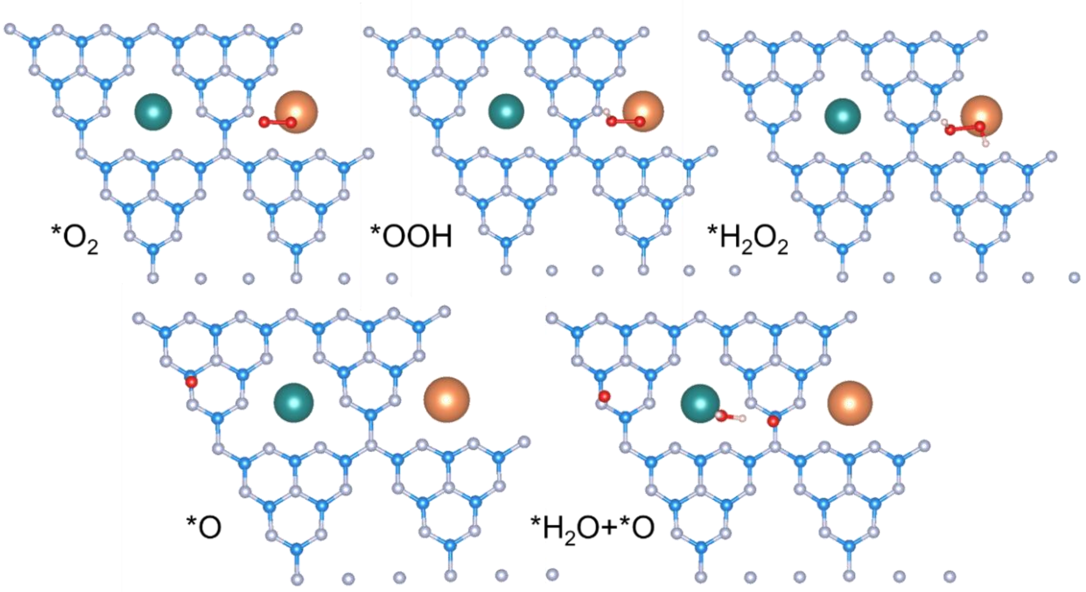

Supplementary Fig. 61. Proposed molecular model for the ORR reaction pathway on CN-KCs. The grey, blue, red, green, orange and red color sphere denote the carbon, nitride, copper, potassium, cesium and oxygen atom respectively.

3. Tables

Table S1. The data statistics show the solar to chemical conversion rate (SCC) and apparent quantum yield (AQY) of different catalysts under their respective reaction conditions., the light source is 300 W Xe light or white LED from 50-150 mW/cm^2, compared with that of CN-KCs.

Entry	Catalyst	Condition	Rate[a]	AQE[b]	SCC(%)	Ref
1	CN-KCs, 15 mg	EG 0.5%, air, 20 ml	1806.6	72.3%	6.1	Our work
2	AKMT, 10 mg	IPA 10%, air, 27 ml		40%	2.2	1
3	RF-DHAQ-2, 10 mg	H_2O, O_2, 50 ml		11.6%	1.20	2
4	COF-TfpBpy, 5 mg	H_2O, air, 10 ml		8.1%	0.57	3
5	TTP, 10 mg	H_2O, air, 50 ml	31.3	7.61%	0.35	4
6	TP-DPBD$_{30}$-COF, 2.5 mg	H_2O, O_2, 15 ml	18	18.0%	0.91	5
7	Hz-TP-BT-COF, 3 mg	H_2O, air, 15 ml	21.3	17.5%	1.21	6
8	CD-AQ, 2 mg	ETOH 10%, pH=6, O_2, 4 ml		26.4%	0.18	7
9	Fe SAS-TpPP-COF, 5 mg	BA 10%, air, 50 ml	20.6	6.4%	0.2	8
10	LCAP-2, 10 mg	H_2O, O_2, 20 ml		9.1%	0.59	9
11	Assembled TCPP, 50 mg	pH=4.0, H_2O,O_2, 50 ml		14.9%	1.20	10
12	RF523, 30 mg	H_2O, O_2, 50 ml		7.5%	0.50	11
13	$CoO_x/BiVO_4$/(Ag/Pd), 50 mg	pH=6.8, O_2, 50 ml		13.1%	0.73	12
14	COF-2CN, 12.5 mg	H_2O, air, 50 ml	20.1	6.8%	0.60	13
15	Ni_{SAPs}-PuCN, 30 mg	H_2O, O_2, 30 ml		10.9%	0.82	14
16	Cu_3-BT-COF, 5 mg	FFA 0.5%,O_2, 30 ml	935	8.0%	0.62	15
17	Sv-ZIS, 20 mg	H_2O, O_2, 30 ml	85.2	9.9%	0.81	16
18	TPC-3D, 1 mg	H_2O, air, 50 ml	9.9	44.8%	3.6	17
19	CNIO-GaSA, 1-50 mg	H_2O, O_2, 2-50 ml	6.6	7.1%	0.4	18
20	CNW03, 10 mg	H_2O, O_2, 100 ml	5.56	8.53%	0.31	19
21	CN-PDA, 30 mg	H_2O, air, 20 ml	496.9	5.1%	0.14	20

[a]:μmolh^{-1}; [b]:λ=420 nm

4. References

[1] P. Zhang, Y.W. Tong, Y. Liu, J.J.M. Vequizo, H.W. Sun, C. Yang, A. Yamakata, F.T. Fan, W. Lin, X.C. Wang, W. Choi, Heteroatom dopants-promoted two-electron O_2 reduction for photocatalytic production of H_2O_2 on polymeric carbon nitride, Angew. Chem. Int. Ed. 132 (2020) 16343-16351.

[2] C. Zhao, X. Wang, Y. Yin, W. Tian, G. Zeng, H. Li, S. Ye, L. Wu, J. Liu, Molecular Level Modulation of Anthraquinone-containing Resorcinol-formaldehyde Resin Photocatalysts for H_2O_2 Production with Exceeding 1.2 % Efficiency, Angew. Chem. Int. Ed., 62 (2023) e202218318.

[3] M. Kou, Y. Wang, Y. Xu, L. Ye, Y. Huang, B. Jia, H. Li, J. Ren, Y. Deng, J. Chen, Y. Zhou, K. Lei, L. Wang, W. Liu, H. Huang, T. Ma, Molecularly Engineered Covalent Organic Frameworks for Hydrogen Peroxide Photosynthesis, Angew. Chem. Int. Ed., 61 (2022) e202200413.

[4] C. Chu, Z. Chen, D. Yao, X. Liu, M. Cai, S. Mao, Large-Scale Continuous and In Situ Photosynthesis of Hydrogen Peroxide by Sulfur-Functionalized Polymer Catalyst for Water Treatment, Angew. Chem. Int. Ed. 63 (2024) e202317214.

[5] Y. Chen, R. Liu, Y. Guo, G. Wu, T.C. Sum, S.W. Yang, D. Jiang, Hierarchical assembly of donor–acceptor covalent organic frameworks for photosynthesis of hydrogen peroxide from water and air, Nat. Synth. 3 (2024) 998-1010.

[6] R. Liu, Y. Chen, H. Yu, M. Položij, Y. Guo, T.C. Sum, T. Heine, D. Jiang, Linkage-engineered donor–acceptor covalent organic frameworks for optimal photosynthesis of hydrogen peroxide from water and air, Nat. Catal. 7 (2024) 195-206.

[7] M. Gu, D.-Y. Lee, J. Mun, D. Kim, H.-i. Cho, B. Kim, W. Kim, G. Lee, B.-S. Kim, H.-i. Kim, Solar-to-hydrogen peroxide conversion of photocatalytic carbon dots with anthraquinone: Unveiling the dual role of surface functionalities, Appl. Catal., B, 312 (2022) 121379.

[8] Z. Li, X. Shi, H. Cheng, Y. Song, Y. Jiao, S. Shi, J. Gao, J. Hou, Atomically Dispersed Iron Active Sites on Covalent Organic Frameworks for Artificial Photosynthesis of Hydrogen Peroxide, Adv. Energy Mater. 14 (2024) 2302797.

[9] Z. Jin, S. Jin, X. Tang, W. Tan, D. Wang, S. Song, H. Zhang, T. Zeng, Rational Design of Conjugated Acetylenic Polymers Enables a Two-Electron Water Oxidation Pathway for Enhanced Photosynthetic Hydrogen Peroxide Generation, Small, 5 (2023) 2305004.

[10] Y. Zhang, C. Pan, G. Bian, J. Xu, Y. Dong, Y. Zhang, Y. Lou, W. Liu, Y. Zhu, H_2O_2 generation from O_2 and H_2O on a near-infrared absorbing porphyrin supramolecular photocatalyst, Nat. Energy, 8 (2023) 361-371.

[11] Y. Shiraishi, T. Takii, T. Hagi, S. Mori, Y. Kofuji, Y. Kitagawa, S. Tanaka, S. Ichikawa, T. Hirai, Resorcinol–formaldehyde resins as metal-free semiconductor photocatalysts for solar-to-hydrogen peroxide energy conversion, Nat. Mater., 18 (2019) 985-993.

[12] T. Liu, Z. Pan, K. Kato, J.J.M. Vequizo, R. Yanagi, X. Zheng, W. Yu, A. Yamakata, B. Chen, S. Hu, K. Katayama, C. Chu, A general interfacial-energetics-tuning strategy for enhanced artificial photosynthesis, Nat. Commun., 13 (2022) 7783.

[13] Y. Hou, P. Zhou, F. Liu, Y. Lu, H. Tan, Z. Li, M. Tong, J. Ni, Efficient Photosynthesis of Hydrogen Peroxide by Cyano-Containing Covalent Organic Frameworks from Water, Air and Sunlight, Angew. Chem. Int. Ed. 136 (2024) e202318562.

[14] X. Zhang, H. Su, P. Cui, Y. Cao, Z. Teng, Q. Zhang, Y. Wang, Y. Feng, R. Feng, J. Hou, X. Zhou, P. Ma, H. Hu, K. Wang, C. Wang, L. Gan, Y. Zhao, Q. Liu, T. Zhang, K. Zheng, Developing Ni single-atom sites in carbon nitride for efficient photocatalytic H_2O_2 production, Nat. Commun., 14 (2023) 7115.

[15] J.-N. Chang, J.-W. Shi, Q. Li, S. Li, Y.-R. Wang, Y. Chen, F. Yu, S.-L. Li, Y.-Q. Lan, Regulation of Redox Molecular Junctions in Covalent Organic Frameworks for H_2O_2 Photosynthesis Coupled with Biomass Valorization, Angew. Chem. Inter. Ed. 62 (2023) e202303606.

[16] H. Peng, H. Yang, J. Han, X. Liu, D. Su, T. Yang, S. Liu, C.-W. Pao, Z. Hu, Q. Zhang, Y. Xu, H. Geng, X. Huang, Defective ZnIn2S4 Nanosheets for Visible-Light and Sacrificial-Agent-Free H_2O_2 Photosynthesis via O_2/H_2O Redox, J. Am. Chem. Soc. 145 (2023) 27757-27766.

[17] Y. Huang, M. Shen, H. Yan, Y. He, J. Xu, F. Zhu, X. Yang, Y.-X. Ye, G. Ouyang, Achieving a solar-to-chemical efficiency of 3.6% in ambient conditions by inhibiting interlayer charges transport, Nat. Commun. 15 (2024) 5406.

[18] H. Tan, P. Zhou, M. Liu, Q. Zhang, F. Liu, H. Guo, Y. Zhou, Y. Chen, L. Zeng, L. Gu, Z. Zheng, M. Tong, S. Guo, Photocatalysis of water into hydrogen peroxide over an atomic Ga-N5 site, Nat. Synth. 2 (2023) 557-563.

[19] C. Feng, J. Luo, C. Chen, S. Zuo, Y. Ren, Z.-P. Wu, M. Hu, S. Ould-Chikh, J. Ruiz-Martínez, Y. Han, H. Zhang, Cooperative tungsten centers in polymeric carbon nitride for efficient overall photosynthesis of hydrogen peroxide, Energy Environ. Sci. 17 (2024) 1520-1530.

[20] Y. Deng, W. Liu, R. Xu, R. Gao, N. Huang, Y. Zheng, Y. Huang, H. Li, X.Y. Kong, L. Ye, Reduction of Superoxide Radical Intermediate by Polydopamine for Efficient Hydrogen Peroxide Photosynthesis, Angew. Chem. Inter. Ed. 63 (2024) e202319216.
