# Supplementary material for: Oxygen-oxygen bond cleavage enables efficient photocatalytic H2O2 production via an *O2 dissociation pathway
Source: Nat Commun. 2026 Jun 5;17:7218. doi: 10.1038/s41467-026-73685-x (PMC13396225; doi:10.1038/s41467-026-73685-x)
Supplement: Supplementary file 2 — Descriptions of Additional Supplementary Files [file 41467_2026_73685_MOESM2_ESM.pdf]

Description of Additional Supplementary Files

File Name: Supplementary Data 1

Description: The atomic coordinates of the optimized unit cells for the DFT-calculated of CN, CN-K, CN-Cs and CN-KCs; The adsorption configuration of O_2 at N sites on CN, CN-K, CN-Cs and CN-KCs; The adsorption configuration of O2 at different sites on CN-KCs; CN-KCs -O_2-site 1, CN-KCs -O_2-site 2, CN-KCs -O_2-site 3, CN-KCs-O_2-site 4; The adsorption configuration of H_2O at different sites on CN-KCs; CN-KCs- H_2O -site 1, CN-KCs - H_2O -site 2, CN-KCs - H_2O -site 3, CN-KCs- H_2O -site 4

File Name: Supplementary Data 2

Description: The geometries of ORR to H_2O_2 intermediates on CN, CN-K, CN-Cs and CN-KCs: CN+*O_2, CN-*O_2, CN-*O, CN-*OOH, CN-*HOOH, CN+*H_2O_2; CN-K+*O_2, CN-K-*O_2, CN-K-*O, CN-K-*OOH, CN-K-*HOOH, CN-K+*H_2O_2; CN-Cs+*O_2, CN-Cs-*O, CN-Cs-*O_2, CN-Cs-*OOH, CN-Cs-*HOOH, CN-Cs+*H_2O_2; CN-KCs+*O_2, CN-KCs-*O, CN-KCs-*O_2, CN-KCs-*OOH, CN-KCs-*HOOH, CN-KCs+*H_2O_2;
