# Supplementary material for: Oxygen-oxygen bond cleavage enables efficient photocatalytic H2O2 production via an *O2 dissociation pathway
Source: Nat Commun. 2026 Jun 5;17:7218. doi: 10.1038/s41467-026-73685-x (PMC13396225; doi:10.1038/s41467-026-73685-x)
Supplement: Supplementary file 5 — Transparent Peer Review file [file 41467_2026_73685_MOESM5_ESM.pdf]

nature portfolio

# Peer Review File

# Oxygen-oxygen bond cleavage enables efficient photocatalytic H2O2 production via an *O2 dissociation pathway

Corresponding Author: Professor Fuxian Wang

**This file contains all reviewer reports in order by version, followed by all author rebuttals in order by version.**

Version 0:

Reviewer comments:

Reviewer #1

(Remarks to the Author)
The manuscript describes the modification of a carbon nitride photocatalyst (with ordinary partial crystalline structure) with Cs, as done before for many heterogeneous catalysts. Not described before, the photocatalyst however developed at 96 -98 % AQY towards the reduction of O2 to H2O2, which is a sensation as such !
it indeed means that charge separation even in disordered carbon nitrides can be close to perfect, while activation and reaction direction of the substrate can be adjusted by the type of cation. This is by far more general than "only" perfect H2O2 synthesis and let us expect many other grand challende reactions, for instance photochemical Fischer-Tropsch reaction.
By isotope experiemnts, the authors demonstrate that an inverse isotope effect is active, i.e. the water is not first dissociated, but rather coactivated in the pores of PHI and reacts electrocyclically after electron transfer to O2. This is in nice agreement with a recent Nat.Comm paper, where the role of water in photochemical water splitting was analyzed by ambient pressure XPS, that is the picture continues to sharpen.
There are to m< opinion only two changes requested to improve this (already outstanding) manuscript before it could be accepted for publication:
1) Abstract and introduction still talk about classical proton transfer, while the own discussion at the end makes this mechanism improbable. I guess the the real secret is the so-called Cesium-effect, regularily discussed in heterogeneous catalysis where the Cs acts as some type of Lewis acid, binding and activating the O2. This "semantic confusion" even enters the title, where they talk about O2-dissociation mechanism, while in fact O2 never separates. it is the third electron of reduction which is always splitting the O2, while Carbon nitrides even without Cs only support 2 electron reduction.
I am only referee, but if this would be my paper, I would describe it as an FLP like activation enabling direct hydrogen transfer from the water.
2) The white spots in the HR-STEM are not localized Cesium atoms (for that, they are too triangular and too big), but a time average over a moving Cs atom, exploring the triangular pore (Debye Waller factor). Such frustrated positions are of course needed for FLP catalysis.

Reviewer #2

(Remarks to the Author)
In this manuscript, the authors design a K and Cs co-modified polymeric carbon nitride (CN-KCs) to fulfill photocatalytic H2O2 production from O2 reduction accompanied with ethylene glycol as hole scavenger. An exceptional photocatalytic H2O2 yield of 1806.6 μmol h-1 is achieved over CN-KCs, with a quantum efficiency of 95.4% at 420 nm and a solar-to-H2O2 conversion efficiency of 6.1%. Besides, a new *O2 dissociation pathway towards photocatalytic H2O2 synthesis is proposed. However, some of the data and the characterization results are vague, improper, insufficient, and questionable, which make this work suspectable. So, I don't recommend its publication in this journal, which is of high standards. Here are some of the problems in the manuscript:
1. The unit of the yield is very likely to be incorrect. In line 610-615 and Supplementary Fig.4, the authors claim that the reaction supernatant corresponds to a 10-fold dilution for the quantification of the concentration of $H_2O_2$ products. According to the standard fitting curve of H2O2 concentration using N, N-diethyl-p-phenylenediamine (DPD) method, the max concentration of H2O2, which can be directly acquired, is about 4000 μM. However, the H2O2 yield of 1806.6 μmol achieved over CN-KCs in 60 min is converted to 90330 μM, which is far beyond the measurable range. Thus, I think the authors

confused the meaning of the units (μmol and μM) and the real $H_2O_2$ yield is 1806.6 μM or 36.132 μmol. The corresponding performance, quantum efficiency, and solar-to-$H_2O_2$ conversion efficiency should be divided by 50 (the volume of the rection solution is 20 mL). These mistakes significantly reduce the importance of this work.
2. If the unit of the yield is right, the degradation rate of $H_2O_2$ of the obtained photocatalysts should use the 90 mM concentration of $H_2O_2$ rather than 5mM in Supplementary Fig. 15.
3. Line 131-132: "more than 120 L of $H_2O_2$ solution with a concentration of about 1100 μmol, demonstrating excellent performance stability and scalability for $H_2O_2$ production". Why is the unit of concentration used as μmol instead of μM? This is the evidence of the incorrect performance, quantum efficiency, and solar-to-$H_2O_2$ conversion efficiency.
4. According to the theoretical model proposed by the authors in Supplementary Fig. 56, the distance between Cs and K is more than 0.2 nm shown in Supplementary Fig. 29.
5. In Supplementary Fig. 12, two different non-protic solvents are used instead of $H_2O$ and the reaction is largely ineffective. Different protic solvents should also be used instead of $H_2O$ to study the role of $H_2O$ in $H_2O_2$ generation.
6. The fs-TA measurements are probably performed in air, where the Air/$O_2$ may affect the dynamic of the charge carriers. So, the additional fs-TA should be performed in the inert atmosphere to exclude the influence of Air/$O_2$ for comparation.
7. I don't know how $\tau_{avg}$ is attained smaller than both $\tau_1$ and $\tau_2$ in Supplementary Fig.44. The authors should give an explanation.
8. In line 232-234, the authors claim that "this inverse kinetic isotope effect on CN-Cs and CN-KCs suggests that $H_2O$ molecules do not necessarily undergo the traditional O-H dissociation pathway, but directly couple with oxygen-containing intermediates like *O to produce $H_2O_2$ (*O + *$H_2O$ → $H_2O_2$)". Maybe, the competing adsorption between *OH/*OD and *$O_2$ likely plays an instrumental role in the ORR just like the work in Ref 31 in the manuscript.
9. The use of triethylamine instead of EG makes a 1.28 folds enhancement of $H_2O_2$ production (Fig. 3i). If calculation of QE in the use of triethylamine, will the QE reach to 100%?
10. If the $H_2O_2$ production is from *O + *$H_2O$ → $H_2O_2$, the use of $^{18}O_2$+$H_2{}^{16}O$ and $^{16}O_2$+$H_2{}^{18}O$ will produce $H_2{}^{18}O^{16}O$. This product $H_2{}^{18}O^{16}O$ will decompose to 1/4 $^{16}O_2$, 1/2 $^{18}O^{16}O$, and 1/4 $^{18}O_2$ in the presence of $MnO_2$. This means that the peak at m/z of 36 in Fig. 4b-c shouldn't be the highest peak.
11. Furthermore, the results of $H_2O_2$ direct detection via GC-MS in Supplementary Fig. 49-51 are strange, too. The dominant peak at m/z of 36 means the major production of $H_2{}^{18}O^{18}O$. But, the use of $^{18}O_2$+$H_2{}^{16}O$ and $^{16}O_2$+$H_2{}^{18}O$ only produces $H_2{}^{18}O^{16}O$. The authors should explain the results.
12. The in situ ATR-FTIR experiments are proceeded in the presence of 0.5% EG (line 694). So, during the reaction, the appearance of the oxidized EG signal is inevitable. How to distinguish the signal of oxygen species from the oxidized EG?
13. The $O_2$ was replaced with isotopically labeled $^{18}O_2$ to acquire the ATR-FTIR spectra with the range of 790-690 $cm^{-1}$ in Supplementary Fig. 54. The spectra with the range of 1800-690 should be provided to conduct a more thorough study of the oxygen species signals.
14. For in situ XPS experiments, the control experiment under $O_2$, $H_2O$ and $O_2$+$H_2O$ in the dark should be provided to study whether the electron transfer caused by adsorption or by redox reaction.
15. In line 371-372, the authors claim that "In the XPS K 2p spectra, no significant changes in the K 2p peak position are observed under different conditions, indicating that excited carriers do not migrate onto K species". While, in my opinion, the binding energy shift of K 2p is evident just like Cs 3d. The phenomenon should be discussed.
16. If the catalysts restore to its original state after each round reaction, the free energy of *+$H_2O_2$ should be identical (J. Am. Chem. Soc. 2025, 147, 38, 34681-34689; Nat. Catal., 2024, 7(2): 195-206; Nat. Commun., 2025, 16(1): 6943.), which is differ from the results in Fig.6d-f. Why?
17. The transition states between some reaction coordinates (*$O_2$ and *O, *O and *$H_2O_2$), which are key to $O_2$ dissociation pathway and seem hard to proceed, should be calculated and provided for the convincing comparation of energy barrier.

Reviewer #3

(Remarks to the Author)
The manuscript by Liu and coauthors reported a new reaction pathway for photocatalytic $H_2O_2$ production via *$O_2$ dissociation process (*$O_2$ → 2*O), which was achieved by using K and Cs co-modified polymeric carbon nitride (CN-KCs). The CN-KCs was synthesized by modifying polymeric CN via a straightforward hydrothermal method with the presence of KCl and CsCl at high temperature. The resulting CN-KCs exhibited high photocatalytic performance, attaining a $H_2O_2$ production rate of 1806.6 μmol $h^{-1}$, a quantum efficiency of 95.4% at 420 nm, and a benchmark solar-to-$H_2O_2$ conversion efficiency of 6.1%. In general, modifying PCN for preparing photocatalysts is not new and has been reported elsewhere (International Journal of Hydrogen Energy, 2021, 46(77), 38310-38318, Journal of Hazardous Materials, 2022, 424, 127292, Dalton Transactions, 2022, 51(43), 16527-16535, International Journal of Hydrogen Energy, 2024, 51, 425-435). The reported procedures for CN-KCs synthesis based on hydrothermal method have no synthetic challenge. The structural design for CN-KCs is not clear as they did not clarify the advantages and purpose of utilization of K and Cs metals in this photocatalyst. The proposed new reaction pathway via *$O_2$ dissociation process (*$O_2$ → 2*O) is doubtful, particularly considering the involvement of dissociation of two strong chemical bonds. The theoretical calculations for $H_2O_2$ production are not completed and lack critical step for the formation of $H_2O_2$, so the conclusion made based on these results is too premature. In addition, the manuscript deserves a deeper interpretation of the data and a more detailed discussion in my opinion, particularly for the mechanism of the new process (*O + *$H_2O$ → $H_2O_2$). Considering the limited novelty and unclear scientific points, I cannot recommend its publication in Nature Communications.

Some detailed points:
1. The authors assumed that increasing the $O_2$ adsorption is possible to reduce the energy of *$O_2$ dissociation and cleavage of O=O bond, but the mechanism is unclear. Why will the dissociation energy decrease? The two processes seem unrelated.
2. The proposed new pathway for photocatalytic $H_2O_2$ production is doubtful, as this process requires the cleavage of two

strong covalent bonds, both O=O and H-O-H, to allow the intermediate *O react with H2O to produce H2O2 (H-O-O-H). Note that the cleavage of the H-O bond in H2O is an inevitable process for H2O2 photocatalysis and is also involved in the proposed new pathway.
3. The bond energy of O2 (O=O) is 498 kJ/mol, while the bond energy of O-H in H2O is 463 kJ/mol, so *O2 dissociation process (*O2 → 2*O) requires higher energy input than the cleavage of H-O. The new pathway is an energy-unfavorable process.
4. The contents of K and Cs in the CN-KCs are not known. How the content affecting the photocatalytic performance is unclear.
5. In Fig. 6e-g, H2O2 production based on *O + *H2O → H2O2 is not completed as the intermediate *O needs to be inserted into H-O-H to form new chemical bond (H-O-O-H). The dissociation of H-O-H and the reaction of *O and *O-H should be included in the theoretical calculations. One-step formation of H2O2 via*O + *H2O → H2O2 is impossible.
6. Lines 186-188: the author supposed that the Cs donates electron in CN-KCs, while this is difficult to understand as the cationic Cs+ ion used in preparation cannot provide electron anymore.
7. TA results showed that CN-KCs possessed the shortest lifetime for charge separated states, which is unfavoured for the utilization of photoinduced electrons and holes.
8. Lines 163-164: the authors indicated that K ions are located within the nitride pots among the three tri-s-triazine units of CN, but K does not possess the d orbitals to coordinate with N.

Reviewer #4

(Remarks to the Author)
This paper reports very impressive efficiencies for H2O2 production in on Cs loaded carbon nitride photocatalysts in the presence of glycol as electron source. The quantum efficiencies and stability are both impressive and suitable for publication in nature communications. I note the authors also also claim very high solar to chemical conversion (SCC) efficiencies. It is not clear to me how these are calculated, and in particular how the change in energy of the glycol to products reaction is included (which it must be if quoting a SCC efficiency). It is not clear to me SCC is a useful assay for such reactions.

The paper includes a extensive characterisation and analysis. This is the weaker side of the paper, in my opinion. While some analyses give insight into the reaction mechanism, and are interesting, others appear to me to give little clear insight, with conclusions not clearly justified by the results. For example, the fs-TAS results essentially show that adding Cs, the optical signal decays faster. This could potentially be due to electron transfer to Cs, but there is no direct evidence of this. There is also, I think, no evidence for a change in electron trapping. The description of the conclusions from these results should be revised to be limited to those which can be clearly concluded from the data.

The authors end their paper with some reaction mechanisms, which appear to be based on theory calculations. it is unclear how the experimental data supports, or indeed may question, these proposed mechanisms - this discussion needs to be added. In particular, it should be clarified whether the KIE, FTIR and XPS data support these mechanisms. It is not necessary that everything agrees - indeed this is a complex system where I would expect the interpretation of the results to often be quite ambiguous. But it is important for readers to be guided more clearly on what can be clearly concluded from the results, and what is suggested but not proven.

Overall I think the results in this manuscript are suitable for publication in nature communications. However the manuscript does need significant revisions, primarily in the discussion and conclusions which can be drawn from the analysis results.

Version 1:

Reviewer comments:

Reviewer #1

(Remarks to the Author)
I went through the answers to the referees and the altered manuscript, and my concerns were completely covered. I think that the manuscript has remarkably improved, and I can support publication in the present shape.

Reviewer #2

(Remarks to the Author)
The authors didn’t respond properly to some key comments. Judging from response 1, the data in the manuscript lacks credibility. Moreover, the mass results cannot match the proposed O2 dissociation mechanism. So, I do not recommend this manuscript for publication. Below are the detailed comments:
1. Regarding response 1: The authors stated that “In Supplementary Fig. 4b, the y-axis label should read 'μmol instead of 'μM.' The H2O2 yield of 1806.6 μmol reported in the main text for CN-KCs is correct.” in response 1. However, the colorimetric DPD method for detecting H2O2 has a significant flaw, that when H2O2 concentration exceeds a certain value, DPD·+ with typical absorption in 551 nm will be overoxidized and the absorbance will decrease significantly (Chemosphere, 2019, 224, 646-652.). I think the authors know the phenomenon, as the detection of reaction supernatant is after a 10-fold dilution. However, the claimed correct unit “μmol” results in the concentrations of H2O2 far exceeding the detectable limit.

The acquisition of updated standard fitting curve of $H_2O_2$ concentration is impossible. The relationship between the absorbance of DPD·+ and the concentration of $H_2O_2$ can refer to the references (Small, 2018, 14(9): 1703142; Chemosphere, 2019, 224, 646-652; Angewandte Chemie International Edition, 2022, 61, e202200413.). Furthermore, there is no need for 10-fold dilution of reaction supernatant, if the "μmol" is the correct unit. 400 μmol $H_2O_2$ in 1 mL solution to be tested corresponds to 8000 μmol $H_2O_2$ in reactor (total volume is 20mL), which is larger than the so-called $H_2O_2$ yield of 1806.6 μmol. Given that the author insists "μmol" is the correct unit, the rest of data is deemed lack of reliability. Also, the real production of $H_2O_2$ significantly reduce the importance of this work.
2. Regarding response 2 and 3: Please refer to comment 1.
3. Regarding response 6: If the fs-TA measurements are conducted in Ar, the shorter lifetime in CN-KCs is unfavored for the subsequent reaction, which I and reviewer 3 both deemed. The authors gave the explanation that "The reduced lifetime induced by Cs is likely due to the formation of new adsorption centers at Cs sites, which attract photogenerated carriers toward the Cs-associated regions. In the presence of $O_2$, these carriers can be rapidly transferred to the adsorbed $O_2$ molecules, thereby facilitating strong $O_2$ adsorption and activation." However, under light and UHV, there is no change in the binding energy of Cs 3d (Fig 3f), indicating no photoelectron transfers to Cs, which is contradictory to the given explanation. Furthermore, the viewpoint that carriers can be rapidly transferred to the adsorbed $O_2$ is lack of evidence. The fsTA measurements in the presence of $O_2$ are essential for comparison with those performed in Ar.
4. Regarding response 9: In Fig 3i, the use of triethylamine instead of EG makes a 1.28 folds enhancement of $H_2O_2$ production. Whereas, in QE calculation, the $H_2O_2$ production in the presence of TEA only gives only a 1.03 folds enhancement compared to EG. Why?
5. As the authors claimed that only 35-50% of the $H_2O_2$ is generated via *O + *$H_2O$ pathway and 50-65% of the $H_2O_2$ is generated via PCET, the KIE value for CN-KCs smaller than 1 is unreasonable.
6. Regarding response 10: First, I don't understand what is the basis for the two key premised in response 10. Second, in the case of *16O coupled with $H_2{}^{18}O$, the two premises is contradictory to each other. Third, based on the conclusion that 50-65% of the $H_2O_2$ is generated via PCET ($^{16}O_2$ + 2e- + 2H+ → $H_2{}^{16}O_2$), the highest peak should be the peak at m/z of 32 (the condition: $^{16}O_2$+ $H_2{}^{18}O$), which is contradictory to the results in Fig 4b and c. Forth, combined with the viewpoint that "when *16O is coupled with $H_2{}^{18}O$, the 16O atom dissociates more readily than 18O, the highest peak should rather be the peak at m/z of 32 than m/z of 36. Fifth, due to the smaller difference in atom weight of 16O and 18O, the KIE is very close to 1. So, the dissociation rate difference between 16O and 18O is close.
7. Regarding response 11: Please refer to comment 6.
8. Regarding response 13: The vibrations of oxygen species showed in Fig 4d-g should be also labeled in Fig R7 under 18O-labeled $O_2$ gas to prove the isotope effect.
9. Regarding response 16 and 17: The choice of reference energy shouldn't lead to the massive energy difference of "*+$H_2O_2$". The DFT calculation should be re-performed to give more reliable results.

Reviewer #3

(Remarks to the Author)
The revised manuscript by Liu and co-authors has been improved with substantial supplementary experiments added, which indeed are important to help understand the new pathway of cleavage of O–O for photocatalytic $H_2O_2$ generation. Nonetheless, I still have some concerns on the mechanism part as well as the explanations of experimental data.

1. For the isotopic experiments, I found that this experiment cannot give any insights in the reaction pathway due to the co-existence of $^{18}O_2$, $^{16}O_2$ and 18O-16O. Particularly, these oxygen species do not have any specific ratio, making the analysis of the results difficult not to mention to explain the reaction mechanism. Note that if the experiment cannot prove the mechanism or provide the solid evidence for the reaction pathway, it is hence not self-consistent and convincing. The author explained this with several possibilities, but it didn't really solve the problem, particularly considering their explanation made based on several hypotheses.
2. In-situ FTIR showed the signals of *O, which are very weak with low S/N ratio to distinguish them. Because this signal is key evidence to identify the reaction mechanism, I wish they can reconsider the experimental procedures and redo the measurement to provide the observable signals at least.
3. Another must be mentioned is the processing procedure of isotope labelling experiment. How to remove ethylene glycol and its derivatives from the reaction system is unknown. The author did not provide any experimental details. Note that these components are likely to have signals in mass spectrum but absent in their results. These derivatives are hydrophilic and mix up with water, fully removing these compounds from water is impossible.
4. Supplementary Fig. 7 showed the NMR spectrum of the oxidative product from EG oxidation, but no product peaks were identified. Please assign the characteristic peaks from acid, aldehyde, etc.
5. The performance comparison with reported literature was presented in Fig 1g but was unfair. The authors utilized sacrificial reagents in both QE and SCC measurements, which did not reflect the real superiority of their photocatalyst. The testing conditions should be identical with these literatures.
6. Another bug is the QE calculation based on the 2e– ORR pathway that did not occur in this reaction. The hypothesis of two electrons transfer is not suitable as the variation of chemical valence in O of $H_2O$ is one. The QE calculation was therefore not correct. Additionally, the experimental parameters including light intensity, distance, irradiation area should be provided.

Reviewer #4

(Remarks to the Author)

The authors have signficantly largely addressed my concerns with the manuscript. I only have two remaining concerns:
1) in the abstract, it should be clearly stated that the SCC $H_2O_2$ efficiency is obtained in the presence of a sacrificial electron donor
2) in the discussion of the uf data, the statement 'density of shallow trap states is clearly enhanced' is not, as far as I can see, clear from the data and should be deleted.

Version 2:

Reviewer comments:

Reviewer #2

(Remarks to the Author)
I have no further questions.

Reviewer #3

(Remarks to the Author)
The authors have done a lot of improvements. I am happy to recommend its acceptance. Congratulations!

Reviewer #4

(Remarks to the Author)
Revisions now address now concerns fine

**Open Access** This Peer Review File is licensed under a Creative Commons Attribution 4.0 International License, which permits use, sharing, adaptation, distribution and reproduction in any medium or format, as long as you give appropriate credit to the original author(s) and the source, provide a link to the Creative Commons license, and indicate if changes were made.
In cases where reviewers are anonymous, credit should be given to 'Anonymous Referee' and the source.
The images or other third party material in this Peer Review File are included in the article's Creative Commons license, unless indicated otherwise in a credit line to the material. If material is not included in the article's Creative Commons license and your intended use is not permitted by statutory regulation or exceeds the permitted use, you will need to obtain permission directly from the copyright holder.
To view a copy of this license, visit https://creativecommons.org/licenses/by/4.0/

Dear Editor,

Thanks for the efficient processing and assessment of our manuscript (Manuscript number: NCOMMS-25-72870). After carefully reviewing the reasons for rejection, we found that the concerns raised by the reviewer are not fundamentally critical. In fact, several of the issues highlighted have already been addressed and discussed in detail in our manuscript. For the remaining points, we believe that we can provide clear and satisfactory responses to fully address the reviewer's questions. We therefore apply for an arbitration of our manuscript. We sincerely hope we could have the second chance to be evaluated and we expect insightful and constructive comments even if they are negative.

## Response to the reviewers' Comments

Many thanks to the reviewers for their valuable comments and suggestions. The followings are the point-by-point answers to the concerns:

**Reviewer 1**: The manuscript describes the modification of a carbon nitride photocatalyst (with ordinary partial crystalline structure) with Cs, as done before for many heterogeneous catalysts. Not described before, the photocatalyst however developed at 96 -98 % AQY towards the reduction of O2 to H2O2, which is a sensation as such !
it indeed means that charge separation even in disordered carbon nitrides can be close to perfect, while activation and reaction direction of the substrate can be adjusted by the type of cation. This is by far more general than "only" perfect H2O2 synthesis and let us expect many other grand challende reactions, for instance photochemical Fischer-Tropsch reaction.
By isotope experiemnts, the authors demonstrate that an inverse isotope effect is active, i.e. the water is not first dissociated, but rather coactivated in the pores of PHI and reacts electrocyclically after electron transfer to O2. This is in nice agreement with a recent Nat.Comm paper, where the role of water in photochemical water splitting was analyzed by ambient pressure XPS, that is the picture continues to sharpen.
There are to my opinion only two changes requested to improve this (already outstanding) manuscript before it could be accepted for publication:
**Q1.** Abstract and introduction still talk about classical proton transfer, while the own discussion at the end makes this mechanism improbable. I guess the the real secret is the so-called Cesium-effect, regularily discussed in heterogeneous catalysis where the Cs acts as some type of Lewis acid, binding and activating the O2. This "semantic confusion" even enters the title, where they talk about O2-dissociation mechanism, while in fact O2 never separates. it is the third electron of reduction which is always splitting the O2, while Carbon nitrides even without Cs only support 2 electron reduction.
I am only referee, but if this would be my paper, I would describe it as an FLP like activation enabling direct hydrogen transfer from the water.
**R1**：We sincerely appreciate the reviewer's thoughtful and insightful comments. The suggestion regarding the possible FLP-like activation mode and the role of Cs as a Lewis-acidic center is particularly constructive and indeed enriches the mechanistic understanding of our system.

We fully agree that the participation of Cs may go beyond a classical proton-transfer description, and that its interaction with $O_2$ can conceptually be related to the "Cs-effect" widely discussed in heterogeneous catalysis. We appreciate the reviewer pointing out the semantic ambiguity between "$O_2$ dissociation" and "O–O bond weakening under the third-electron reduction step", and this has helped us refine our mechanistic description significantly.

In the revised manuscript, we would highlight the the Cs–N interfacial pair can function analogously to a frustrated Lewis pair to facilitate hydrogen (proton/electron) transfer and the activation of $O_2$ is assisted by Lewis-acidic Cs sites.

We greatly appreciate the reviewer for raising this point, which indeed improves the mechanistic clarity and conceptual depth of our work.

**Q2.** The white spots in the HR-STEM are not localized Cesium atoms (for that, they are too triangular and too big), but a time average over a moving Cs atom, exploring the triangular pore (Debye Waller factor). Such frustrated positions are of course needed for FLP catalysis.
**R2**：We sincerely thank the reviewer for their comment, just acorrd with the R1, we could highlight the Cs–N interfacial pair can function analogously to a frustrated Lewis pair in the manuscript.

**Reviewer 2**: In this manuscript, the authors design a K and Cs co-modified polymeric carbon nitride (CN-KCs) to fulfill photocatalytic H2O2 production from O2 reduction accompanied with ethylene glycol as hole scavenger. An exceptional photocatalytic H2O2 yield of 1806.6 μmol h-1 is achieved over CN-KCs, with a quantum efficiency of 95.4% at 420 nm and a solar-to-H2O2 conversion efficiency of 6.1%. Besides, a new *O2 dissociation pathway towards photocatalytic H2O2 synthesis is proposed. However, some of the data and the characterization results are vague, improper, insufficient, and questionable, which make this work suspectable. So, I don't recommend its publication in this journal, which is of high standards.
**Response to comment (R)**: We sincerely thank the reviewer for the thorough evaluation of our manuscript and for the critical comments regarding the quality of the data and characterizations. We fully understand the reviewer's concerns about the clarity and reliability of several results, and we greatly appreciate these comments as they have helped us significantly improve the rigor and presentation of our work.
In the revised manuscript, we have carefully addressed all issues raised. These revisions collectively strengthen the evidence supporting the proposed photocatalytic mechanism and enhance the overall reliability of our conclusions.
We sincerely appreciate the reviewer's insightful comments, which have greatly improved the scientific quality of the manuscript. We hope that the revisions now meet the high standards of the journal.

Here are some of the problems in the manuscript:
**Q1.** The unit of the yield is very likely to be incorrect. In line 610-615 and Supplementary Fig.4, the authors claim that the reaction supernatant corresponds to a 10-fold dilution for the quantification of the concentration of H2O2 products. According to the standard fitting curve of H2O2 concentration using N, N-diethyl-p-phenylenediamine (DPD) method, the max concentration of H2O2, which can be directly acquired, is about 4000 μM. However, the H2O2 yield of 1806.6 μmol achieved over CN-KCs in 60 min is converted to 90330 μM, which is far beyond the measurable range. Thus, I think the authors confused the meaning of the units (μmol and μM) and the real $H_2O_2$ yield is 1806.6 μM or 36.132 μmol. The corresponding performance, quantum efficiency, and solar-to-$H_2O_2$ conversion efficiency should be divided by 50 (the volume of the rection solution is 20 mL). These mistakes significantly reduce the importance of this work.
**R1**：We sincerely appreciate the reviewer for pointing out the error. In Supplementary Fig. 4b. The y-axis label should read 'μmol instead of 'μM.' The $H_2O_2$ yield of 1806.6 μmol reported in the main text for CN-KCs is correct. We apologize for this careless mistake.

**Q2.** If the unit of the yield is right, the degradation rate of H2O2 of the obtained photocatalysts should use the 90 mM concentration of H2O2 rather than 5mM in Supplementary Fig. 15.

**R2**: We understand this comment to be a continuation of question Q1. The correction of the standard curve units (from μM to μmol) does not affect the described $H_2O_2$ yield of 1806.6 μmol. Therefore, we have updated Supplementary Fig. 15 to reflect the correct concentration of $H_2O_2$, changing '5 mM' to '5 mmol'.

**Q3.** Line 131-132: "more than 120 L of H2O2 solution with a concentration of about 1100 μmol, demonstrating excellent performance stability and scalability for H2O2 production". Why is the unit of concentration used as μmol instead of μM? This is the evidence of the incorrect performance, quantum efficiency, and solar-to-H2O2 conversion efficiency.

**R3**: This ultimately traces back to the unit discrepancy in Supplementary Fig. 4b, which should be corrected to 1100 μmol/L (or 1100 μM). We have carefully checked and confirmed that calculations of the quantum efficiency and solar-to-$H_2O_2$ conversion efficiency are correct.

**Q4.** According to the theoretical model proposed by the authors in Supplementary Fig. 56, the distance between Cs and K is more than 0.2 nm shown in Supplementary Fig. 29.

**R4**: We appreciate the reviewer's careful examination of the structural model. The apparent discrepancy between the Cs–K distance in Supplementary Fig. 29 and the DFT-optimized structure in Supplementary Fig. 56 can be rationalized from three aspects:

(1) **Intrinsic structural corrugation**. Experimental XRD shows that polymeric carbon nitride does not form a perfectly planar structure; instead, the nanosheets exhibit noticeable corrugation and local bending. Such structural distortion can effectively shorten the projected interatomic distance between Cs and K in STEM images.

(2) **Electron-beam incidence angle in HAADF-STEM**. The apparent distance in HAADF-STEM is strongly dependent on the imaging tilt angle. Our nanosheet sample is unlikely to be perfectly perpendicular to the electron beam. For instance, a real spacing of ~0.35 nm would appear significantly smaller when the sheet is imaged at a tilt (e.g., a 60° tilt would reduce the projected distance by about half). Thus, the measured distance in STEM can be smaller than the actual Cs–K separation.

(3) **Possible multilayer stacking.** We also cannot exclude the possibility of local multilayer stacking during STEM imaging, which may alter the projected atomic positions and further affect the measured Cs–K distance.

These combined factors may explain why the Cs–K distance observed in STEM images appears shorter than that in the DFT structural model.

**Q5.** In Supplementary Fig. 12, two different non-protic solvents are used instead of H2O and the reaction is largely ineffective. Different protic solvents should also be used instead of H2O to study the role of H2O in H2O2 generation.

**R5**: We appreciate your insightful suggestion. In response, we conducted experiments using methanol, ethanol, and formic acid as substitutes for $H_2O$, maintaining the 0.5% (v/v) EG concentration. The results, presented in Figure R1, demonstrate that $H_2O_2$ production is marginally reduced when employing methanol, ethanol, and formic acid compared to $H_2O$. Nevertheless, the activity remains substantially greater than that observed with non-protic solvents, confirming the importance of protons in the reaction mechanism. The enhanced activity observed with $H_2O$ further demonstrates that in our reaction system, it is not necessary to first deprotonate $H_2O$ before reacting with $O_2$. Instead, water can directly react with the dissociated *O species to produce $H_2O_2$.

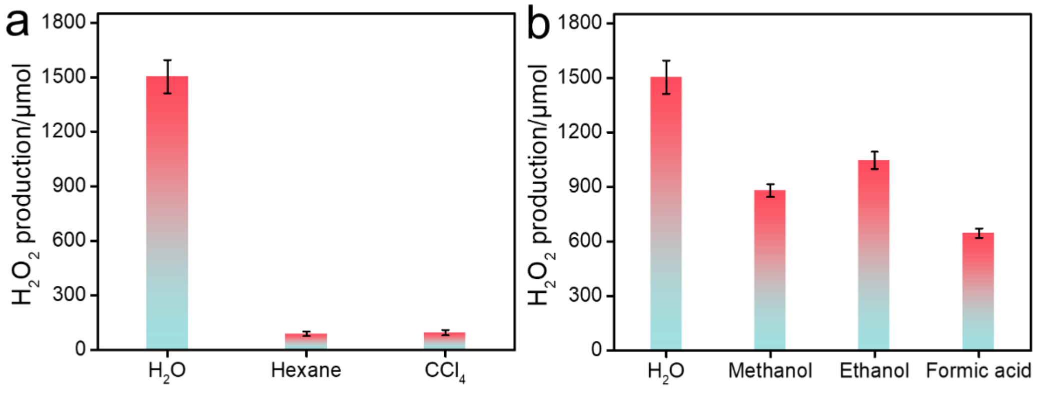


**Figure. R1.** $H_2O_2$ generation rates in (a) non-protic solvents and (b) protic solvents were used instead of $H_2O$ with a 0.5% (v/v) EG using CN-KCs.

**Q6.** The fs-TA measurements are probably performed in air, where the Air/O2 may affect the dynamic of the charge carriers. So, the additional fs-TA should be performed in the inert atmosphere to exclude the influence of Air/O2 for comparation.

**R6**: We thank the reviewer for this valuable suggestion. We would like to clarify that the current fs-TA measurements were indeed conducted under an Ar atmosphere, so the observed charge carrier dynamics are not influenced by ambient $O_2$.

**Q7.** I don't know how τavg is attained smaller than both τ1 and τ2 in Supplementary Fig.44. The authors should give an explanation.

**R7**: We thank the reviewer for pointing out this error. Indeed, the value of $\tau_{avg}$ reported in Supplementary Fig. 44 was mistakenly calculated as $\tau_{avg} = \tau_1 \cdot A_1 + \tau_2 \cdot A_2$ directly from the fitting parameters shown in Figure R2. Upon re-evaluation, we have recalculated $\tau_{avg}$ correctly, and the updated values are presented in Figure R3. We apologize for this oversight and have updated the Supplementary Information accordingly.

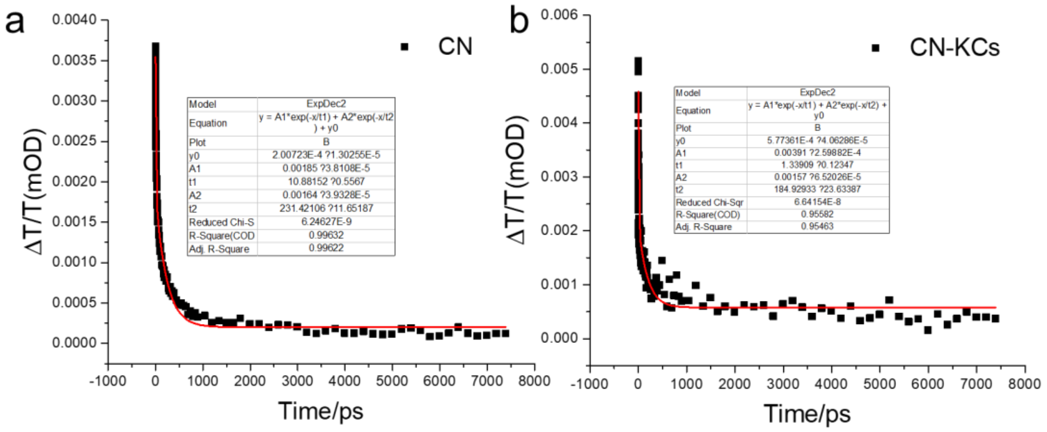


Figure R2. Bi-exponential fitting curves of the fs-TA decay spectra at 600 nm for CN and CN-KCs catalysts.

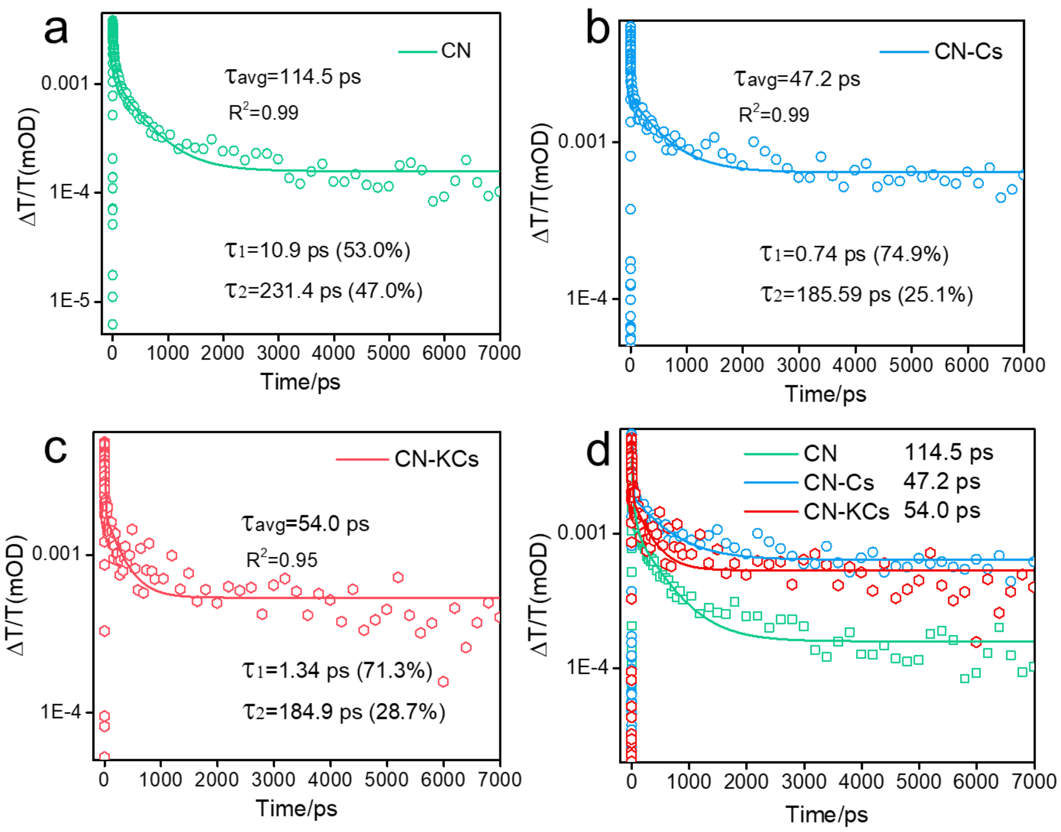


Figure R3. Bi-exponential fitting curves of the fs-TA decay spectra at 600 nm for CN, CN-Cs and CN-KCs catalyst.

**Q8.** In line 232-234, the authors claim that "this inverse kinetic isotope effect on CN-Cs and CN-KCs suggests that H2O molecules do not necessarily undergo the traditional O-H dissociation pathway, but directly couple with oxygen-containing intermediates like *O to produce H2O2 (*O + *H2O → H2O2)". Maybe, the competing adsorption between *OH/*OD and *O2 likely plays an instrumental role in the ORR just like the work in Ref 31 in the manuscript.

**R8**: We thank the reviewer for this very constructive comment. Indeed, on CN-Cs and CN-KCs, competitive adsorption between *$O_2$ and other intermediates can play an important role in the ORR, consistent with previous reports (Ref. 31). In our system, two parallel reaction pathways coexist on CN-KCs: (i) the conventional PCET pathway, in which *$O_2$ is hydrogenated to *OOH and finally to $H_2O_2$, and (ii) the newly proposed O–O dissociation pathway in the current work, in which *O directly couples with *$H_2O$ to form $H_2O_2$ (*O + *$H_2O$ → $H_2O_2$).

Based on a rough estimation using electron count in the final $H_2O_2$ product, we infer that approximately 35–50% of the $H_2O_2$ is generated via the *O + *$H_2O$ pathway, while the rest is still produced through the conventional PCET pathway. This indicates that both pathways contribute to $H_2O_2$ formation, with the newly identified dissociation pathway playing a significant role in the overall catalytic process.

**Q9.** The use of triethylamine instead of EG makes a 1.28 folds enhancement of H2O2 production (Fig. 3i). If calculation of QE in the use of triethylamine, will the QE reach to

100%?

**R9**：We thank the reviewer for this insightful question. Based on our calculations, when using triethylamine (TEA) instead of ethylene glycol (EG), the quantum efficiency (QE) at 420 nm can reach 98.2% (Figure R4).

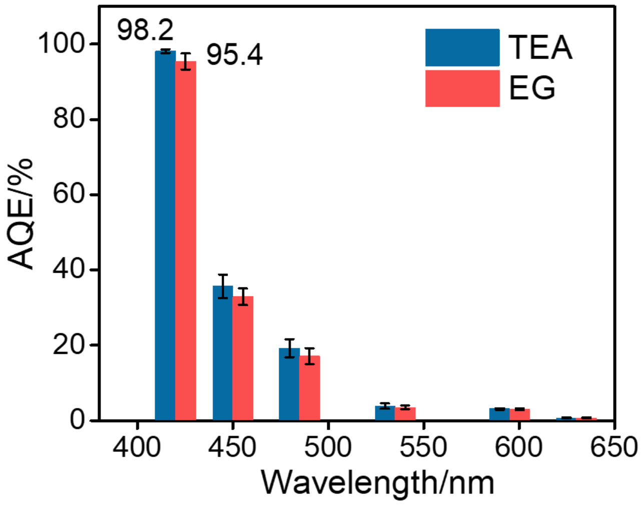


Figure R4. Apparent quantum efficiency (AQE) for $O_2$ reduction to $H_2O_2$ at different wavelengths using TEA or EG.

**Q10.** If the $H_2O_2$ production is from *O + *$H_2O$ → $H_2O_2$, the use of $^{18}O_2$+$H_2{}^{16}O$ and $^{16}O_2$+$H_2{}^{18}O$ will produce H218O16O. This product $H_2{}^{18}O^{16}O$ will decompose to 1/4 $^{16}O_2$, 1/2 $^{18}O^{16}O$, and 1/4 $^{18}O_2$ in the presence of MnO2. This means that the peak at m/z of 36 in Fig. 4b-c shouldn't be the highest peak.

**R10**：We thank the reviewer for this valuable comment. In our system, two parallel reaction pathways coexist on CN-KCs: (i) the conventional PCET pathway, in which *$O_2$ is hydrogenated to *OOH and finally to $H_2O_2$, and (ii) the newly proposed O–O dissociation pathway in the current work, in which *O directly couples with *$H_2O$ to form $H_2O_2$ (*O + *$H_2O$ → $H_2O_2$).

Based on a rough estimation using electron count in the final $H_2O_2$ product, we infer that approximately 35–50% of the $H_2O_2$ is generated via the *O + *$H_2O$ pathway, while the rest is still produced through the conventional PCET pathway. This indicates that both pathways contribute to $H_2O_2$ formation, with the newly identified dissociation pathway playing a significant role in the overall catalytic process.

And when referring to the new O–O dissociation pathway, we may first need to clarify two key premises. The first is that after the coupling of *O with $H_2O$ to form $H_2O_2$, the original *$H_2O$ intermediate is more stable. Consequently, the oxygen atom that is bound as an *O species onto *$H_2O$ is more likely to be released first and tends to form $O_2$ preferentially. The second premise is that in $H_2O_2$, the $^{18}O$ isotope atom is more prone to dissociation than $^{16}O$. Based on which, when *$^{18}O$ is coupled with $H_2{}^{16}O$ to form $H_2{}^{16}O^{18}O$, the $^{18}O$ atom is more likely to dissociate during decomposition, thus predominantly leading to the formation of $^{18}O_2$ (Figure R5). When *$^{16}O$ is coupled with $H_2{}^{18}O$, the $^{16}O$ atom dissociates

more readily than $^{18}O$, resulting in the presence of both mz=36 and 34 isotopic $O_2$ signals. As such, the peak at m/z of 36 would be more intensed.

In summary, under these conditions, the product isotope distribution cannot be predicted based on the new reaction pathway we proposed.

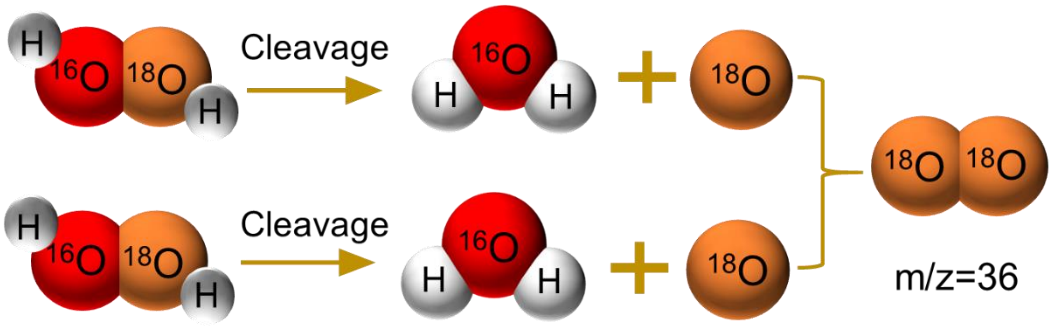


**Figure R5.** Cartoon schematic of the mass spectrum peak at m/z 36 for isotopically labeled H2$^{16}O^{18}O$.

**Q11.** Furthermore, the results of $H_2O_2$ direct detection via GC-MS in Supplementary Fig. 49-51 are strange, too. The dominant peak at m/z of 36 means the major production of $H_2{}^{18}O^{18}O$. But, the use of $^{18}O_2$+$H_2{}^{16}O$ and $^{16}O_2$+$H_2{}^{18}O$ only produces $H_2{}^{18}O^{16}O$. The authors should explain the results.

**R11**：We thank the reviewer for this comment. This question appears to be an extension of Q10, and the same explanation provided in R10 applies here. That is, the observed mass spectra are consistent with the exchange and labeling behavior discussed previously. We refer to our detailed discussion in R10 for clarification.

**Q12.** The in situ ATR-FTIR experiments are proceeded in the presence of 0.5% EG (line 694). So, during the reaction, the appearance of the oxidized EG signal is inevitable. How to distinguish the signal of oxygen species from the oxidized EG?

**R12**. This is a good point. Figure R6 presents the FTIR spectra of ethylene glycol (EG) and glycolaldehyde. As shown, the major bands of the two molecules are highly similar, with glycolaldehyde exhibiting only one new feature—the C=O stretching vibration of the aldehyde group. However, even this peak is difficult to distinguish in practice. During the in situ ATR-FTIR measurements, we performed repeated background scans to subtract the EG signals completely. As a result, any species with similar vibrational fingerprints, would also be largely removed from the spectra.

Moreover, in contrast to ORR intermediates (*OOH, *O-O, *O), which remain strongly adsorbed on the catalyst surface and therefore show strong ATR-FTIR signals, glycolaldehyde rapidly desorbs into the solution upon formation and becomes highly diluted. Its surface concentration is therefore extremely low, causing the corresponding FTIR signals to be quenched and essentially undetectable. As such, no feature of glycolaldehyde is observed in the ATR-FTIR spectra in Fig. 4.

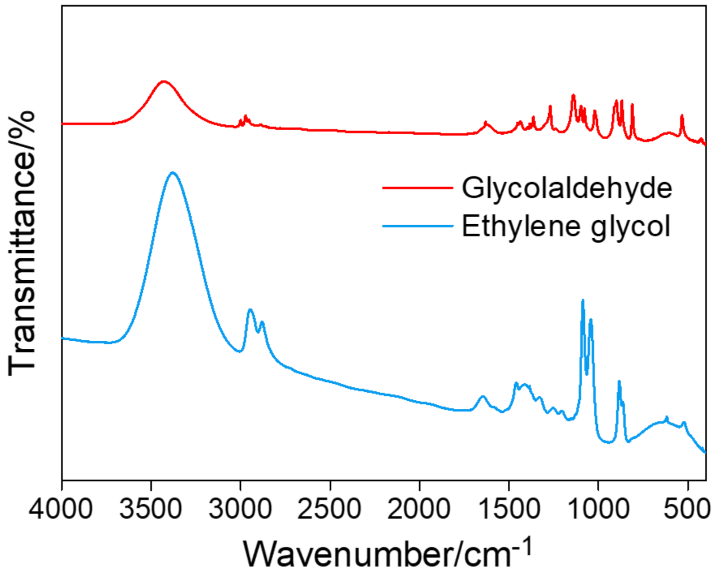


**Figure R6.** FTIR spectrum of ethylene glycol and glycolaldehyde sample.

**Q13.** The $O_2$ was replaced with isotopically labeled $^{18}O_2$ to acquire the ATR-FTIR spectra with the range of 790-690 cm-1 in Supplementary Fig. 54. The spectra with the range of 1800-690 should be provided to conduct a more thorough study of the oxygen species signals.

**R13：** According to the reviewer's suggestion, we have now presented the ATR-FTIR spectra in the range of 1800–690 $cm^{-1}$ in Figure R7.

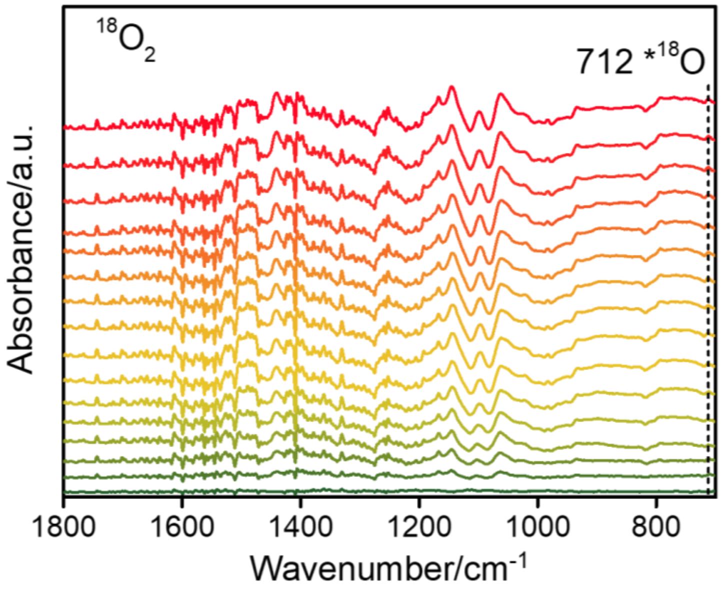


**Figure R7.** In situ ATR-FTIR experiments for CN-KCs under visible light illumination, conducted under 18O-labeled O2 gas. FTIR spectra were collected at 5-minute intervals.

**Q14.** For in situ XPS experiments, the control experiment under O2, H2O and O2+H2O in the dark should be provided to study whether the electron transfer caused by adsorption

or by redox reaction.

**R14**: We appreciate the reviewer's insightful suggestion. Following your advice, we have conducted the required control in situ XPS measurements under $O_2$, $H_2O$, and $O_2$ + $H_2O$ conditions in the dark, in order to distinguish whether the observed electron transfer originates from mere adsorption or from light-driven redox processes. The corresponding XPS spectra have now been added as Figure R8-R10 for comparison.

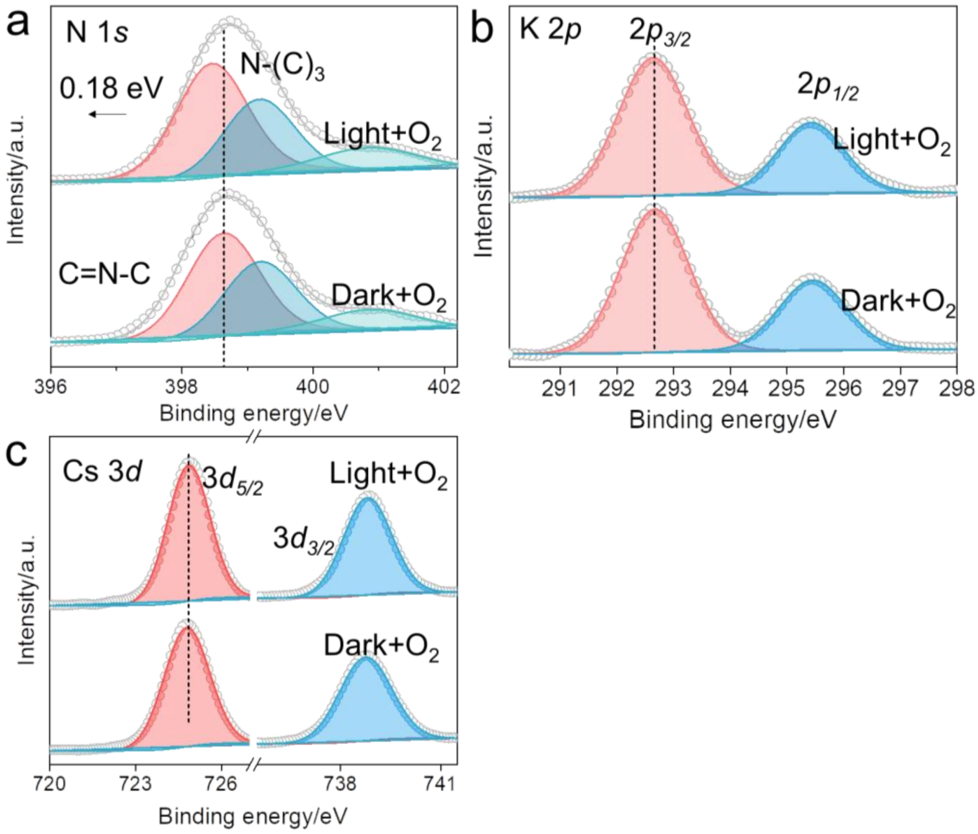


**Figure R8.** In situ XPS experiments of **a.** N 1s, **b.** K 2p and **c.** Cs 3d spectra under the O2 gas with dark time or illumination on CN-KCs.

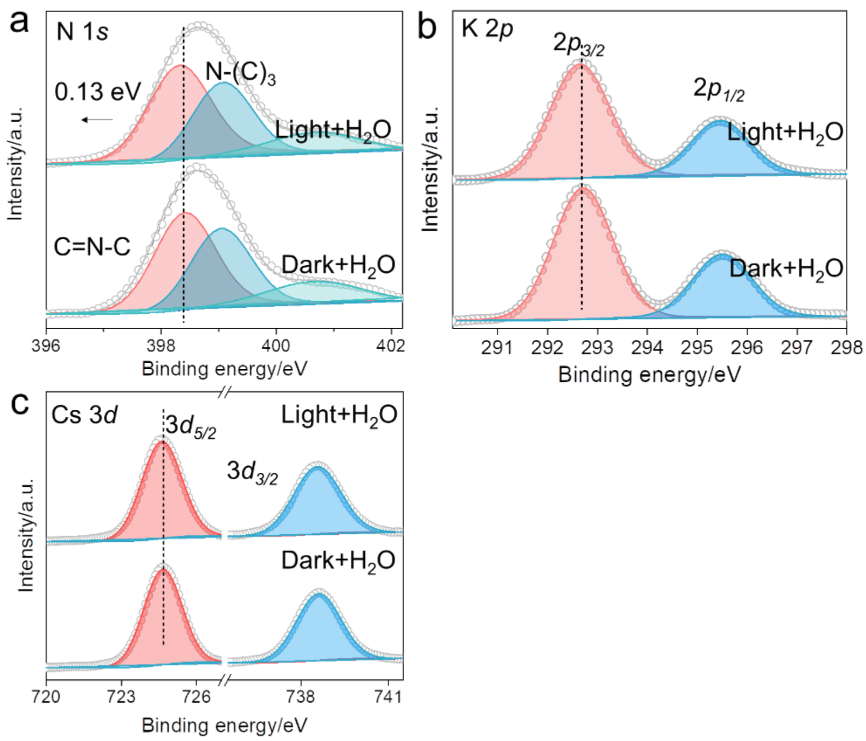


**Figure R9.** In situ XPS experiments of **a.** N 1s, **b.** K 2p and **c.** Cs 3d spectra under the $H_2O$ steam with dark time or illumination on CN-KCs.

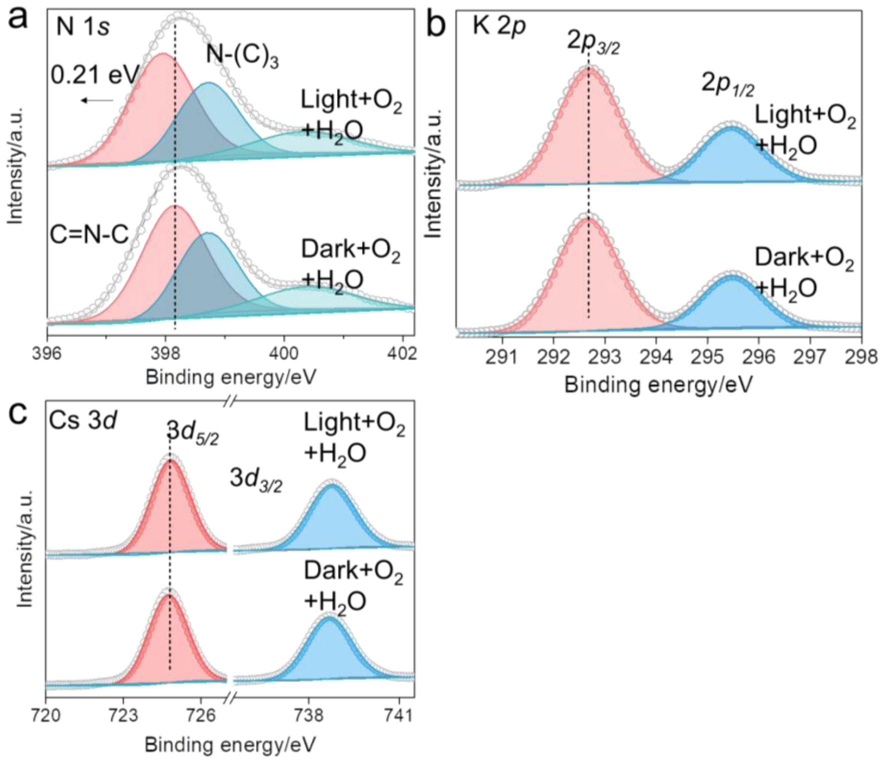


**Figure R10.** In situ XPS experiments of **a.** N 1s, **b.** K 2p and **c.** Cs 3d spectra under the O2+$H_2O$ with dark time or illumination on CN-KCs.

**Q15.** In line 371-372, the authors claim that "In the XPS K 2p spectra, no significant changes in the K 2p peak position are observed under different conditions, indicating that excited carriers do not migrate onto K species". While, in my opinion, the binding energy shift of K 2p is evident just like Cs 3d. The phenomenon should be discussed.

**R15**: We sincerely thank the reviewer for pointing this out. Our previous description was indeed ambiguous, and we have now revised the relevant content accordingly: In the XPS K 2p spectra, apart from the light illumination under ultra-high vacuum (UHV) conditions, no significant changes in the K 2p peak position are observed under different control conditions among under $O_2$, $H_2O$ and $O_2$+$H_2O$, indicating that both the $O_2$ and the $H_2O$ reactant are not directly absorbed onto K species. In contrast, a shift to higher binding energy of nearly 0.2 eV in the XPS Cs 3d spectra (Fig. 5f) is observed under light+$O_2$ and light+$O_2$+$H_2O$ conditions (724.9 eV), compared to the light+UHV condition (724.7 eV). This shift is not presented in XPS Cs 3d spectra, when introducing the $O_2$ or $O_2$+$H_2O$ mixture. This is due to the presence frustrated Lewis pair (FLP) of dual adsorption sites, Cs-$N_2$, for $O_2$ on CN-KCs.

**Q16.** If the catalysts restore to its original state after each round reaction, the free energy of *+H2O2 should be identical (J. Am. Chem. Soc. 2025, 147, 38, 34681-34689; Nat. Catal., 2024, 7(2): 195-206; Nat. Commun., 2025, 16(1): 6943.), which is differ from the results in Fig.6d-f. Why?

**R16**: We sincerely thank the reviewer for pointing out the important thermodynamic principle that, if the catalyst fully returns to its initial state after each catalytic cycle, the free energy of the "* + $H_2O_2$" final state should indeed be identical across different reaction pathways when referenced to the same initial state, as demonstrated in the cited works (JACS 2025, 147, 34681; Nat. Catal. 2024, 7, 195; Nat. Commun. 2025, 16, 6943).

We would like to clarify that the discrepancy in our calculated free energy of "* + $H_2O_2$"" does not originate from an intrinsic mechanistic inconsistency. Instead, it results from a difference in the **choice of reference energy (zero-point)** during data processing. In our current presentation, different pathways were referenced to slightly different "initial states," leading to different numerical values for the final "* + $H_2O_2$"" state, even though the catalyst is indeed restored to its original configuration.

If all pathways are referenced to a **consistent initial state**—for example, clean surface + gas-phase $H_2O_2$" or clean surface + gas-phase $O_2$ + $H_2O$—we can readily make all "* + $H_2O_2$" free energies identical, exactly as the reviewer pointed out. This does not require re-optimizing structures, only re-normalizing the thermodynamic cycle. Importantly, this adjustment does **not** affect the relative energy barriers, rate-determining steps, nor the qualitative trends that justify the proposed mechanism; it only ensures thermodynamic self-consistency across pathways.

**Q17.** The transition states between some reaction coordinates (*$O_2$ and *O, *O and *$H_2O_2$), which are key to $O_2$ dissociation pathway and seem hard to proceed, should be calculated and provided for the convincing comparation of energy barrier.

**R17**: We thank the reviewer for this valuable suggestion. The reaction coordinates used in our theoretical calculations were derived from the intermediates detected in the ATR-FTIR spectra. Our DFT calculations were thus performed based on the experimentally observed species. Moving forward, we plan to develop more advanced time-resolved ultrafast spectroscopic techniques to capture additional transient intermediates. Once these intermediates are identified experimentally, we will further expand our theoretical calculations accordingly.

**Reviewer 3**: The manuscript by Liu and coauthors reported a new reaction pathway for photocatalytic H2O2 production via *O2 dissociation process (*O2 → 2*O), which was achieved by using K and Cs co-modified polymeric carbon nitride (CN-KCs). The CN-KCs was synthesized by modifying polymeric CN via a straightforward hydrothermal method with the presence of KCl and CsCl at high temperature. The resulting CN-KCs exhibited high photocatalytic performance, attaining a H2O2 production rate of 1806.6 μmol h–1, a quantum efficiency of 95.4% at 420 nm, and a benchmark solar-to-H2O2 conversion efficiency of 6.1%. In general, modifying PCN for preparing photocatalysts is not new and has been reported elsewhere (International Journal of Hydrogen Energy, 2021, 46(77), 38310-38318, Journal of Hazardous Materials, 2022, 424, 127292, Dalton Transactions, 2022, 51(43), 16527-16535, International Journal of Hydrogen Energy, 2024, 51, 425-435). The reported procedures for CN-KCs synthesis based on hydrothermal method have no synthetic challenge. The structural design for CN-KCs is not clear as they did not clarify the advantages and purpose of utilization of K and Cs metals in this photocatalyst. The proposed new reaction pathway via *O2 dissociation process (*O2 → 2*O) is doubtful, particularly considering the involvement of dissociation of two strong chemical bonds. The theoretical calculations for H2O2 production are not completed and lack critical step for the formation of H2O2, so the conclusion made based on these results is too premature. In addition, the manuscript deserves a deeper interpretation of the data and a more detailed discussion in my opinion, particularly for the mechanism of the new process (*O + *H2O → H2O2). Considering the limited novelty and unclear scientific points, I cannot recommend its publication in Nature Communications.

**Response to comment (R)**: We thank Reviewer #3 for evaluating of our manuscript and providing comments and suggestions. Below we provide a point-by-point response to clarify the scientific novelty, structural design, and mechanistic insight of our work.

1. **Novelty of the catalyst design and difference from previously reported alkali-metal modified PCN**

The reviewer mentioned that modifying polymeric carbon nitride (PCN) with alkali metals is not new, citing several references. We agree that K/Na-modified PCN has been reported previously. However, our work differs from these studies in several essential aspects:

**(i) Distinct role of Cs and formation of new N–Cs dual active sites**

Although K is also present, our catalyst design primarily focuses on **Cs**, whose chemical behavior in PCN is far less explored. More importantly, we discovered that Cs incorporation creates **new N–Cs dual active sites**, which K alone cannot provide. These sites are crucial for the strong asymmetric adsorption of $O_2$, enabling the $*O_2 \rightarrow *O$ dissociation pathway. This fundamentally distinguishes our system from previous K/Na-modified PCNs.

**(ii) Different synthesis strategy enabling uniform intercalation**

In contrast to the widely used physical grinding–calcination approach in the cited literature, which often yields non-uniform alkali-metal distribution, we developed a **solution-based pre-intercalation strategy**. By first fully dissolving KCl and CsCl in water, followed by controlled drying and calcination, alkali-metal ions are uniformly inserted into PCN. This yields **homogeneously modified PCN with significantly enhanced catalytic performance**, far superior to conventional mechanical-mixing methods.

**(iii) Unique K/Cs synergistic effect demonstrated in systematic screening**

Our catalyst screening (Fig. 1a) included over **15 different single- and dual-alkali-metal combinations**. Among all tested systems, **only K/Cs co-modified PCN** exhibited the most outstanding activity, achieving:

- $H_2O_2$ production: 1806.6 μmol $h^{-1}$

- AQE at 420 nm: 95.4% (benchmark performance)
- Solar-to- H2O2 efficiency: 6.1%

This superior activity cannot be achieved with K, Na, or Cs alone, confirming the unique cooperative effect of K/Cs.

**2. Structural characterization and identification of K/Cs coordination environments**

In terms of structural elucidation, we have thoroughly characterized the catalyst using XPS, TEM, HAADF-STEM, and two synchrotron-based techniques: synchrotron radiation total scattering with pair distribution function (PDF) analysis, and X-ray absorption near-edge structure (XANES) spectroscopy, which collectively confirm the atomic-level coordination environment and the presence of adjacent K/Cs species. We acknowledge that the inherently low crystallinity of PCN limits precise crystal-structure modeling, but the combined experimental evidence unambiguously supports the formation of **dual $O_2$ adsorption sites (Cs–$N_2$)**.

**3. The discovery on *O dissociation pathway**

More importantly, in this work, we identified an unconventional inverse isotope effect over $H_2O$ during the reaction process, and, based on this observation, we proposed a new $*O_2$ dissociation pathway towards photocatalytic $H_2O_2$ synthesis is realized ($*O_2 \rightarrow 2*O$, then $*O + *H_2O \rightarrow H_2O_2$). To elucidate the reaction mechanism, we employed in situ X-ray photoelectron spectroscopy (XPS), temperature-programmed desorption (TPD), and attenuated total reflectance Fourier transform infrared spectroscopy (ATR-IR), to identify the nature and dynamic evolution of surface intermediates during the reaction. These experiments clearly support the formation of dual $O_2$ adsorption sites (Cs-$N_2$), and the subsequent dissociation of the O-O bond to form monodentate *O intermediates. And we also employed the kinetic isotope effect (KIE) studies using both $H_2O$ and EG as hydrogen sources, pH-dependent activity measurements, and Koutecky–Levich analyses, all of which consistently indicate that the reaction proceeds predominantly through the ORR route with direct O-O bond dissociation pathway rather than the traditional proton-coupled pathways via *OOH formation routes. Moreover, density functional theory (DFT) calculations were carried out to simulate the adsorption configurations and energy barriers, further verifying that the O-O bond cleavage occurs on the dual-end binding sites, leading to a new direct O-O dissociation ORR pathway toward $H_2O_2$ production.To exclude the possible contribution from the water oxidation reaction (WOR) pathway, we designed a set of control experiments, while the WOR pathway is negligible under our conditions.

Through the above clarifications, we believe that the novelty, scientific significance, and mechanistic insight of our work are much stronger than initially perceived. We sincerely hope that the reviewer may reconsider the scientific merit and originality of our work in light of these explanations.

Some detailed points:

**Q1.** The authors assumed that increasing the O2 adsorption is possible to reduce the energy of *O2 dissociation and cleavage of O=O bond, but the mechanism is unclear. Why

will the dissociation energy decrease? The two processes seem unrelated.

**R1**：We thank the reviewer for raising this important point. We agree that a simple increase in $O_2$ adsorption does not automatically guarantee a reduction of the O-O bond dissociation energy. In our system, however, the introduction of Cs does not merely “increase adsorption” in a general sense; instead, it creates a new type of dual-site adsorption configuration that directly facilitates O-O bond cleavage.

Specifically, the Cs incorporation creates a positively polarized site in close proximity to the intrinsic pyridinic-N sites. This forms a dual N–Cs adsorption motif, where $O_2$ binds in a highly asymmetric bidentate configuration: one O atom interacts with N, while the second O atom interacts with Cs. This asymmetric adsorption induces a strong differential charge redistribution and geometric stretching of the O-O bond, which is fundamentally different from simple physisorption enhancement. As a consequence, the O–O bond is already significantly elongated in the N–Cs dual-site configuration. This pre-activation reduces the energetic penalty required to cleave the O–O bond and allows spontaneous or low-barrier dissociation into *O + *O. Importantly, our DFT results show that in CN–KCs: O–O dissociation energy energy decreases substantially. The pathway *O-O → *O + *O becomes more favorable than the *O-O → *OOH hydrogenation step as the presence of Cs atom, which is not the case on pristine CN, where O-O cleavage is more difficult over CN.

Therefore, the effect is not due to nonspecific “stronger adsorption”, but due to a structural and electronic reconfiguration of the adsorption geometry induced by Cs, which stabilizes the transition state and weakens the O–O bond.

**Q2.** The proposed new pathway for photocatalytic $H_2O_2$ production is doubtful, as this process requires the cleavage of two strong covalent bonds, both O=O and H-O-H, to allow the intermediate *O react with $H_2O$ to produce $H_2O_2$ (H-O-O-H). Note that the cleavage of the H-O bond in $H_2O$ is an inevitable process for $H_2O_2$ photocatalysis and is also involved in the proposed new pathway.

**R2**：We sincerely thank the reviewer for the insightful comments regarding the proposed new reaction pathway. We would like to clarify that in the conventional PCET pathway for photocatalytic $H_2O_2$ production, the cleavage of the H-O bond in $H_2O$ is indeed indispensable. However, breaking the H-O bond requires a relatively high oxidation potential and substantial energy input, which is precisely the challenge that motivated our exploration of an alternative pathway.

In our proposed *oxygen-dissociation pathway*, the O–O bond of adsorbed $O_2$ is first cleaved to generate *O species, and these reactive *O intermediates subsequently couple directly with *$H_2O$ to form *$H_2O_2$, as shown in Equations 4–9. This mechanism **circumvents the need for H–O bond cleavage in water**, which is required in the classical PCET route.

More importantly, on the CN–KCs catalyst, the introduced Cs atoms create asymmetric dual-site adsorption (N + Cs) and facilitate electron injection into the π* antibonding orbitals of $O_2$. These effects collectively elongate the O–O bond, reduce its bond order, and significantly lower the energy barrier for O–O cleavage. As a result, O–O bond dissociation becomes energetically more favorable than H–O bond cleavage under our catalytic environment.

In summary, the enhanced O–O bond dissociation on CN–KCs originates from:
(1) asymmetric dual-site adsorption of $O_2$,
(2) electron transfer from the catalyst into the $O_2$ π* antibonding orbital, and
(3) the presence of Cs as a strong adsorption site.

These factors synergistically weaken the intrinsic O=O double bond and substantially reduce its dissociation barrier, enabling our proposed pathway.

For CN-Cs and CN-KCs, the presence of Cs introduces a new oxygen dissociation pathway. The formed $*O_2$, due to its strong adsorption, tends to dissociate to form *O, which then directly couples with $*H_2O$ to generate $*H_2O_2$ following equations 4-9:

$$O_2+2* \rightarrow *O\text{-}O* \quad \text{(on double N+Cs sites)} \quad (4)$$

$$*O\text{-}O*+ e^- \rightarrow *O+ O^- \quad (5)$$

$$*O+ *H_2O \rightarrow *H_2O_2 \quad (6)$$

$$2O^- +*H_2O \rightarrow *H_2O_2 +2OH^- \quad (7)$$

$$HO\text{-}CH_2\text{-}CH_2OH\ (EG)+ 2h^+ \rightarrow HO\text{-}CH_2\text{-}CHO +2H^+ \quad (8)$$

$$2OH^- +2H^+ \rightarrow 2H_2O \quad (9)$$

**Q3.** The bond energy of $O_2$ (O=O) is 498 kJ/mol, while the bond energy of O-H in $H_2O$ is 463 kJ/mol, so $*O_2$ dissociation process ($*O2 \rightarrow 2*O$) requires higher energy input than the cleavage of H-O. The new pathway is an energy-unfavorable process.

**R3:** Q3 appears to be an extension of Q2, and the specific explanation can be found in our response to R2.

**Q4.** The contents of K and Cs in the CN-KCs are not known. How the content affecting the photocatalytic performance is unclear.

**R4:** ICP-OES reveals that the contents of K and Cs in CN-KCs are 6.8 wt% and 21.3 wt%, respectively. The average $H_2O_2$ generation rate at different different K and Cs loadings is shown is Figure R11 (Supplementary Fig. 3.)

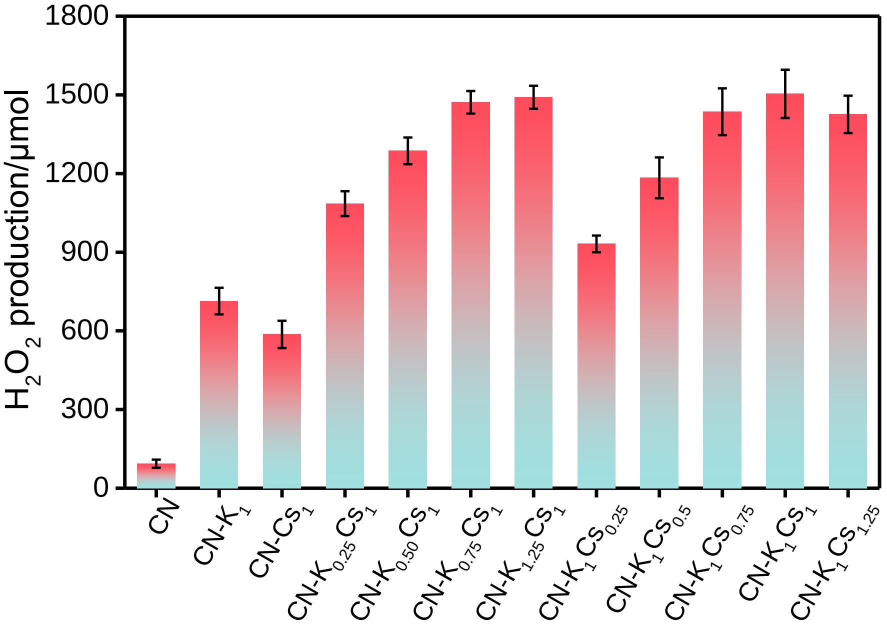


**Figure. R11.** The average $H_2O_2$ generation rate at different different K and Cs loadings using CN-KCs catalyst with air, 20 mL of a 0.5% (v/v) EG aqueous solution, 25 °C and illuminated by white LED lamp at an intensity of 100 mW/cm$^2$.

**Q5.** In Fig. 6e-g, $H_2O_2$ production based on *O + *$H_2O$ → $H_2O_2$ is not completed as the intermediate *O needs to be inserted into H-O-H to form new chemical bond (H-O-O-H). The dissociation of H-O-H and the reaction of *O and *O-H should be included in the theoretical calculations. One-step formation of $H_2O_2$ via*O + *$H_2O$ → $H_2O_2$ is impossible.

**R5**: We sincerely thank the reviewer for this insightful comment. We agree that in traditional proton-coupled electron transfer (PCET) pathways, $H_2O_2$ formation involves O–H bond cleavage. However, in our system, the *O intermediate generated from O–O bond cleavage is highly reactive and positioned on the dual-end Cs–N sites, which strongly polarizes the adjacent $H_2O$ molecule. This allows the formation of $H_2O_2$ via a **concerted O–H bond rearrangement** rather than full O–H bond dissociation. In other words, the reaction proceeds through an **O–H bond shift within** $H_2O$, forming H–O–O–H, without the need for separate proton abstraction and electron transfer steps. This step is strongly supported by the Inverse kinetic isotope effect (KIE)**:** indicating that H–O cleavage is not rate-limiting and the DFT calculations**:** revealing that the energy barrier for *O + * $H_2O$ → $H_2O_2$ via O–H bond rearrangement is lower than that for the traditional PCET *OOH formation route.

We therefore consider the "*O + * $H_2O$ → $H_2O_2$" notation as a reasonable one-step representation of the overall process under our catalytic conditions, which captures the essence of the direct O–O bond dissociation pathway toward $H_2O_2$.

**Q6.** Lines 186-188: the author supposed that the Cs donates electron in CN-KCs, while this is difficult to understand as the cationic Cs+ ion used in preparation cannot provide electron anymore.

**R6**: We appreciate the reviewer's concern. This is mainly because Cs possesses an extremely low electronegativity (0.79), and therefore behaves as a strong electron-donating species when interacting with the heptazine units in PCN. It should be noted that Cs does not form a localized covalent bond with the N sites of the heptazine ring; instead,

it interacts with these N atoms through electrostatic coordination, which increases the electron density on the adjacent N atoms. Consequently, the neighboring C atoms become relatively more electron-deficient, leading to an enhanced polarization of the C=N bond and a strengthened π* absorption.

**Q7.** TA results showed that CN-KCs possessed the shortest lifetime for charge separated states, which is unfavoured for the utilization of photoinduced electrons and holes.

**R7**: We thank the reviewer for this important point. We acknowledge that the lifetime of charge-separated states in CN-KCs is shorter than that of pristine CN, which indeed represents a drawback of the CN-KCs catalyst. This shortening mainly originates from the presence of Cs. For example, the average lifetime of CN and CN-Cs is 114.5 ps and 47.2 ps, respectively, whereas that of CN-KCs is 54.0 ps, indicating that the introduction of K does not lead to faster carrier recombination.

The reduced lifetime induced by Cs is likely due to the formation of new adsorption centers at Cs sites, which attract photogenerated carriers toward the Cs-associated regions. In the presence of $O_2$, these carriers can be rapidly transferred to the adsorbed $O_2$ molecules, thereby facilitating strong $O_2$ adsorption and activation.

This interpretation is fully consistent with the central conclusion of our work: the primary role of Cs is to enhance the adsorption strength of $O_2$ and key reaction intermediates, lower the associated adsorption/transition energy barriers, and ultimately shift the reaction pathway toward a faster and more efficient process.

**Q8.** Lines 163-164: the authors indicated that K ions are located within the nitride pots among the three tri-s-triazine units of CN, but K does not possess the d orbitals to coordinate with N.

**R8**: We thank the reviewer for raising this important point. We agree that $K^+$ does not utilize d-orbitals to form classical coordination bonds with nitrogen. In our manuscript, the term "located within the nitride pots" does not imply a d-orbital–based coordination. Instead, $K^+$ is stabilized in the nitride pots of the tri-s-triazine units through electrostatic embedding / ion–dipole interactions with the electron-rich pyridinic N atoms.

Such interactions have been reported for alkali metal cations ($Na^+$, $K^+$, $Cs^+$) in carbon nitride frameworks (e.g., Angew. Chem. Int. Ed. 2017, 56, 13445.; Angew.Chem.Int. Ed. 2018, 57,9372 –9376). These ions do not require d-orbitals for binding; rather, their stabilization arises from the strong electrostatic potential inside the heptazine cavity and the polarization of nearby C–N bonds. To avoid confusion, we have revised the wording in the manuscript to explicitly state that $K^+$ is "electrostatically embedded within the three tri-s-triazine units" rather than "coordinated", and we have added a citation to the relevant literature.

**Reviewer 4**: This paper reports very impressive efficiencies for H2O2 production in on Cs loaded carbon nitride photocatalysts in the presence of glycol as electron source. The quantum efficiencies and stability are both impressive and suitable for publication in nature communications. I note the authors also also claim very high solar to chemical conversion (SCC) efficiencies. It is not clear to me how these are calculated, and in particular how the change in energy of the glycol to products reaction is included (which it must be if quoting a SCC efficiency). It is not clear to me SCC is a useful assay for such reactions.

The paper includes a extensive characterisation and analysis. This is the weaker side of the paper, in my opinion. While some analyses give insight into the reaction mechanism,

and are interesting, others appear to me to give little clear insight, with conclusions not clearly justified by the results. For example, the fs-TAS results essentially show that adding Cs, the optical signal decays faster. This could potentially be due to electron transfer to Cs, but there is no direct evidence of this. There is also, I think, no evidence for a change in electron trapping. The description of the conclusions from these results should be revised to be limited to those which can be clearly concluded from the data.

The authors end their paper with some reaction mechanisms, which appear to be based on theory calculations. it is unclear how the experimental data supports, or indeed may question, these proposed mechanisms - this discussion needs to be added. In particular, it should be clarified whether the KIE, FTIR and XPS data support these mechanisms. It is not necessary that everything agrees - indeed this is a complex system where I would expect the interpretation of the results to often be quite ambiguous. But it is important for readers to be guided more clearly on what can be clearly concluded from the results, and what is suggested but not proven.

Overall I think the results in this manuscript are suitable for publication in nature communications. However the manuscript does need significant revisions, primarily in the discussion and conclusions which can be drawn from the analysis results.

**R1**： We thank the reviewer for raising this important and thoughtful question regarding the definition and validity of the solar-to-chemical conversion (SCC) efficiency in our reaction system.

In our work, the SCC efficiency is defined as the ratio between the chemical free energy stored in the target product $H_2O_2$ and the total incident solar energy, following the convention commonly adopted in photocatalytic $H_2O_2$ production systems. We emphasize that this SCC value is an apparent efficiency that specifically quantifies the **light-to-chemical energy conversion toward $H_2O_2$ formation, rather than the overall thermodynamic efficiency of the entire reaction system.**

0.5% (v/v) Ethylene glycol (EG) in this system is intentionally introduced as a hole scavenger to address the sluggish kinetics of water oxidation half-reaction. The oxidation of EG is a thermodynamically favorable process and serves to consume photogenerated holes, but its chemical energy change is not included in the SCC calculation. Therefore, the SCC reported here just reflects the efficiency of converting solar energy into the chemical energy stored in $H_2O_2$, under assisted conditions, rather than a closed-cycle solar fuel efficiency.

We fully agree with the reviewer that SCC may not represent an absolute energy efficiency for such multi-component reaction systems. Nevertheless, it remains a useful and widely adopted metric for benchmarking and comparing the light utilization efficiency among different photocatalytic $H_2O_2$ systems operated under similar conditions with sacrificial agents. To avoid potential misunderstanding, we have revised the manuscript to explicitly clarify the definition, physical meaning, and limitations of the SCC efficiency used in this study.

The detailed calculation is provided in Supplementary Note 2.

**Supplementary Note 2, the solar-to-chemical conversion (SCC) efficiency calculation**

The SCC efficiency on CN-KCs is calculated by the following equation :

$$SCC(\%)=\frac{\Delta G_{H_2O_2}\times N_{H_2O_2}}{I\times S\times T}\times 100\%$$

Where $\Delta G_{H2O2}$ represents the Gibbs free energy change associated with the formation of $H_2O_2$ (117 kJ/mol), $N_{H2O2}$ stands the moles of produced $H_2O_2$ of which was calculated to be 1806.6 μmol $h^{-1}$. I signifys the light intensity of simulated sunlight (100 mW $cm^{-2}$), S represents the illuminated surface area irradiation area of 9.616 $cm^2$, and T means the irradiation time of 3600 s. As such, the calculated total input energy was 3461.76 J, the power generated by $H_2O_2$ formed was 211.37 J.

$$SCC(\%)=\frac{117\times 10^{3}\times 1806.6\times 10^{-6}}{100\times 10^{1}\times 9.616\times 10^{-4}\times 3600}\times 100\%$$

$$=\frac{211.37\ J/s}{3461.76\ W}\times 100\%=6.1\%$$

It should be noted that the SCC value reported here does not account for the chemical energy change associated with EG oxidation, and therefore should be regarded as a comparative metric rather than an absolute thermodynamic efficiency.

**R2**: We sincerely thank the reviewer for the careful reading of our manuscript and for pointing out the limitations in the mechanistic interpretation of the fs-TAS measurements. We agree that ultrafast transient absorption mainly reflects the decay kinetics of photoexcited states, and that the current data cannot unambiguously prove electron transfer to Cs sites or definitively confirm changes in the nature of electron trapping.

Our original intention was to use the faster decay of the excited-state signal in CN-Cs to indicate accelerated carrier relaxation dynamics, which is consistent with (though not direct proof of) improved charge extraction or accelerated interfacial reactions. This interpretation aligns with the enhanced ORR activity and the DFT-predicted Cs-induced electronic polarization, but we fully acknowledge that fs-TAS alone cannot isolate the specific microscopic pathway (e.g., Cs-mediated electron transfer vs. modified trap-state distribution).

**R3**: We sincerely thank the reviewer for highlighting the need for a clearer connection between our experimental results and the proposed reaction mechanisms derived from theoretical calculations. We fully agree that, for a complex photocatalytic system such as ours, it is essential to distinguish:

1. which mechanistic elements are directly supported by experiments, and
2. which parts are suggested by DFT but remain hypothetical or partially supported.

To address the reviewer's concern, we will revise the Discussion section to explicitly relate each key experimental technique (KIE, in-situ FTIR, and XPS) to the proposed mechanistic steps as follows:

(1) KIE measurements

Kinetic isotope measurements indicate that proton transfer is not the rate-limiting step, consistent with our DFT-predicted pathway in which O-O activation rather than O-H bond cleavage dominates the energetics. We will clarify that the KIE does not directly prove the $O_2$ dissociation pathway, but it rules out classical PCET being rate-limiting, which is consistent with our proposed mechanism.

(2) In-situ FTIR

In-situ FTIR shows that Cs-containing catalysts (CN-Cs and CN-KCs) exhibit a shifted $O_2$-adsorption band (1577 → 1581 $cm^{-1}$) and the emergence of a new vibration at 733 $cm^{-1}$, assigned to an *O-related bending mode. These features indicate that $O_2$ is more strongly activated on Cs-modified surfaces and that *O-derived intermediates are formed, although FTIR does not directly prove complete O-O bond dissociation. The much stronger *O signal on CN-KCs, combined with the appearance of * $H_2O_2$ bands at 1312-1314 $cm^{-1}$, correlates well with the higher photocatalytic activity observed for this catalyst. In contrast, CN and CN-K show no detectable *O signals and only weak * $H_2O_2$ features. The light-dependent growth of all ORR-related peaks further confirms that these intermediates are generated through photon-driven processes. Overall, FTIR provides experimental evidence for enhanced $O_2$ activation on Cs-containing catalysts and offers mechanistic insights consistent with, though not independently proving, the DFT-predicted dual-site activation pathway.

(3) XPS

The in-situ XPS data clearly support the presence of differentiated adsorption behaviors toward $O_2$ and $H_2O$ on CN-KCs. Under light + $O_2$, the Cs 3d peak shows a noticeable positive shift, indicating that excited electrons near Cs are extracted by adsorbed $O_2$, thereby strengthening the Cs-O interaction. This observation is consistent with the proposed dual adsorption site (Cs–$N_2$) involved in $O_2$ activation. In contrast, under light + $H_2O$, the Cs 3d signal remains essentially unchanged, suggesting that $H_2O$ interacts only weakly with Cs sites, in agreement with the TPD results.

While the XPS shift does not directly prove O–O bond dissociation, it supports the mechanistic proposal that $O_2$ is strongly bound at the Cs-related site. Together with ATR-IR results that provide direct evidence of monodentate *O species, the XPS data corroborate—but do not independently confirm—the dual-end adsorption mode that facilitates O-O bond cleavage.

As the reviewer correctly notes, such complex catalytic systems inevitably involve interpretations that are somewhat ambiguous. In the revision, we will clearly separate:

- **Experimentally verified observations** (e.g., enhanced $O_2$ adsorption, $O_2$ -derived intermediates, non-PCET rate-determining step), from
- **Mechanistic suggestions primarily supported by theory** (e.g., precise sequence of *O formation and its coupling with *$H_2O$).

This restructuring will guide readers more transparently regarding the degree of certainty associated with each mechanistic step.

,

We were pleased to receive your letter concerning the revisions required for our manuscript. We sincerely appreciate the valuable support and thoughtful input provided by you and the reviewers, which have greatly contributed to improving the clarity and quality of our manuscript. Over the past few days, we have carefully conducted additional experiments to address each of the reviewers' comments. Specifically, fs-TA measurements under $O_2$ were carried out, hydroxyl radical trapping experiment with p-hydroxybenzoic acid was performed to trace the oxygen source in $H_2O_2$, in-situ FTIR spectra under $^{18}O_2$ was tested, QE calculation was corrected, and DFT calculations were re-conducted according to reviewer's suggestions. The newly acquired data have also been subjected to statistical analysis. We now submit the revised manuscript, in which all the points raised by the reviewers have been thoroughly responded. Below, we provide a detailed, point-by-point description of the revisions made in response to the reviewers' comments.

## Response to the reviewers' Comments

Many thanks to the reviewers for their valuable comments and suggestions. The followings are the point-by-point answers to the concerns:

**Reviewer #2:** The authors didn't respond properly to some key comments. Judging from response 1, the data in the manuscript lacks credibility. Moreover, the mass results cannot match the proposed $O_2$ dissociation mechanism. So, I do not recommend this manuscript for publication. Below are the detailed comments:

**Q1.** Regarding response 1: The authors stated that "In Supplementary Fig. 4b, the y-axis label should read 'μmol instead of 'μM.' The $H_2O_2$ yield of 1806.6 μmol reported in the main text for CN-KCs is correct." in response 1. However, the colorimetric DPD method for detecting $H_2O_2$ has a significant flaw, that when H2O2 concentration exceeds a certain value, DPD·+ with typical absorption in 551 nm will be overoxidized and the absorbance will decrease significantly (Chemosphere, 2019, 224, 646-652.). I think the authors know the phenomenon, as the detection of reaction supernatant is after a 10-fold dilution. However, the claimed correct unit "μmol" results in the concentrations of $H_2O_2$ far exceeding the detectable limit. The acquisition of updated standard fitting curve of $H_2O_2$ concentration is impossible. The relationship between the absorbance of DPD·+ and the concentration of $H_2O_2$ can refer to the references (Small, 2018, 14(9): 1703142; Chemosphere, 2019, 224, 646-652; Angewandte Chemie International Edition, 2022, 61, e202200413.). Furthermore, there is no need for 10-fold dilution of reaction supernatant, if the "μmol" is the correct unit. 400 μmol $H_2O_2$ in 1 mL solution to be tested corresponds to 8000 μmol $H_2O_2$ in reactor (total

volume is 20mL), which is larger than the so-called $H_2O_2$ yield of 1806.6 μmol. Given that the author insists "μmol" is the correct unit, the rest of data is deemed lack of reliability. Also, the real production of $H_2O_2$ significantly reduce the importance of this work.

**R1:** We sincerely thank the reviewer for the careful analysis and for raising this important concern regarding the reliability of the DPD colorimetric method at high $H_2O_2$ concentrations. We also greatly appreciate the insightful references provided. After carefully reading the cited literatures (Small, 2018; Chemosphere, 2019; Angew. Chem. Int. Ed., 2022) and comparing them with our experimental procedure, we believe that the inconsistency mainly arises from our unclear expression of how the $H_2O_2$ amount and concentration were defined and quantified in our work.

In the referenced studies, the calibration curves are typically constructed based on $H_2O_2$ concentration (μmolL$^{-1}$) without explicitly considering the total reaction volume. In contrast, in our work, the reported value (μmol) refers to the total amount of $H_2O_2$ produced in the entire reaction system (20 mL), rather than the concentration in the aliquot used for DPD measurement.

Specifically, during our calibration and detection procedure, a known amount of $H_2O_2$ was first added into a 20 mL solution to match the actual reaction volume, and then a 1 mL aliquot was taken for DPD analysis. Therefore, the reviewer's assumption that "400 μmol $H_2O_2$ in 1 mL corresponds to 8000 μmol in 20 mL" does not apply to our system. Instead, it should be 400 μmol $H_2O_2$ distributed in the total 20 mL solution, followed by aliquot sampling for measurement.

Regarding the concern about DPD overoxidation at high concentrations, we would like to clarify that the 10-fold dilution step was intentionally applied to ensure that the measured absorbance falls within the linear detection range of the DPD method, thereby avoiding the overoxidation issue mentioned by the reviewer. The calibration curve used in our study was established under the same dilution and measurement conditions to ensure consistency and accuracy.

We acknowledge that the description of our quantification procedure in the original manuscript was not sufficiently clear. In the revised manuscript, we provide a more detailed explanation of the calibration method, dilution procedure, and the distinction between $H_2O_2$ amount (μmol) and concentration (μmol L$^{-1}$) to avoid any confusion.

Once again, we sincerely appreciate the reviewer's careful and responsible evaluation, which has helped us improve the clarity and rigor of our work.

**Q2.** Regarding response 2 and 3: Please refer to comment 1.

**R2:** Please refer to **R1.**

**Q3.** Regarding response 6: If the fs-TA measurements are conducted in Ar, the shorter

lifetime in CN-KCs is unfavored for the subsequent reaction, which I and reviewer 3 both deemed. The authors gave the explanation that “The reduced lifetime induced by Cs is likely due to the formation of new adsorption centers at Cs sites, which attract photogenerated carriers toward the Cs-associated regions. In the presence of $O_2$, these carriers can be rapidly transferred to the adsorbed $O_2$ molecules, thereby facilitating strong $O_2$ adsorption and activation.” However, under light and UHV, there is no change in the binding energy of Cs 3d (Fig 3f), indicating no photoelectron transfers to Cs, which is contradictory to the given explanation. Furthermore, the viewpoint that carriers can be rapidly transferred to the adsorbed $O_2$ is lack of evidence. The fsTA measurements in the presence of $O_2$ are essential for comparison with those performed in Ar.

**R3:** We sincerely thank the reviewer for this important and insightful comment. We fully agree that, in general, a shorter lifetime of charge-separated states may appear unfavorable for photocatalytic reactions, and we appreciate the reviewer for pointing out the apparent inconsistency between the fs-TA and XPS results.

First, we would like to clarify that the in situ XPS results under light and UHV conditions show no noticeable shift in the Cs 3d binding energy, which indeed indicates that Cs does not act as a **direct electron acceptor** in the absence of adsorbates such as $O_2$. We agree that our previous wording may have led to confusion, and we have revised it accordingly.

However, it is important to emphasize that under **reaction-relevant conditions (i.e., in the presence of $O_2$ and light irradiation)**, the behavior of Cs is markedly different. As shown in our in situ XPS measurements (Fig.R1), the Cs 3d peak exhibits a clear shift (~0.2 eV) toward higher binding energy in the presence of $O_2$ under illumination. This shift indicates a **decrease in electron density around Cs**, which can be reasonably attributed to **photoinduced electron transfer from the catalyst to adsorbed $O_2$ molecules mediated by Cs-associated sites**.

This interpretation is further supported by **TPD results** (Fig.R2), which show a significant increase in both the adsorption amount and adsorption strength of $O_2$ upon Cs incorporation. Notably, apart from the intrinsic $N_2$ site in carbon nitride (~141.8 °C), $O_2$-TPD profiles reveal the emergence of an additional desorption peak (~182.4 °C) after Cs incorporation (CN-KCs), which is absent in pristine CN and can be assigned to newly formed Cs-related $O_2$ adsorption sites. This result indicates the formation of dual Cs–$N_2$ adsorption sites that enable stronger and asymmetric $O_2$ binding, consistent with the proposed role of Cs in facilitating $O_2$ activation and subsequent electron transfer. These observations consistently indicate that Cs plays a crucial role in **enhancing $O_2$ adsorption and facilitating interfacial electron transfer**, rather than acting as a standalone electron acceptor.

Regarding the fs-TA results, we would like to emphasize that the measurements were conducted under **Ar atmosphere**, where no external electron acceptor is present. Under such conditions, the shortened lifetime observed for CN-KCs may also be attributed to **faster carrier migration toward surface-active sites**, rather than purely increased recombination. In contrast, under actual reaction conditions with $O_2$ present, these carriers can be efficiently extracted by adsorbed $O_2$, leading to enhanced catalytic performance.

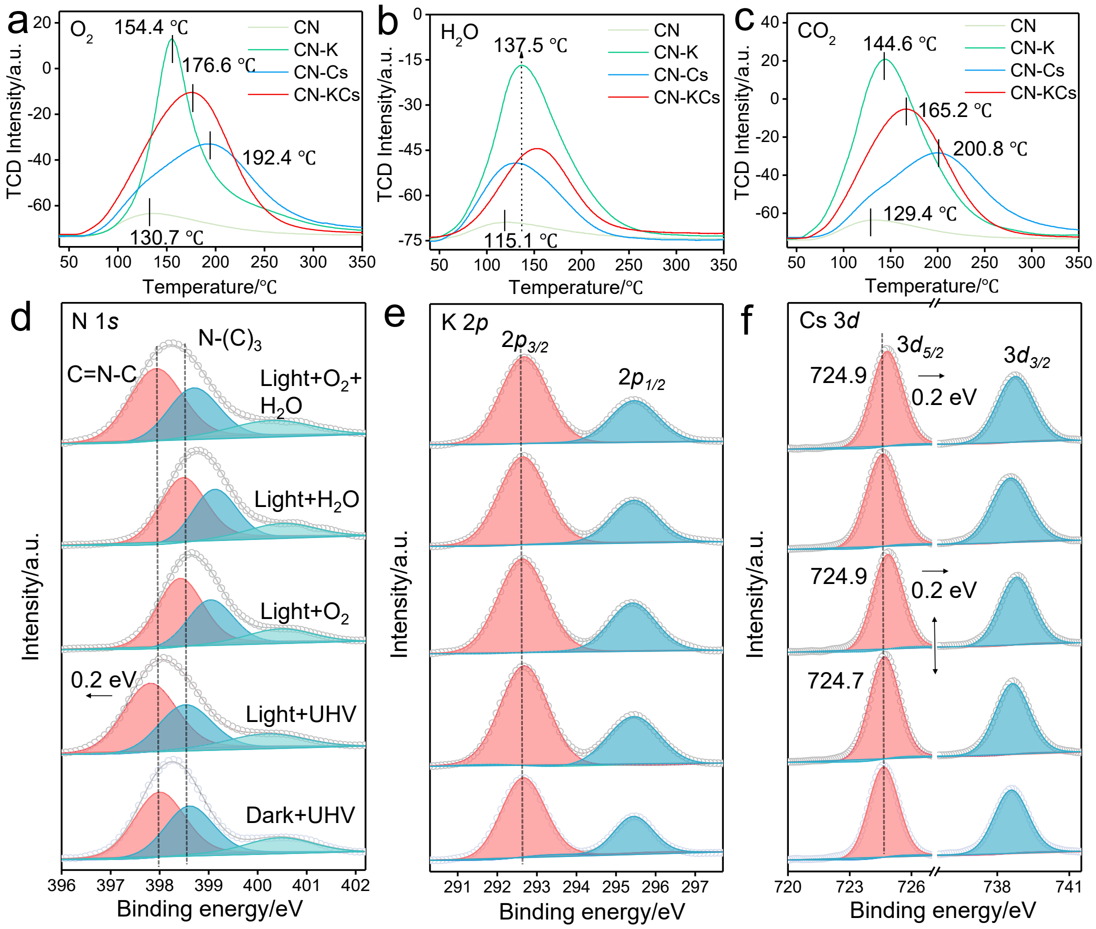


**Fig. R1. Investigation of surface absorption capacity and chemical state variation.** TPD spectra of **a**. $O_2$, **b**. $H_2O$ and **c**. $CO_2$ on catalysts. In situ XPS experiments of **d**. N 1*s*, **e**. K 2*p* and **f**. Cs 3*d* spectra under different condition on catalysts.

We fully agree with the reviewer that fs-TA measurements under $O_2$ atmosphere would provide more direct insight into the interfacial charge transfer process. Following this suggestion, we conducted additional fs-TA experiments in the presence of $O_2$ using CN-KCs. Preliminary results suggest a further acceleration of the decay dynamics, with the lifetime decreasing from 54.0 ps to 46.7 ps (Fig. R3), supporting the role of $O_2$ as an efficient electron acceptor. In addition, the 2D transient absorption mapping under

$O_2$ atmosphere exhibits a similar ground-state bleaching (GSB) spectral range compared to that under Ar, while showing a slightly reduced signal intensity, which can be attributed to more efficient extraction of photogenerated electrons by $O_2$.

Therefore, we would like to emphasize that the key factor is not the absolute carrier lifetime itself, but rather the **competition between charge recombination and interfacial charge transfer**. In CN-KCs, Cs-induced adsorption sites promote strong $O_2$ binding and enable rapid electron extraction under reaction conditions, which outweighs the apparent disadvantage of shortened lifetime and ultimately leads to enhanced photocatalytic activity.

We have revised the manuscript to clarify these points and avoid any overinterpretation. We sincerely appreciate the reviewer's insightful suggestion, which has significantly improved the rigor of our discussion.

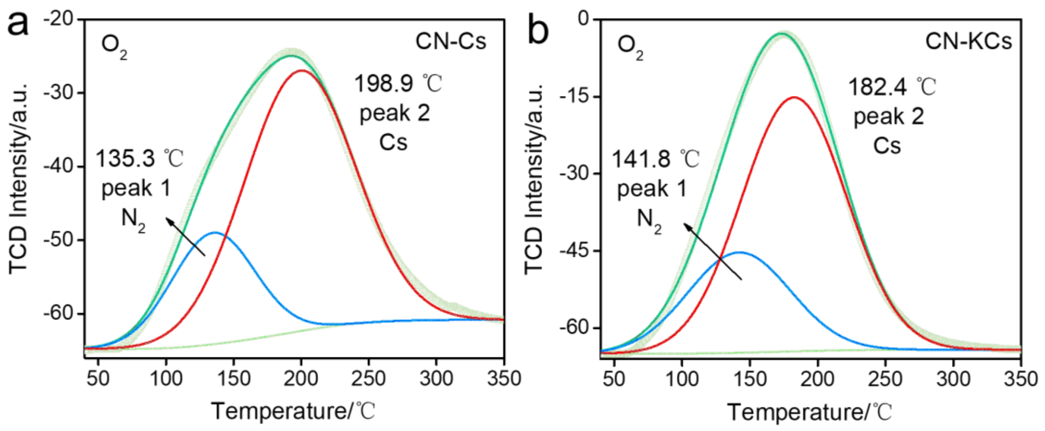


**Fig. R2.** Temperature-programmed desorption (TPD)-$O_2$ spectra of (a) CN-Cs and (b) CN-KCs. The original spectrum was fitted into two peaks: one is the adsorption peak of the intrinsic N sites of CN, and the other peak, represented by the red line, corresponds to the adsorption peak of $O_2$ at the Cs sites.

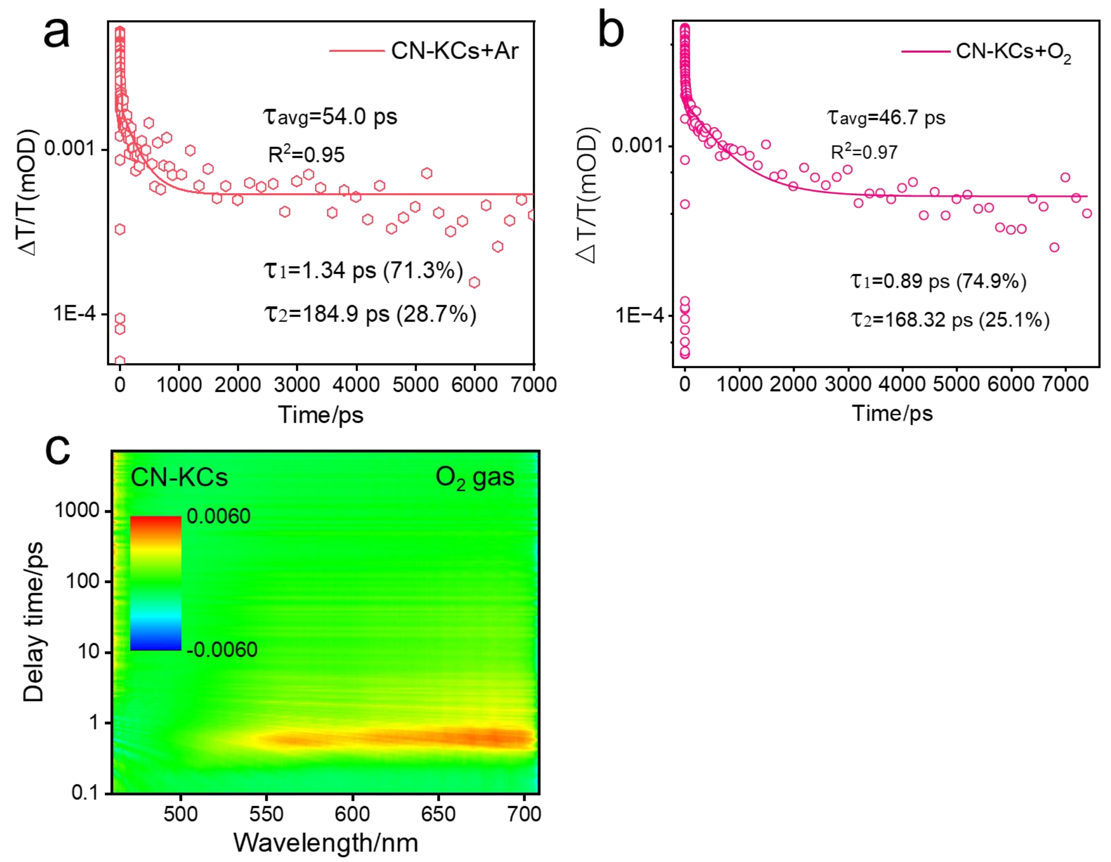


**Fig. R3.** fs-TA decay spectra at 600 nm over CN-KCs catalyst under (a) Ar gas and (b) $O_2$ gas. (c) 2D mapping femtosecond transient absorption (fs-TA) spectroscopy measurements of CN-KCs under $O_2$ gas.

**Q4.** Regarding response 9: In Fig 3i, the use of triethylamine instead of EG makes a 1.28 folds enhancement of $H_2O_2$ production. Whereas, in QE calculation, the $H_2O_2$ production in the presence of TEA only gives only a 1.03 folds enhancement compared to EG. Why?

**R4:** We thank the reviewer for this valuable and insightful question. The discrepancy between the overall activity enhancement (1.28×) and the QE enhancement at 420 nm (1.03×) can be rationalized by the following factors.

First, the 1.28-fold enhancement in $H_2O_2$ production was obtained under broad-spectrum visible-light irradiation, whereas the QE value reflects the catalytic performance at a single wavelength (420 nm). The wavelength-dependent activity differs between TEA and EG systems. For example, at longer wavelengths (e.g., ~450 nm), the QE using TEA is more than 1.1 times that using EG. Therefore, when integrated over the entire visible-light spectrum, these wavelength-dependent differences lead to a larger overall enhancement compared to that observed at a single excitation wavelength.

Second, the ORR process in our system proceeds via two parallel pathways,

namely the conventional two-electron PCET pathway and the *O-mediated dissociation pathway. In our initial QE calculation, only the two-electron PCET contribution was considered, which may have led to an overestimation of the QE values. We have therefore recalculated the wavelength-dependent QE values (Fig. R4) by taking both pathways into account.

In addition, since TEA is more readily oxidized than EG, it can more efficiently scavenge photogenerated holes and promote charge separation, which may alter the relative contributions of the two pathways under different irradiation conditions. This difference can further contribute to the inconsistency between broadband activity and monochromatic QE results.

We have clarified these points in the revised manuscript and provided a detailed description of the QE calculation procedure to ensure transparency and avoid potential misunderstanding.

"The number of reacted electrons ($N_e$) is no longer simply $2\times N_{H2O2}$. Instead, it is calculated as follows: $N_e$=2×PCET yield of $H_2O_2$ (51.5%) +1×single electron of $H_2O_2$ (48.5%).

"To calculate the AQE at the wavelength of 420±2 nm on using CN-KCs, the calculated process as follows:

$$N_e\text{=number of reacted electrons (mols}^{-1}\text{)}$$

$$=2\times\frac{n2}{t}+1\times\frac{n1}{t}=\frac{(2\times372.83+1\times351.12)\times10^{-6}}{3600}=3.047\times10^{-7}\ \text{mols}^{-1}$$

The number of $H_2O_2$ molecules from the production rate of $H_2O_2$ under 420 nm was calculated to be 723.95 μmol $h^{-1}$. Accordingly, the $H_2O_2$ generated via the single-electron reduction pathway is 351.12 μmol $h^{-1}$ (723.95×48.5%), while that produced through the two-electron proton-coupled electron transfer (PCET) process is 372.83 μmol $h^{-1}$. Then, the received reacted electron number of the $H_2O_2$ products ($N_e$) is measured as $(2\times372.83+1\times351.12)\times10^{-6}/3600=3.047\times10^{-8}$ $mols^{-1}$.

The energy of a single photon ($E_s$) at λ = 420 nm is calculated according to hc/λ, where h and c are the Planck constant $6.63\times10^{-34}$ J s and the speed of light $3\times10^{8}$ m/s, respectively, $E_s$ was found to be $4.736\times10^{-19}$ J.

$$E_s=\frac{hc}{\lambda}=\frac{6.63\times10^{-34}\times3\times10^{8}}{4.2\times10^{-7}}=4.736\times10^{-19}\ \text{J}$$

The Avogadro's number (denoted as A) representing the number in one mole of a substance is $6.022\times10^{23}$. The incident light intensity (I) was measured as 12.5 $mW/cm^2$, calibrated by a light power meter (PL-MW2000). The reactor's illumination window, approximately 3.5 cm in diameter, yielded an irradiation area (S) of 9.616 $cm^2$.

Consequently, the total number of photons incident (Np) offered in the reaction vessel per hour was calculated to be $4.214\times10^{-7}$ mol $s^{-1}$.

$$N_p=\frac{I\times S}{E_s\times A}=\frac{12.5\times10^{1}\times9.616\times10^{-4}}{4.736\times10^{-19}\times6.022\times10^{23}}=4.214\times10^{-7}$$

Thus, according to equation (1), the AQE (%) =72.3%.

$$AQE(\%)=\frac{N_e}{N_P}=\frac{\text{number of reacted electrons}}{\text{number of incident photons}}\times100\%$$

$$=\frac{3.047\times10^{-7}}{4.214\times10^{-7}}\times100\%=72.3\%$$

**Take AQY at the wavelength of 385±2 nm of CN-KCs as an other example,**

The number of $H_2O_2$ 744.8molecules from the production rate of $H_2O_2$ under 385 nm was calculated to be 352.32 μmol $h^{-1}$. Accordingly, the $H_2O_2$ generated via the single-electron reduction pathway is 170.88 μmol $h^{-1}$ (352.32×48.5%), while that produced through the two-electron proton-coupled electron transfer (PCET) process is 181.44 μmol $h^{-1}$. Consequently, the total number of reacted electrons for the $H_2O_2$ products ($N_e$) is determined to be $(2\times181.44+1\times170.88)\times10^{-6}/3600=1.483\times10^{-7}$ $mols^{-1}$.

$$N_e=\text{number of reacted electrons } (mols^{-1})$$

$$=2\times\frac{n2}{t}+1\times\frac{n1}{t}=\frac{(2\times181.44+1\times170.88)\times10^{-6}}{3600}=1.483\times10^{-7}\ mols^{-1}$$

The energy of a single photon ($E_s$) at λ = 385 nm is calculated to be $5.234\times10^{-19}$ J, the light intensity (I) was measured as 6.5 mW/$cm^2$. Consequently, the total number of photons incident (Np) offered in the reaction vessel per hour was calculated to be $1.983\times10^{-7}$ mol $s^{-1}$.

$$N_p=\frac{I\times S}{E_s\times A}=\frac{6.5\times10^{1}\times9.616\times10^{-4}}{5.234\times10^{-19}\times6.022\times10^{23}}=1.983\times10^{-7}$$

Thus, according to equation (1), the AQE (%) =74.8%.

$$AQE(\%)=\frac{N_e}{N_P}=\frac{\text{number of reacted electrons}}{\text{number of incident photons}}\times100\%$$

$$=\frac{1.483\times10^{-7}}{1.983\times10^{-7}}\times100\%=74.8\%$$

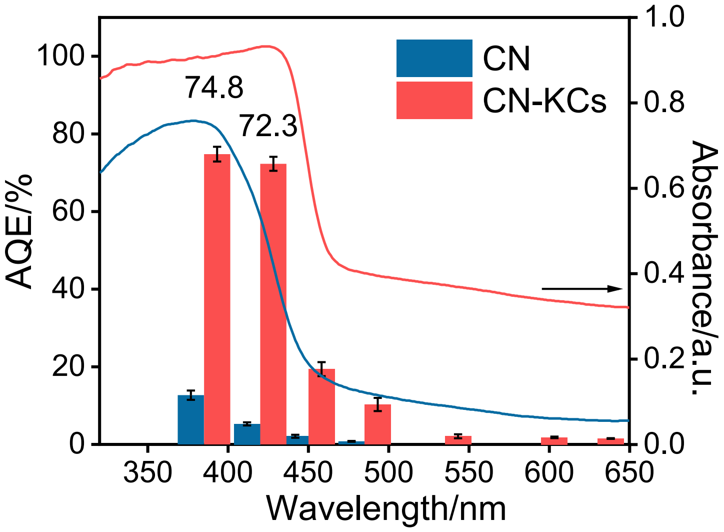


**Fig. R4.** Apparent quantum efficiencies (AQE) for $H_2O_2$ product at specific incident wavelengths, together with the DRS spectra of CN and CN-KCs.

**Q5.** As the authors claimed that only 35-50% of the $H_2O_2$ is generated via *O + *$H_2O$ pathway and 50-65% of the $H_2O_2$ is generated via PCET, the KIE value for CN-KCs smaller than 1 is unreasonable.

**R5:** We sincerely thank the reviewer for this insightful comment. We agree that, at first glance, a KIE value smaller than 1 may appear inconsistent with the coexistence of both PCET and *O-mediated pathways. However, we would like to clarify that the observed inverse KIE (KIE = 0.87) for CN-KCs arises from the combined contributions of these two pathways, rather than from a single dominant mechanism.

In our system, we estimate that approximately 42–55% of $H_2O_2$ is generated via the *O+*$H_2O$ pathway, while the remaining 45–58% proceeds through the conventional two-electron PCET pathway. Importantly, these two pathways exhibit fundamentally different isotope effects: the *O-mediated pathway does not involve direct O–H bond cleavage in the rate-determining step and can therefore exhibit a negligible or even inverse isotope effect, whereas the PCET pathway typically shows a normal KIE (>1), as also confirmed by the CN-K sample (KIE = 1.97), where the reaction proceeds almost exclusively via the PCET route.

Importantly, the overall KIE in such a system should be considered as a **weighted average of the contributions from the two pathways**, which can be expressed as:

$$\text{KIE}_{\text{obs}} = f_{\text{PCET}} \cdot \text{KIE}_{\text{PCET}} + f_{*O} \cdot \text{KIE}_{*O}$$

where $f_{\text{PCET}}$and $f_{*O}$represent the fractional contributions of the PCET and *O-mediated pathways, respectively.

It should be emphasized that the KIE of the PCET pathway on CN-KCs is expected

to be significantly smaller than that on CN-K, due to the reduced reaction barrier indicated by our DFT calculations. Indeed, recent literature reports that KIE values for PCET processes can be as low as ~1.04 (Nature Chemistry, 2024, 16, 1250–1260), depending on the reaction energetics and rate-determining step.

Therefore, when combining:

(i) a reduced KIE contribution from the PCET pathway, and

(ii) a *near-zero or inverse KIE contribution from the O-mediated pathway,

the resulting overall KIE can reasonably fall below 1. For example, assuming $f_{*O}$≈0.4, $KIE_{*O} \leq 1$, and a reduced $KIE_{PCET}$≈1.1, the weighted average can approach or even drop below 1, consistent with our experimental observation (KIE = 0.87).

Thus, the inverse KIE observed for CN-KCs is **net manifestation of parallel pathways with different isotope sensitivities**, rather than being inconsistent with the proposed mechanism.

**Q6.** Regarding response 10: First, I don't understand what is the basis for the two key premised in response 10. Second, in the case of *$^{16}O$ coupled with $H_2{}^{18}O$, the two premises is contradictory to each other. Third, based on the conclusion that 50-65% of the $H_2O_2$ is generated via PCET ($^{16}O_2$ + 2e- + 2H+ → $H_2{}^{16}O_2$), the highest peak should be the peak at m/z of 32 (the condition: $^{16}O_2$+ $H_2{}^{18}O$), which is contradictory to the results in Fig 4b and c. Forth, combined with the viewpoint that "when *16O is coupled with $H_2{}^{18}O$, the 16O atom dissociates more readily than 18O, the highest peak should rather be the peak at m/z of 32 than m/z of 36. Fifth, due to the smaller difference in atom weight of $^{16}O$ and $^{18}O$, the KIE is very close to 1. So, the dissociation rate difference between $^{16}O$ and $^{18}O$ is close.

**R6:** We sincerely thank the reviewer for this careful and detailed analysis. We appreciate the concerns raised regarding the interpretation of our isotopic experiments and agree that the previous explanation was not sufficiently rigorous and may lead to confusion.

First, we acknowledge that the interpretation based on the $MnO_2$ decomposition–GC-MS method relied on several simplifying assumptions regarding oxygen species distribution and did not adequately account for the complexity of oxygen scrambling. As the reviewer correctly pointed out, the coexistence and interconversion of $^{16}O_2$, $^{18}O_2$, and mixed $^{16}O$–$^{18}O$ species make it difficult to establish a well-defined relationship between the observed m/z signals and the actual reaction pathways. In this context, the two premises used in our previous response are indeed not strictly self-consistent.

Second, we recognize that the core issue lies in the limitation of the analytical method itself. During $MnO_2$-induced decomposition of $H_2O_2$, oxygen atom scrambling and recombination are unavoidable, leading to the formation of mixed isotopologues with non-defined ratios. As a result, the relative intensities of m/z = 32, 34, and 36

cannot be directly correlated to specific mechanistic pathways, and thus may lead to the discrepancy such as those highlighted by the reviewer.

To address this fundamental limitation, we have further adopted a more reliable isotopic tracing method based on **hydroxyl radical trapping using benzoic acid (BA) to form p-hydroxybenzoic acid (PHB)** “Water research, 2001, 35(2): 387-396.; Current Analytical Chemistry, 2012, 8, 143-149”. The use of benzoic acid as a probe for hydroxyl radicals is a well-established strategy in advanced oxidation processes, where ·OH reacts with benzoic acid to form hydroxylated products such as p-hydroxybenzoic acid (PHB), which can be quantitatively analyzed by chromatographic techniques. In this work, we extend this established approach by combining it with isotope labeling and GC–MS analysis to probe the oxygen origin in $H_2O_2$. Specifically, $H_2O_2$ is first converted into ·OH radicals via a Fenton-like process (1mM $Fe^{2+}$), and the resulting ·OH subsequently reacts with benzoic acid to form PHB. Importantly, the oxygen atom incorporated into PHB originates directly from $H_2O_2$, thereby preserving the isotopic information at the single-oxygen level. The extracted PHB was analyzed by GC–MS, where the isotopic composition can be directly determined based on the molecular ion peaks (m/z = 138 for $^{16}O$ and m/z = 140 for $^{18}O$). This approach avoids O–O bond reformation during analysis, could directly tracing the oxygen source at the atomic level and thus minimizes isotope scrambling effects associated with indirect detection methods.

Therefore, although this method is adapted from established hydroxyl radical trapping chemistry rather than being a standard isotopic protocol specifically designed for $H_2O_2$, it provides a reliable and chemically meaningful probe for evaluating the oxygen source and mechanistic interpretation. This approach enables **direct probing of the oxygen source at the single-atom level**, without involving O–O bond reformation steps. Therefore, it avoids the ambiguity associated with oxygen recombination and provides a more robust basis for mechanistic interpretation.

The detailed experimental procedures are as follows:

“To investigate the oxygen origin in $H_2O_2$, a benzoic acid (BA)-based hydroxyl radical trapping method combined with GC–MS analysis was employed.

After the photocatalytic reaction, an aliquot of the reaction solution containing $H_2O_2$ was collected. Benzoic acid (0.1 mM) was then added as a hydroxyl radical probe, followed by the introduction of $Fe^{2+}$ ions (0.2 mM) to initiate a Fenton-like reaction, generating hydroxyl radicals (·OH) from $H_2O_2$. The mixture was stirred at room temperature for 60 min to ensure sufficient conversion of BA into hydroxylated products, primarily p-hydroxybenzoic acid (p-HBA).

After the reaction, the mixture was extracted with ethyl acetate. The organic phase was collected, and concentrated under reduced pressure. The resulting products were

analyzed by gas chromatography-mass spectrometry (GC-MS). The isotopic composition of oxygen incorporated into p-HBA was determined by monitoring the molecular ion peaks:m/z = 138 corresponding to $^{16}O$-containing p-HBA; m/z = 140 corresponding to $^{18}O$-labeled p-HBA."

This method enables tracing the oxygen source in $H_2O_2$ at the single-oxygen level and minimizes isotope scrambling associated with indirect detection methods. The updated results are presented in Fig. R5 (corresponding to Fig. 4 in the revised manuscript). The related discussion is provided below, and the corresponding content has been updated in the main text accordingly.

"To further trace the source of oxygen in $H_2O_2$, isotope labeling experiments were conducted (Fig. 4a-4c, Supplementary Fig. 46). When $^{18}O_2$ was used instead of $O_2$, all catalysts produced $H_2O_2$ that could yield isotopically labeled $^{18}O$, via post-treatment with benzoic acid, into p-hydroxybenzoic acid (p-HBA) for GC-MS analysis (see details in Isotope labeling experiments). A clear shift of the molecular ion peak from m/z = 138 ($^{16}O$-containing p-HBA) to m/z=140 ($^{18}O$-labeled p-HBA) was observed, confirming that the oxygen atoms in $H_2O_2$ predominantly originate from $^{18}O_2$ via the conventional PCET pathway ($*^{18}O_2 + 2*H \rightarrow H_2{}^{18}O_2$) on CN-K. However, for CN-Cs and CN-KCs, in addition to the dominant m/z=140 peak, a noticeable signal at m/z=138 was also observed, with a relative intensity ratio of ~0.30-0.38 ($I_{138}/I_{140}$). This result suggests that a fraction of the produced $H_2O_2$ contains oxygen atoms not solely derived from $O_2$. This confirms the coexistence of an alternative pathway involving surface *O+ *$H_2O$ pathway, in which $*^{18}O$ couples with *$H_2O$ to form $H_2{}^{16}O^{18}O$ ($*^{18}O + *H_2O \rightarrow H_2{}^{16}O^{18}O$).

When replacing $H_2O$ with $H_2{}^{18}O$, the mass spectrometry peak for $H_2O_2$ using CN-K was only a peak at m/z=138. It can be concluded that the oxygen in $H_2O$ does not participate in $H_2O_2$ formation using CN-K, with all oxygen in $H_2O_2$ derived from $O_2$. This is consistent with the PCET pathway in which the *$O_2$ intermediate generates $H_2O_2$. In contrast, for CN-Cs and CN-KCs, an additional peak at m/z = 140 emerged, with a relative intensity ratio of ~0.26–0.34 ($I_{140}/I_{138}$). This observation indicates that part of the generated $H_2O_2$ incorporates oxygen from $H_2{}^{18}O$, resulting in mixed isotopic products ($H_2{}^{16}O^{18}O$). This can be attributed to the reaction $*^{16}O + H_2{}^{18}O \rightarrow H_2{}^{16}O^{18}O$. Taken together, these results consistently suggest that, in addition to the conventional PCET pathway, an alternative *O-mediated pathway ($*O + *H_2O \rightarrow H_2O_2$) operates on CN-Cs and CN-KCs.

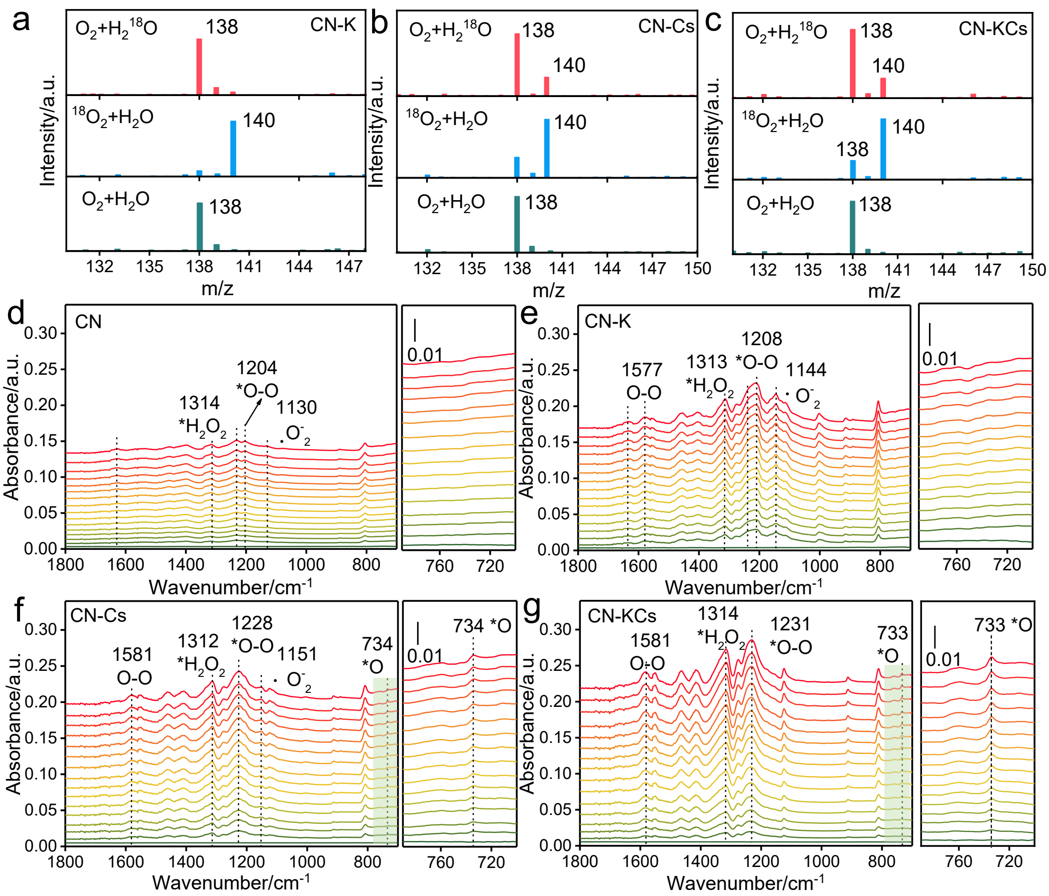

**Fig. R5. Investigation mechanism of $H_2O_2$ generation.** MS spectra of $O_2$ derived from $H_2O_2$ using $O_2$+$H_2O$, $^{18}O_2$+$H_2O$, and $O_2$+$H_2{}^{18}O$ on **a**. CN-K, **b**. CN-Cs and **c**. CN-KCs. In situ ATR-FTIR experiments for **d**. CN, **e**. CN-K, **f**. CN-Cs and **g**. CN-KCs under the visible light illumination, conducted under a $O_2$ gas flow. FTIR spectra were collected at 5-minute intervals.

Based on a semi-quantitative estimation from the isotopic peak ratios, we infer that approximately 42%–55% of $H_2O_2$ is generated via the *O + *$H_2O$ pathway, while the remaining fraction is produced through the conventional PCET route. This strongly supports that Cs atoms in CN serve as Lewis-acidic center, and thus facilitates the O-O bond dissociation to form *O, which could then directly couple with the adsorbed *$H_2O$ to form $H_2O_2$."

The detailed calculation process is as follows:

"To quantitatively determine the contribution of each pathway, we denote the molar fraction of $H_2O_2$ produced via the **$2e^-$ PCET pathway** as **x** and that via the **$1e^-$ *O + *$H_2O$** as **y**. The total $H_2O_2$ yield is normalized as x + y = 1.

In Experiment A **(**$^{18}O_2$ + $H_2{}^{16}O$**)**, the isotopic composition of the generated $H_2O_2$ is as follows:

- x·$H_2{}^{18}O_2$: Derived from the PCET pathway.
- y·$H_2{}^{18}O^{16}O$: Derived from the *O+ *$H_2O$ pathway, where one O atom originates

from $^{18}O_2$ and the other from $H_2{}^{16}O$.

Upon the addition of $Fe^{2+}$ for PHB detection, the O-O bond of $H_2O_2$ undergoes homolytic cleavage to generate·OH radicals. The distribution of the resulting·OH isotopes is:

1. From $H_2{}^{18}O_2$: Produces 2x moles of $^{18}OH$.
2. From $H_2{}^{18}O^{16}O$: Produces y moles of $^{18}OH$ and y moles of $^{16}OH$ (equimolar distribution).

The intensity of the MS peaks for $^{18}O$-PHB (m/z=140) and $^{16}O$-PHB (m/z=138) is proportional to the concentration of their respective ·OH precursors:

- $I(m/z=140) \propto 2x + y$
- $I(m/z=138) \propto y$

Based on the experimental intensity ratio $I(138)/I(140) \approx 0.34$, we have the following equation:

$y/(2x + y) = 0.34$

Substituting this into $x + y = 1$:

$y \approx 0.507$ (50.7%)

$x \approx 0.493$ (49.3%)

This derivation demonstrates that **50.7%** of the $H_2O_2$ incorporates one oxygen atom from the $H_2O$ solvent via the *O + *$H_2O$ pathway."

"In Experiment B **(**$O_2 + H_2{}^{18}O$**),** experimental intensity ratio $I(140)/I(130) \approx 0.30$, Based on a similar calculation formula, it can be derived that the *O + *$H_2O$ pathway accounts for approximately **46.2%**, while the PCET pathway accounts for approximately 53.8%.

- $I(m/z=138) \propto 2x + y$
- $I(m/z=140) \propto y$

Based on the experimental intensity ratio $I(140)/I(138) \approx 0.30$, we have the following equation:

$y/(2x + y) = 0.30$

Substituting this into $x + y = 1$:

$y \approx 0.462$ (46.2%)

$x \approx 0.538$ (53.8%)

This derivation demonstrates that **46.2%** of the $H_2O_2$ incorporates one oxygen atom from the $H_2O$ solvent via the *O + *$H_2O$ pathway."

This indicates that on the CN-KCs catalysts, about 46% of the $H_2O_2$ is generated via the single-electron pathway involving water oxidation. This result is highly consistent with the findings from Experiment A ($y \approx 50.7\%$), and such cross-validation significantly enhances the credibility of the proposed mechanism.

Based on this improved analysis, the isotopic distribution is consistent with our proposed mechanism, indicating that approximately 42–55% of $H_2O_2$ originates from

the *O+ *$H_2O$ pathway, while the remaining 45–58% is produced via the conventional PCET pathway." These results are also in good agreement with our KIE analysis, in situ spectroscopic observations, and DFT calculations.

We acknowledge that our previous interpretation relied on an indirect method and did not fully resolve this issue. The revised isotopic analysis provides a more robust and self-consistent validation of the proposed reaction pathways. We have accordingly revised the manuscript to clarify the limitations of the original method and to include the improved isotopic results. The using PHB method could provide a more reliable and self-consistent mechanistic understanding. We sincerely thank the reviewer for the insightful comments, which have helped us significantly improve the rigor and clarity of our work.

**Q7.** Regarding response 11: Please refer to comment 6.

**R7:** Please refer to **R6.**

**Q8.** Regarding response 13: The vibrations of oxygen species showed in Fig 4d-g should be also labeled in Fig R7 under $^{18}O$-labeled $O_2$ gas to prove the isotope effect.

**R8:** We appreciate the reviewer's professional suggestion. In accordance with your comments, we have labeled the oxygen species in the in-situ FTIR spectra under $^{18}O_2$ atmospheres to demonstrate the isotopic effect. As shown in the revised Fig. R6, the characteristic vibration bands of the adsorbed oxygen species exhibit a clear red-shift upon substituting $^{16}O_2$ with $^{18}O_2$:

The stretching vibration band of adsorbed oxygen species ($O_2$) originally located at 1581 $cm^{-1}$ in the $^{16}O_2$ system shifted to 1442 $cm^{-1}$ under $^{18}O_2$. The vibration signal of *O-O intermediates at 1231 $cm^{-1}$ exhibited a corresponding shift to 1144 $cm^{-1}$. The stretching vibration of the *$H_2O_2$ product at 1314 $cm^{-1}$ showed a discernible shift to 1254 $cm^{-1}$, further confirming the incorporation of isotopic oxygen into the final products. The *O stretching vibration shifted from 733 $cm^{-1}$ to 712 $cm^{-1}$, which is highly consistent with the theoretical isotope effect calculated using the harmonic oscillator model.

These experimental observations are in good agreement with the theoretical shifts calculated using the harmonic oscillator model, where the substitution of $^{16}O$ with $^{18}O$ typically results in a frequency reduction. These results provide robust evidence that the detected oxygen species and the produced $H_2O_2$ originate directly from the activation of gaseous $O_2$. We have updated Fig. R6 and the corresponding discussion in the revised manuscript.

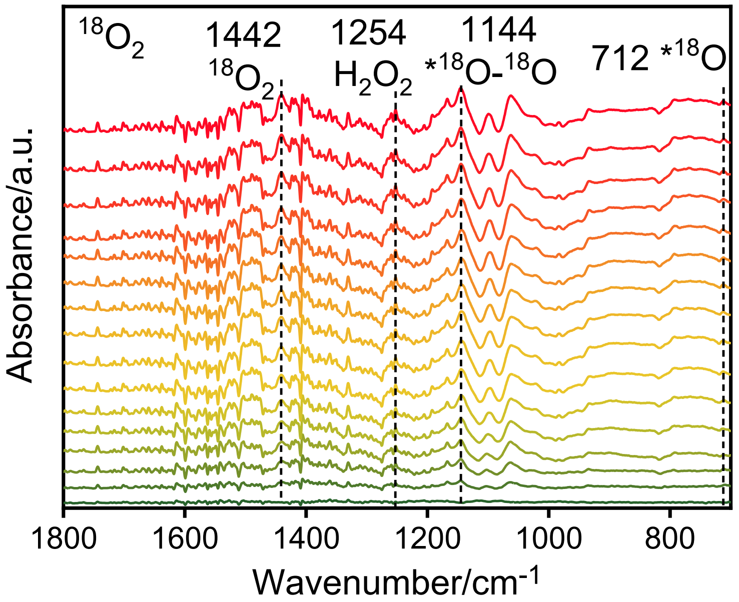


**Fig. R6.** In situ ATR-FTIR experiments for CN-KCs under visible light illumination, conducted under 18O-labeled $O_2$ gas. FTIR spectra were collected at 5-minute intervals.

**Q9.** Regarding response 16 and 17: The choice of reference energy shouldn't lead to the massive energy difference of "*+$H_2O_2$". The DFT calculation should be re-performed to give more reliable results.

**R9:** We sincerely thank the reviewer for the continued attention to this important point, which has helped us significantly improve the rigor and reliability of our theoretical analysis. We fully agree that, when the catalytic cycle is closed and referenced consistently, the free energy of the final state (* + $H_2O_2$) should be identical across different pathways. (including zero-point energy, entropy, and solvation contributions)

Upon carefully re-examining our previous calculations, we realize that the discrepancy did not originate from the DFT optimization itself, but rather from inconsistencies in the thermodynamic post-processing. Specifically, in the earlier version, different reaction pathways were constructed using slightly different combinations of reference states, and the corresponding corrections were not treated in a fully unified manner across all pathways. This led to an artificial offset in the absolute free energy of the final (* + $H_2O_2$) state.

Following the reviewer's suggestion, we have reprocessed and recalculated the reaction pathways using a fully consistent thermodynamic framework, in which:

- The pathways are referenced to the same initial state,
- The same set of correction terms (ZPE, entropy, solvation, and electrode potential where applicable) are applied consistently,

- And the total number of atoms and electrons are strictly conserved across the reaction cycles.

With this unified treatment, the free energy of the final (* + $H_2O_2$) state becomes identical across all pathways, shown in Fig.R7, as expected from thermodynamic principles. Importantly, we emphasize that this correction does not alter the rate-determining steps, or the overall mechanistic conclusions, but rather ensures internal thermodynamic consistency.

We acknowledge that the previous presentation may have caused confusion, and we have now fully revised the corresponding figures and descriptions in the manuscript to reflect the corrected and self-consistent results.

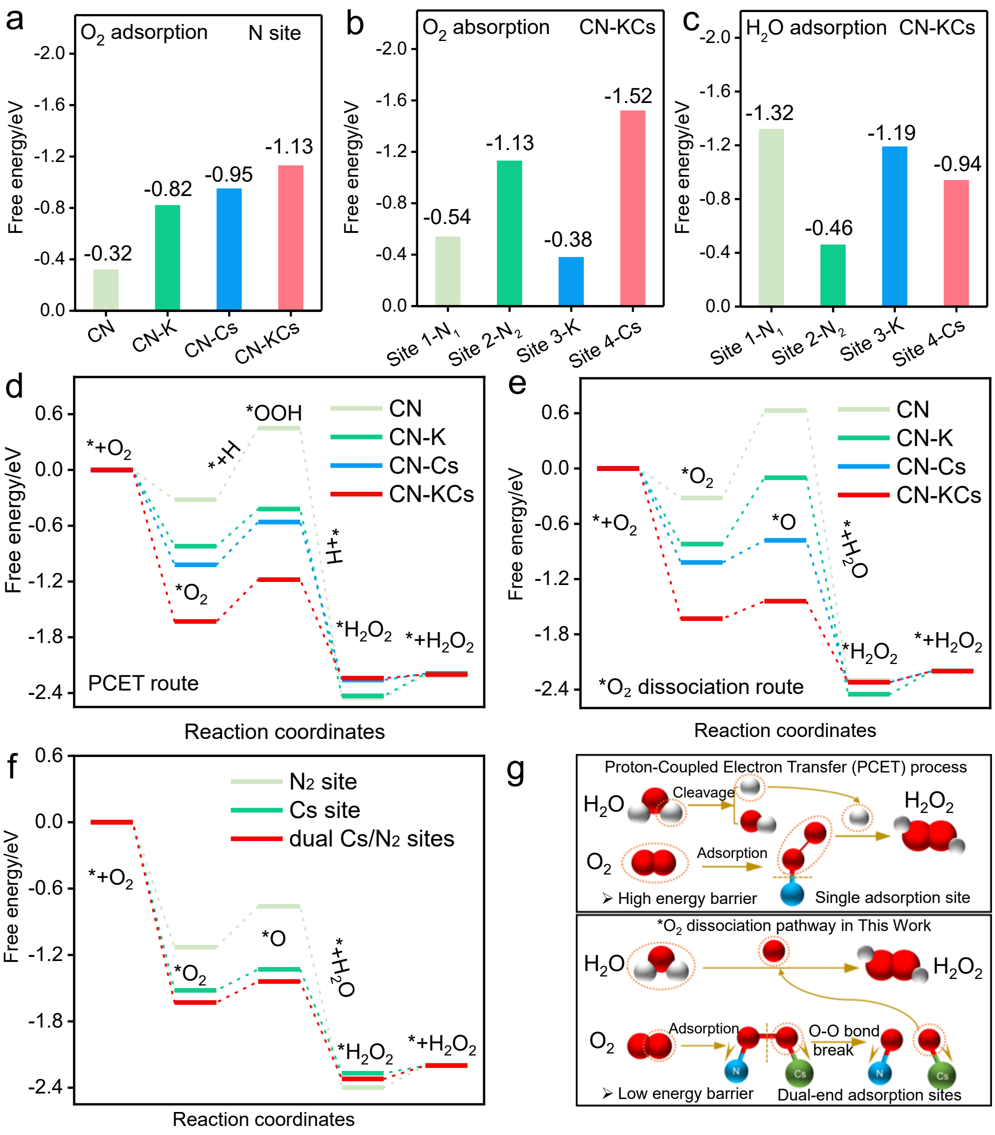


**Fig. R7. Investigation of energy barrier during $H_2O_2$ generation.** DFT Energy barrier for **a**. $O_2$ adsorption on catalysts. **b**. $O_2$ adsorption over different sites on CN-KCs and **c**. $O_2$ adsorption on catalysts. Calculated free energy diagram from $O_2$ to $H_2O_2$ via **d**. the PECT route or **e**. *$O_2$ dissociation pathway on different catalysts and **f**.

the different sites on CN-KCs. **g**. Proposed reaction mechanism for photocatalytic $H_2O_2$ production via the $*O_2$ dissociation pathway and comparison with the typical proton coupled electron transfer (PCET) process.

**Reviewer #3:** The revised manuscript by Liu and co-authors has been improved with substantial supplementary experiments added, which indeed are important to help understand the new pathway of cleavage of O–O for photocatalytic $H_2O_2$ generation. Nonetheless, I still have some concerns on the mechanism part as well as the explanations of experimental data.

**Q1.** For the isotopic experiments, I found that this experiment cannot give any insights in the reaction pathway due to the co-existence of $^{18}O_2$, $^{16}O_2$ and $^{18}O$-$^{16}O$. Particularly, these oxygen species do not have any specific ratio, making the analysis of the results difficult not to mention to explain the reaction mechanism. Note that if the experiment cannot prove the mechanism or provide the solid evidence for the reaction pathway, it is hence not self-consistent and convincing. The author explained this with several possibilities, but it didn't really solve the problem, particularly considering their explanation made based on several hypotheses.

**R1:** We sincerely thank the reviewer for this very constructive and forward-looking comment. We fully agree that isotopic labeling experiments can become ambiguous if oxygen scrambling occurs among different species ($^{16}O_2$, $^{18}O_2$, and mixed $^{16}O$–$^{18}O$), which may complicate mechanistic interpretation. Upon careful reassessment of our original approach, we recognize that the previously used method—decomposition of $H_2O_2$ into $O_2$ using $MnO_2$ followed by headspace GC-MS detection—has inherent limitations. Specifically, during the decomposition process, oxygen atom scrambling and recombination are unavoidable, leading to the formation of mixed isotopologues with non-defined ratios. As a result, this method does not reliably preserve the original isotopic signature of $H_2O_2$ and may therefore introduce uncertainty in mechanistic analysis.

To overcome this limitation, we have employed a more direct and reliable isotopic probing strategy based on **hydroxyl radical trapping using benzoic acid (BA)** to generate **p-hydroxybenzoic acid (PHB)** "Water research, 2001, 35(2): 387-396.; Current Analytical Chemistry, 2012, 8, 143-149". The use of benzoic acid as a probe for hydroxyl radicals is a well-established strategy in advanced oxidation processes, where ·OH reacts with benzoic acid to form hydroxylated products such as p-hydroxybenzoic acid (PHB), which can be quantitatively analyzed by chromatographic techniques. In this work, we extend this established approach by combining it with isotope labeling and GC–MS analysis to probe the oxygen origin in $H_2O_2$. Specifically, $H_2O_2$ is first converted into ·OH radicals via a Fenton-like process (1mM $Fe^{2+}$), and the

resulting ·OH subsequently reacts with benzoic acid to form PHB. Importantly, the oxygen atom incorporated into PHB originates directly from $H_2O_2$, thereby preserving the isotopic information at the single-oxygen level.

The extracted PHB was analyzed by GC–MS, where the isotopic composition can be directly determined based on the molecular ion peaks (m/z = 138 for $^{16}O$ and m/z = 140 for $^{18}O$). This approach avoids O–O bond reformation during analysis, could directly tracing the oxygen source at the atomic level and thus minimizes isotope scrambling effects associated with indirect detection methods.

Therefore, although this method is adapted from established hydroxyl radical trapping chemistry rather than being a standard isotopic protocol specifically designed for $H_2O_2$, it provides a reliable and chemically meaningful probe for evaluating the oxygen source and mechanistic interpretation. The detailed experimental procedures are as follows:

"To investigate the oxygen origin in $H_2O_2$, a benzoic acid (BA)-based hydroxyl radical trapping method combined with GC–MS analysis was employed.

After the photocatalytic reaction, an aliquot of the reaction solution containing $H_2O_2$ was collected. Benzoic acid (0.1 mM) was then added as a hydroxyl radical probe, followed by the introduction of $Fe^{2+}$ ions (0.2 mM) to initiate a Fenton-like reaction, generating hydroxyl radicals (·OH) from $H_2O_2$. The mixture was stirred at room temperature for 60 min to ensure sufficient conversion of BA into hydroxylated products, primarily p-hydroxybenzoic acid (p-HBA).

After the reaction, the mixture was extracted with ethyl acetate. The organic phase was collected, and concentrated under reduced pressure. The resulting products were analyzed by gas chromatography-mass spectrometry (GC-MS). The isotopic composition of oxygen incorporated into p-HBA was determined by monitoring the molecular ion peaks:m/z = 138 corresponding to $^{16}O$-containing p-HBA; m/z = 140 corresponding to $^{18}O$-labeled p-HBA."

This method enables tracing the oxygen source in $H_2O_2$ at the single-oxygen level and minimizes isotope scrambling associated with indirect detection methods. The updated results are presented in Fig. R5 (corresponding to Fig. 4 in the revised manuscript). The related discussion is provided below, and the corresponding content has been updated in the main text accordingly.

"To further trace the source of $H_2O_2$, isotope labeling experiments were conducted (Fig. 4a-4c, Supplementary Fig. 46). When $^{18}O_2$ was used instead of $O_2$, all catalysts produced $H_2O_2$ that could yield isotopically labeled $^{18}O$, via post-treatment with benzoic acid, into p-hydroxybenzoic acid (p-HBA) for GC-MS analysis (see details in Isotope labeling experiments). A clear shift of the molecular ion peak from m/z = 138 ($^{16}O$-containing p-HBA) to m/z=140 ($^{18}O$-labeled p-HBA) was observed, confirming that the

oxygen atoms in $H_2O_2$ predominantly originate from $^{18}O_2$ via the conventional PCET pathway ($*^{18}O_2 + 2*H \rightarrow H_2{}^{18}O_2$). However, for CN-Cs and CN-KCs, in addition to the dominant m/z=140 peak, a noticeable signal at m/z=138 was also observed, with a relative intensity ratio of ~0.30-0.38 ($I_{138}/I_{140}$). This result suggests that a fraction of the produced $H_2O_2$ contains oxygen atoms not solely derived from $O_2$. Such behavior can be rationalized by the coexistence of an alternative pathway involving surface *O+ *$H_2O$ pathway, in which *$^{18}O$ couples with *$H_2O$ to form $H_2{}^{16}O^{18}O$ ($*^{18}O + *H_2O \rightarrow H_2{}^{16}O^{18}O$).

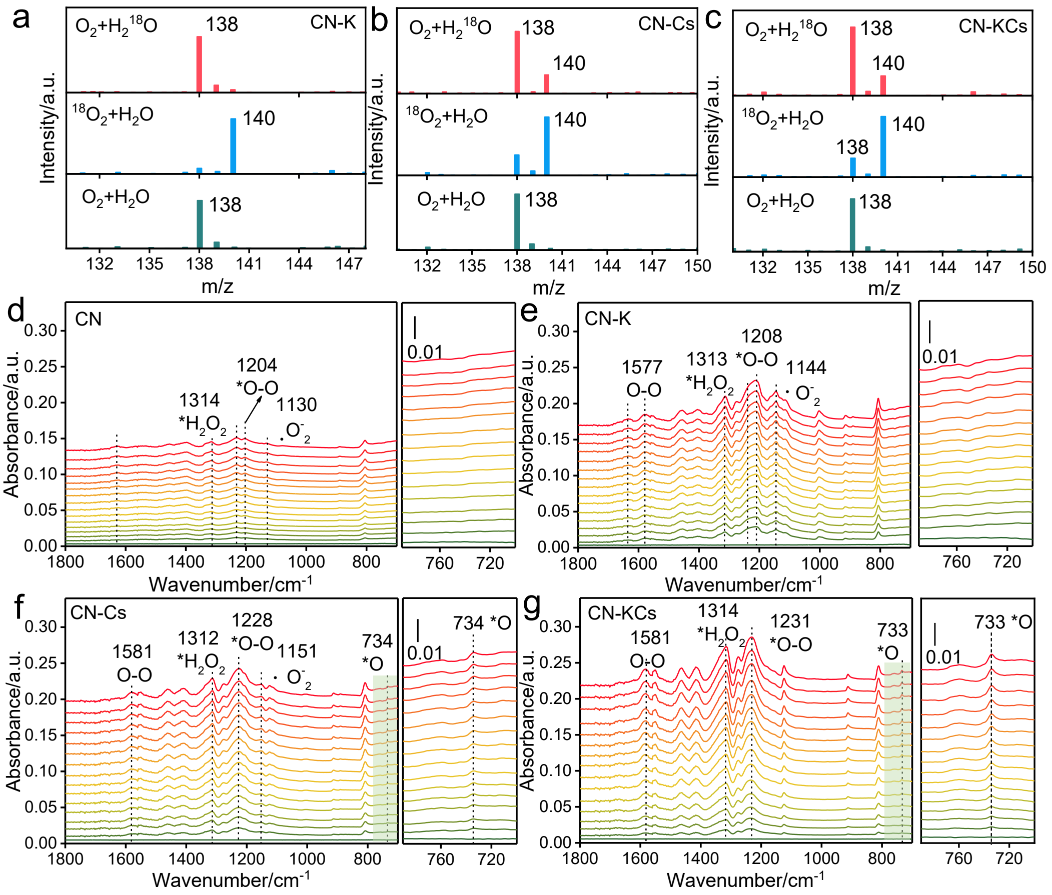


**Fig. R5. Investigation mechanism of $H_2O_2$ generation.** MS spectra of $O_2$ derived from $H_2O_2$ using $O_2$+$H_2O$, $^{18}O_2$+$H_2O$, and $O_2$+$H_2{}^{18}O$ on **a**. CN-K, **b**. CN-Cs and **c**. CN-KCs. In situ ATR-FTIR experiments for **d**. CN, **e**. CN-K, **f**. CN-Cs and **g**. CN-KCs under the visible light illumination, conducted under a $O_2$ gas flow. FTIR spectra were collected at 5-minute intervals.

When replacing $H_2O$ with $H_2{}^{18}O$, the mass spectrometry peak for $H_2O_2$ using CN-K was only a peak at m/z=138. It can be concluded that the oxygen in $H_2O$ does not participate in $H_2O_2$ formation using CN-K, with all oxygen in $H_2O_2$ derived from $O_2$. This is consistent with the PCET pathway in which the *$O_2$ intermediate generates $H_2O_2$. In contrast, for CN-Cs and CN-KCs, an additional peak at m/z = 140 emerged, with a relative intensity ratio of ~0.26–0.34 ($I_{140}/I_{138}$). This observation indicates that part of

the generated $H_2O_2$ incorporates oxygen from $H_2{}^{18}O$, resulting in mixed isotopic products ($H_2{}^{16}O^{18}O$). This can be attributed to the reaction $*^{16}O + H_2{}^{18}O \rightarrow H_2{}^{16}O^{18}O$. Taken together, these results consistently suggest that, in addition to the conventional PCET pathway, an alternative *O-mediated pathway ($*O + *H_2O \rightarrow H_2O_2$) operates on CN-Cs and CN-KCs.

Based on a semi-quantitative estimation from the isotopic peak ratios, we infer that approximately 42%–55% of $H_2O_2$ is generated via the $*O + *H_2O$ pathway, while the remaining fraction is produced through the conventional PCET route. We note that this estimation is intended to provide a qualitative assessment of pathway contributions rather than an exact quantification. These it can be inferred that Cs atoms in CN serve as Lewis-acidic center, and thus facilitates the O-O bond dissociation to form *O, which could then directly couple with the adsorbed $*H_2O$ to form $H_2O_2$."

The detailed calculation process is as follows:

"To quantitatively determine the contribution of each pathway, we denote the molar fraction of $H_2O_2$ produced via the **2e⁻ PCET pathway** as **x** and that via the **1e⁻ *O + *$H_2O$** as **y**. The total $H_2O_2$ yield is normalized as x + y = 1.

In Experiment A (**$^{18}O_2 + H_2{}^{16}O$**), the isotopic composition of the generated $H_2O_2$ is as follows:

- $x \cdot H_2{}^{18}O_2$: Derived from the PCET pathway.
- $y \cdot H_2{}^{18}O^{16}O$: Derived from the *O+ *$H_2O$ pathway, where one O atom originates from $^{18}O_2$ and the other from $H_2{}^{16}O$.

Upon the addition of $Fe^{2+}$ for PHB detection, the O-O bond of $H_2O_2$ undergoes homolytic cleavage to generate·OH radicals. The distribution of the resulting·OH isotopes is:

3. From $H_2{}^{18}O_2$: Produces 2x moles of $^{18}OH$.
4. From $H_2{}^{18}O^{16}O$: Produces y moles of $^{18}OH$ and y moles of $^{16}OH$ (equimolar distribution).

The intensity of the MS peaks for $^{18}O$-PHB (m/z=140) and $^{16}O$-PHB (m/z=138) is proportional to the concentration of their respective ·OH precursors:

- $I(m/z=140) \propto 2x + y$
- $I(m/z=138) \propto y$

Based on the experimental intensity ratio $I(138)/I(140) \approx 0.34$, we have the following equation:

$y/(2x + y) = 0.34$

Substituting this into x + y = 1:

$y \approx 0.507$ (50.7%)

$x \approx 0.493$ (49.3%)

This derivation demonstrates that **50.7%** of the $H_2O_2$ incorporates one oxygen atom

from the $H_2O$ solvent via the *O + *$H_2O$ pathway."

"In Experiment B **($O_2$ + $H_2{}^{18}O$),** experimental intensity ratio I(140)/I(130)≈0.30, Based on a similar calculation formula, it can be derived that the *O + *$H_2O$ pathway accounts for approximately **46.2%**, while the PCET pathway accounts for approximately 53.8%.

- $I(m/z=138) \propto 2x + y$
- $I(m/z=140) \propto y$

Based on the experimental intensity ratio I(140)/I(138)≈0.30, we have the following equation:

$y/(2x + y) = 0.30$

Substituting this into x + y = 1:

$y \approx 0.462$ (46.2%)

$x \approx 0.538$ (53.8%)

This derivation demonstrates that **46.2%** of the $H_2O_2$ incorporates one oxygen atom from the $H_2O$ solvent via the *O + *$H_2O$ pathway."

This indicates that on the CN-KCs catalysts, about 46% of the $H_2O_2$ is generated via the single-electron pathway involving water oxidation. This result is highly consistent with the findings from Experiment A (y≈50.7%), and such cross-validation significantly enhances the credibility of the proposed mechanism.

Based on this improved analysis, the isotopic distribution is consistent with our proposed mechanism, indicating that approximately 42–55% of $H_2O_2$ originates from the *O+ *$H_2O$ pathway, while the remaining 45–58% is produced via the conventional PCET pathway."

We acknowledge that our previous interpretation relied on an indirect method and did not fully resolve this issue. The revised isotopic analysis provides a more robust and self-consistent validation of the proposed reaction pathways. We have accordingly revised the manuscript to clarify the limitations of the original method and to include the improved isotopic results. We sincerely appreciate the reviewer's insightful comment, which has significantly strengthened the rigor and reliability of our work.

**Q2.** In-situ FTIR showed the signals of *O, which are very weak with low S/N ratio to distinguish them. Because this signal is key evidence to identify the reaction mechanism, I wish they can reconsider the experimental procedures and redo the measurement to provide the observable signals at least.

**R2:** We thank the reviewer for this valuable and constructive suggestion. We agree that the signal corresponding to *O is relatively weak with a low signal-to-noise (S/N) ratio, and we appreciate the reviewer's emphasis on its importance for mechanistic identification.

Following the reviewer's recommendation, we have repeated the in situ ATR-FTIR

measurements under the same and optimized conditions. However, we found that the S/N ratio of the *O signal remains comparable to that shown in Fig. R8, without significant improvement. We believe this is intrinsic to the nature of the *O intermediate and the spectral region, rather than solely due to experimental limitations. Specifically, the relatively weak *O signal can be attributed to the following factors:

(1) Spectral region limitation:

The *O signal appears around ~733 $cm^{-1}$, which lies in the fingerprint region of IR spectra, where absorption features are generally weaker and often overlap with background vibrations, making high-intensity signals difficult to obtain.

(2) *Low surface coverage of O intermediate:

The *O species is highly reactive and is rapidly consumed in subsequent reaction steps (e.g., coupling with $H_2O$), preventing its accumulation to a high steady-state concentration on the catalyst surface, and thus resulting in a relatively weak IR signal.

(3) Wide absorbance scale used in the spectra:

The presented spectra cover a relatively broad absorbance range (0–0.35) could reduces the apparent intensity of the *O peak. Nevertheless, as shown in the inset (-0.1 ) the *O signal can still be clearly distinguished and exhibits a consistent evolution with increasing illumination time, supporting its assignment.

Importantly, we note that the identification of the *O intermediate is not based on a single piece of evidence, but is **corroborated by multiple complementary techniques**, including TPD analysis, KIE results, and DFT calculations, all of which consistently support the proposed O–O dissociation pathway.

We acknowledge the reviewer's concern and will continue to explore improved experimental approaches to further enhance the signal quality.

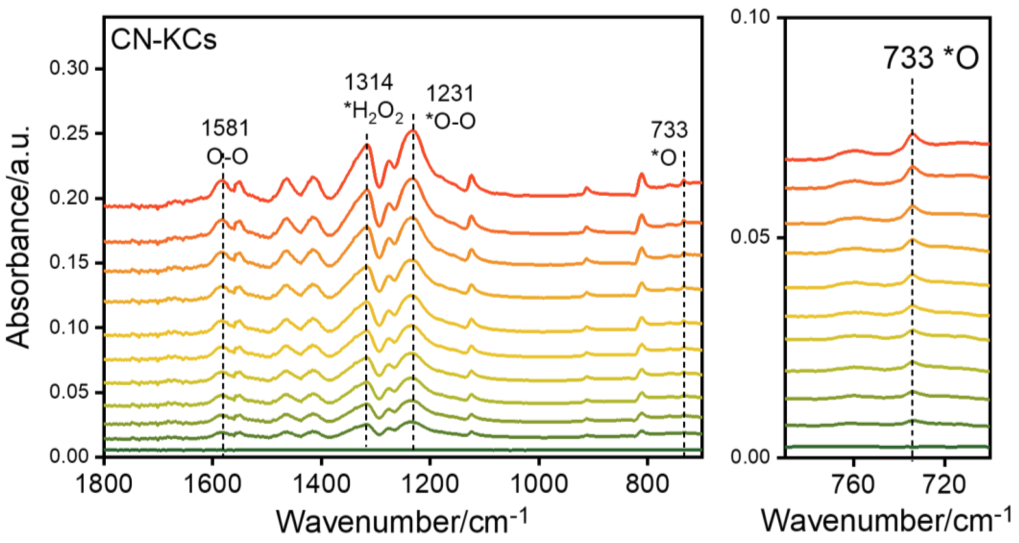


**Fig. R8.** In situ ATR-FTIR experiments for CN-KCs under the visible light illumination, conducted under a $O_2$ gas flow. FTIR spectra were collected at 5-minute intervals.

**Q3.** Another must be mentioned is the processing procedure of isotope labelling experiment. How to remove ethylene glycol and its derivatives from the reaction system is unknown. The author did not provide any experimental details. Note that these components are likely to have signals in mass spectrum but absent in their results. These derivatives are hydrophilic and mix up with water, fully removing these compounds from water is impossible.

**R3:** We appreciate the reviewer's professional caution regarding potential interference from ethylene glycol (EG) and its derivatives in the mass spectrometry (MS) analysis. We apologize for the insufficient detail in the original manuscript and would like to clarify our experimental procedure, which was specifically designed to avoid such liquid-phase interference.

Using the $^{18}O_2$ labeling experiment as an example, the refined procedure is as follows:

1. Reaction Setup: 15 mg of the photocatalyst was dispersed in 20 mL of a 0.5% (v/v) EG aqueous solution within a sealed flat quartz reactor (total volume 50 mL). The suspension was sonicated for 10 minutes to ensure uniform dispersion.
2. Isotope Introduction & Reaction: After purging the system with $^{18}O_2$, the reaction was conducted using the PCX-50C Discover Multi-channel system.
3. Purification & Gas Removal: Post-reaction, the suspension was centrifuged to remove the CN-KCs catalyst. The remaining supernatant was transferred to a 25 mL sealed glass vial. An AC1000 Atmosphere Controller was employed to perform multiple vacuum-argon cycles to ensure the complete removal of dissolved $^{18}O_2$, which was confirmed via GC-MS.
4. $H_2O_2$ Decomposition & Headspace Sampling: Inside an Ar-filled glovebox, 0.2 g of $MnO_2$ was added to the vial. The mixture was stirred for 10 minutes to quantitatively decompose the produced $H_2O_2$ into $O_2$ gas ($H_2O_2 \rightarrow 2H_2O + O_2$).
5. Analysis: Crucially, we performed **headspace sampling** using a gas-tight syringe. The needle was inserted only into the top gas phase of the vial and did not contact the liquid phase.

Since the MS analysis targets the gaseous $O_2$ generated from $H_2O_2$ decomposition rather than the liquid solution, the non-volatile ethylene glycol and its hydrophilic derivatives remain in the liquid phase and do not enter the GC-MS system. This sampling method effectively eliminates the risk of signals from EG derivatives interfering with the isotope results. We have added these details to the Experimental Section of the revised manuscript.

**Q4.** Supplementary Fig. 7 showed the NMR spectrum of the oxidative product from EG oxidation, but no product peaks were identified. Please assign the characteristic peaks from acid, aldehyde, etc.

**R4:** We thank the reviewer for this constructive suggestion. In accordance with your comments, we have assigned the characteristic peaks corresponding to the glycolic acid and glycolaldehyde products of ethylene glycol (EG) oxidation (Fig.R9).

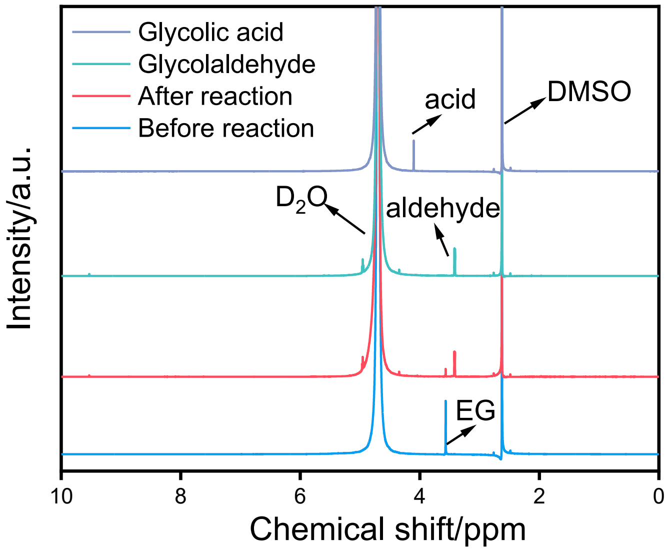


**Fig. R9.** The NMR spectrum of the oxidative product from EG oxidation, for the NMR analysis, 0.7 ml target liquid sample, 50 μL $D_2O$ and 10 ul of DMSO.

**Q5.** The performance comparison with reported literature was presented in Fig 1g but was unfair. The authors utilized sacrificial reagents in both QE and SCC measurements, which did not reflect the real superiority of their photocatalyst. The testing conditions should be identical with these literatures.

**R5:** We appreciate the reviewer's insightful comment regarding the fairness of the performance comparison. We agree that reaction conditions, particularly the use of sacrificial agents or oxidation substrates, significantly influence the apparent performance (SCC and QE). However, since the reported literature employs a wide variety of reaction systems, it is practically challenging to achieve identical testing conditions for all compared catalysts. To address your concern and provide a more fair comparison, we have revised Fig. 1g (i.e. Fig. R10) by categorizing the reported works into two distinct groups based on their reaction media:

1) Pure Water Systems: Represented by light green circles, these data points refer to photocatalytic $H_2O_2$ production in pure water without any additional substrates.

2) Substrate-containing Systems: Represented by deep blue circles, these refer to systems where oxidation substrates (e.g., biomass derivatives or sacrificial agents) were added alongside $H_2O$.

By differentiating these two systems, we aim to provide a clearer benchmark for our catalyst's performance under its specific operating conditions while acknowledging the inherent differences in reaction pathways. The updated figure and the corresponding

discussion have been incorporated into the revised manuscript.

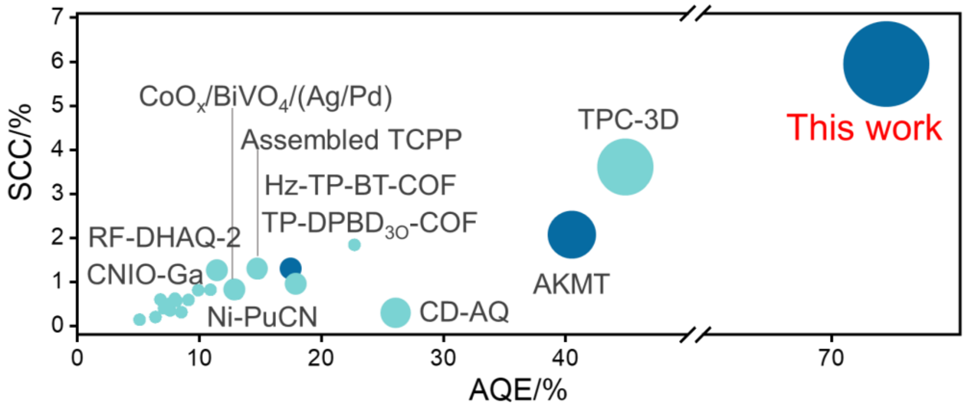


**Fig. R10.** Summarized photocatalytic $H_2O_2$ synthesis with the AQE and SCC value. It should be noted that the SCC reported here just reflects the efficiency of converting solar energy into the chemical energy stored in $H_2O_2$, under assisted conditions, rather than a closed-cycle solar fuel efficiency. Light green circles, signifying the pure water reaction system, and deep blue circles, signifying the oxidative substrate added.

**Q6.** Another bug is the QE calculation based on the 2e⁻ ORR pathway that did not occur in this reaction. The hypothesis of two electrons transfer is not suitable as the variation of chemical valence in O of $H_2O$ is one. The QE calculation was therefore not correct. Additionally, the experimental parameters including light intensity, distance, irradiation area should be provided.

**R6:** We sincerely appreciate the reviewer's professional insight regarding the electron transfer stoichiometry. You are correct that assuming a uniform $2e^-$ pathway is imprecise if a portion of the $H_2O_2$ originates from the $O_2$ reduction involving a valence change of one. Following your constructive suggestion, we have carefully re-calculated the Quantum Efficiency (QE) by considering the co-existence of different reaction pathways. Based on our mechanistic studies, it is estimated that approximately 42%–55% of the $H_2O_2$ is generated via the*O + *$H_2O$ pathway (a single-electron transfer process), while the rest is produced through the conventional $2e^-$ proton-coupled electron transfer (PCET) pathway. For the revised calculation, we used a weighted average approach, assuming that 48.5% of the $H_2O_2$ yield follows the $1e^-$ pathway and 51.5% follows the $2e^-$ PCET pathway. Accordingly, we have updated the QE data (Fig. R4) and the corresponding figures (Fig. 1b and Fig. 1g) in the revised manuscript.

The detailed experimental parameters and the revised calculation process (using λ=420 nm as an example) are provided below:

The QE for $H_2O_2$ production was measured using a PCX-50C Discover Multi-channel photochemical reaction system.Light Source: Different monochromatic wavelengths of light (λ) including 385±2 nm, 420±2 nm, 450±2 nm, 485±2 nm, 535±2 nm, 595±2 nm

and 630±2 nm was used as the light source.

Irradiation image: as shown in Fig. R11, the reactor was illuminated from the bottom. The light source was positioned in close proximity to the base of the reaction vessel to minimize energy loss.

Light Intensity (I): λ = 420 nm, the incident light intensity was measured as 12.5 mW/cm$^2$.

Irradiation Area (S): The reactor's illumination window has a diameter of approximately 3.5 cm, resulting in an effective irradiation area (S) of 9.616 cm$^2$.

The number of reacted electrons ($N_e$) is no longer simply 2×$N_{H2O2}$. Instead, it is calculated as follows: $N_e$=2×PCET yield of $H_2O_2$ (51.5%) +1×single electron of $H_2O_2$ (48.5%).

"To calculate the AQE at the wavelength of 420±2 nm on using CN-KCs, the calculated process as follows:

$$N_e\text{=number of reacted electrons (mols}^{-1}\text{)}$$

$$=2\times\frac{n2}{t}+1\times\frac{n1}{t}=\frac{(2\times372.83+1\times351.12)\times10^{-6}}{3600}=3.047\times10^{-7}\ \text{mols}^{-1}$$

The number of $H_2O_2$ molecules from the production rate of $H_2O_2$ under 420 nm was calculated to be 723.95 μmol h$^{-1}$. Accordingly, the $H_2O_2$ generated via the single-electron reduction pathway is 351.12 μmol h$^{-1}$ (723.95×48.5%), while that produced through the two-electron proton-coupled electron transfer (PCET) process is 372.83 μmol h$^{-1}$. Then, the received reacted electron number of the $H_2O_2$ products ($N_e$) is measured as (2×372.83+1×351.12)×10$^{-6}$/3600=3.047×10$^{-8}$ mols$^{-1}$.

The energy of a single photon ($E_s$) at λ = 420 nm is calculated according to hc/λ, where h and c are the Planck constant 6.63×10$^{-34}$ J s and the speed of light 3×10$^8$ m/s, respectively, $E_s$ was found to be 4.736×10$^{-19}$ J.

$$E_s=\frac{hc}{\lambda}=\frac{6.63\times10^{-34}\times3\times10^{8}}{4.2\times10^{-7}}=4.736\times10^{-19}\ \text{J}$$

The Avogadro's number (denoted as A) representing the number in one mole of a substance is 6.022 × 10$^{23}$. The incident light intensity (I) was measured as 12.5 mW/cm$^2$, calibrated by a light power meter (PL-MW2000). The reactor's illumination window, approximately 3.5 cm in diameter, yielded an irradiation area (S) of 9.616 cm$^2$. Consequently, the total number of photons incident (Np) offered in the reaction vessel per hour was calculated to be 4.214×10$^{-7}$ mol s$^{-1}$.

$$N_p=\frac{I\times S}{E_s\times A}=\frac{12.5\times10^{1}\times9.616\times10^{-4}}{4.736\times10^{-19}\times6.022\times10^{23}}=4.214\times10^{-7}$$

Thus, according to equation (1), the AQE (%) =72.3%.

$$AQE(\%)=\frac{N_e}{N_P}=\frac{\text{number of reacted electrons}}{\text{number of incident photons}}\times100\%$$

$$=\frac{3.047\times10^{-7}}{4.214\times10^{-7}}\times100\%=72.3\%$$

**Take AQY at the wavelength of 385±2 nm of CN-KCs as an other example,**

The number of $H_2O_2$ 744.8molecules from the production rate of $H_2O_2$ under 385 nm was calculated to be 352.32 μmol $h^{-1}$. Accordingly, the $H_2O_2$ generated via the single-electron reduction pathway is 170.88 μmol $h^{-1}$ (352.32×48.5%), while that produced through the two-electron proton-coupled electron transfer (PCET) process is 181.44 μmol $h^{-1}$. Consequently, the total number of reacted electrons for the $H_2O_2$ products ($N_e$) is determined to be $(2\times181.44+1\times170.88)\times10^{-6}/3600=1.483\times10^{-7}$ $mols^{-1}$.

$$N_e=\text{number of reacted electrons } (mols^{-1})$$

$$=2\times\frac{n2}{t}+1\times\frac{n1}{t}=\frac{(2\times181.44+1\times170.88)\times10^{-6}}{3600}=1.483\times10^{-7}\ mols^{-1}$$

The energy of a single photon ($E_s$) at λ = 385 nm is calculated to be $5.234\times10^{-19}$ J, the light intensity (I) was measured as 6.5 mW/$cm^2$. Consequently, the total number of photons incident (Np) offered in the reaction vessel per hour was calculated to be $1.983\times10^{-7}$ mol $s^{-1}$.

$$N_p=\frac{I\times S}{E_s\times A}=\frac{6.5\times10^{1}\times9.616\times10^{-4}}{5.234\times10^{-19}\times6.022\times10^{23}}=1.983\times10^{-7}$$

Thus, according to equation (1), the AQE (%) =74.8%.

$$AQE(\%)=\frac{N_e}{N_P}=\frac{\text{number of reacted electrons}}{\text{number of incident photons}}\times100\%$$

$$=\frac{1.483\times10^{-7}}{1.983\times10^{-7}}\times100\%=74.8\%$$

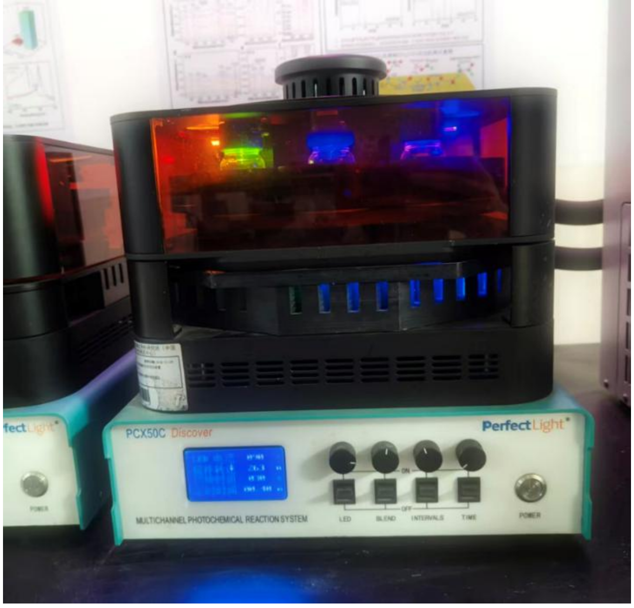


**Fig. R11.** The digital image of the photoreaction setup of calculation the QE value.

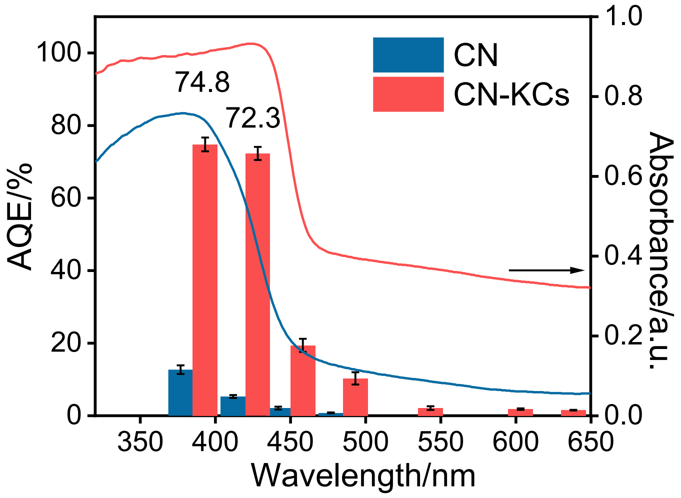


**Fig. R4.** Apparent quantum efficiencies (AQE) for $H_2O_2$ product at specific incident wavelengths, together with the DRS spectra of CN and CN-KCs.

**Reviewer #4:** The authors have signficantly largely addressed my concerns with the manuscript. I only have two remaining concerns:

**Q1.** In the abstract, it should be clearly stated that the SCC $H_2O_2$ efficiency is obtained in the presence of a sacrificial electron donor.

**R1:** We appreciate the reviewer's constructive suggestion. The abstract has been revised accordingly to clearly state that the SCC $H_2O_2$ efficiency is obtained in the presence of a sacrificial electron donor.

**Q2.** In the discussion of the uf data, the statement 'density of shallow trap states is

clearly enhanced' is not, as far as I can see, clear from the data and should be deleted.

**R2:** We appreciate the reviewer's insightful comment. Upon careful re-examination of the data, we agree that this conclusion is not sufficiently supported. Therefore, the related statement has been removed from the revised manuscript.
